# Regulatory Territory and General Deterrence Across Borders: Swiss Banks’ Territorial Self-Categorizations and Responses to U.S. Extraterritorial Law Enforcement

**DOI:** 10.1177/00018392251334366

**Published:** 2025-05-07

**Authors:** Emmanuelle Reuter, Florian Überbacher, Andreas Georg Scherer

**Affiliations:** 1 University of Neuchâtel; 2 MBS School of Business; 3 University of Zurich

**Keywords:** social control, wrongdoing, coercion, deterrence, rational choice, neo-institutionalism, culture, schemas, categories, space, territory, transnational governance, international business, international law, financial regulation, private wealth management, Swiss banking industry, tax evasion

## Abstract

Regulators increasingly engage in extraterritorial law enforcement, but its deterrence effects on organizations remain poorly understood. Based on a case study in the Swiss private wealth management industry, we explore the conditions under which U.S. extraterritorial law enforcement provoked cross-border general deterrence, preventing not only the prosecuted Swiss banks but also the unprosecuted ones (i.e., the observers of the events) from violating U.S. law when a peer was prosecuted for similar behavior. Our inductive analysis led us to integrate deterrence theory with a novel cultural–cognitive, organization-centered perspective on *regulatory territory:* the spatial scope of a regulator’s jurisdictional authority. This integration suggests that unprosecuted banks’*territorial self-categorization*—a form of spatial self-categorization that leads them to conclude whether they are located inside or outside what they perceive to be a foreign regulator’s territory—is critical for explaining cross-border general deterrence. Our findings emphasize the significance of the clarity with which the U.S. regulator communicated its regulatory territory and how different types (which we call locals and cosmopolitans) of organizations’ taken-for-granted categorization schemes influenced their rational choices and instrumental actions. We contribute to scholarship on organizations’ legal environment and to organizational research on categories.

Deterrence is a coercive mechanism that stresses the role of law enforcement and sanctioning to ensure organizations’ compliance with national laws (e.g., Edelman & Suchman, 1997; Reid & Toffel, 2009; Short & Toffel, 2010). Law enforcement represents a social event that is interpreted by individuals and organizations and that may deter both those who are prosecuted (*specific deterrence*) and those who are not prosecuted (*general deterrence*) (Gray & Shimshack, 2011; Tan & West, 2023; Thornton et al., 2005; Yiu et al., 2014), i.e., the observers of the events. Based on a rational choice perspective (Becker, 1968), with its logic of consequences and focus on instrumental perceptions and actions (Scott, 2014), the deterrence literature suggests that unprosecuted organizations’ perception of the severity of sanctions resulting from a prosecution is a necessary but insufficient condition for deterring them from illegal behaviors (Paternoster & Bachman, 2012). Of particular relevance is these organizations’ perceived threat of prosecution: the realization of a threat of criminal charges or legal sanctions given the violation of legal rules (e.g., Andenaes, 1966; Nagin, 2013).

Following the territoriality principle of international law (e.g., Ryngaert, 2015), research on organizations’ legal environment generally assumes that the spatial scope of where a state has the capacity to enforce national law is fixed, mutually exclusive, and geographically defined by state borders (Edelman & Suchman, 1997; Schneiberg & Bartley, 2008; Vogel, 2008). Thus, states are presumed to lack the regulatory power to prosecute individuals or organizations for violations of national laws abroad (e.g., Djelic & Quack, 2018; Kobrin, 2001). Legal coercion and deterrence have therefore been explored only within (not across) national borders (Marx, 1997). Yet, a few states (and supranational regulators, like the European Commission) with sufficient coercive power are increasingly engaging in *extraterritorial* law enforcement (e.g., Kaczmarek & Newman, 2011; Parrish and Ryngaert, 2023; Putnam, 2009; Überbacher & Scherer, 2020; Woll, 2023): a unilateral transnational governance type (Krisch, 2022) that entails enforcing national laws against private actors (companies and their agents) for transgressing these laws beyond the regulator’s national borders, without prior consent to the enforcement from the targeted country’s government (e.g., Kalyanpur & Newman, 2018).^1^

However, the deterrence effects of extraterritorial law enforcement have hardly been studied. We do not know whether extant deterrence theory, with its focus on rational choice and instrumental action, can sufficiently explain general deterrence in cross-border settings or whether other, less instrumental mechanisms play a role. The neo-institutional literature has long emphasized that organizations are culturally and socially embedded rather than atomistic actors and that a logic of appropriateness, emphasizing interpretations and taken-for-granted beliefs, may strongly impact their legal compliance, particularly when the regulator communicates in ambiguous and unclear ways (e.g., Edelman, 1992, 2016; Suchman, 1997; Sutton & Dobbin, 1996). However, the deterrence and the neo-institutional literatures have evolved largely separately and, despite repeated calls (e.g., Edelman & Suchman, 1997; Short, 2013; Vaughan, 1998), still await substantial integration. Thus, the role of cultural–cognitive factors in shaping unprosecuted domestic organizations’ interpretations of extraterritorial enforcement events and prosecution threat perceptions remains understudied.

To address this shortcoming, we conducted a comparative case study of legal violations in Swiss private wealth management (PWM), wherein Swiss banks were prosecuted for allegedly conspiring with their U.S. clients to defraud the U.S. government via tax evasion. We sought to explain why the general deterrence effects of two extraterritorial law enforcement events by U.S. regulators varied in a population of unprosecuted Swiss banks that engaged in illegal behaviors similar to those of the prosecuted banks. Deterrence theory cannot sufficiently explain our findings. The U.S. regulators’ coercive power and the severity of sanctions levied (very high and undisputed by the banks in both events) were necessary but insufficient factors for explaining the observed variations in unprosecuted Swiss banks’ compliance. Also, extant research on triggers of unprosecuted organizations’ perceptions of prosecution threat and other alternative explanations cannot sufficiently explain these differences.

To more fully explain cross-border general deterrence, our qualitative analysis led us to elaborate deterrence theory by providing a novel cultural–cognitive perspective on regulatory territory. We blended research on territory from international relations and international law (e.g., Buxbaum, 2009; Sassen, 2013) with neo-institutional research on categories and categorization (DiMaggio, 1997; Durand et al., 2017; Glynn & Navis, 2013).

As studied in international relations and international law (e.g., Buxbaum, 2009; Sassen, 2013), territory is the spatial scope of an actor’s power (Storey, 2012). Here, we define it more narrowly as the spatial scope of a regulator’s jurisdictional authority. The territory literature conceives of a regulator’s territory as dependent on the territory claims made and enforced by the regulator (e.g., Buxbaum, 2009). According to the literature on categories, these claims can be conceived of as entailing *territorial categories*, that is, nomenclatures that define the spatial scope of a regulator’s jurisdictional authority (cf. Cattani et al., 2017) and, thus, who or what is inside versus outside a claimed territory (cf. Sack, 1983).

Yet, since this regulator-centered conception of territory does not account for how a regulator’s territory claims are interpreted and responded to by their targets (e.g., the organizations subject to the regulator’s claims), we inductively developed a novel, organization-centered conceptualization of regulatory territory. Our conceptualization emphasizes the role of unprosecuted organizations’*territorial self-categorization*, a form of spatial self-categorization (cf. Huttenlocher et al., 1991) that leads organizations to conclude whether they are located inside what they perceive to be a regulator’s territory. Territorial self-categorization is key for explaining cross-border general deterrence: Unprosecuted banks perceive a high prosecution threat and decide to comply with U.S. law only if they categorize their organizations as located inside U.S. regulatory territory.

The cultural–cognitive perspective on territorial self-categorization we developed suggests that the categorizations made by different groups of unprosecuted organizations are shaped by their taken-for-granted categorization schemes (cf. DiMaggio, 1997; Glynn & Navis, 2013). Following a distinction introduced to cultural sociology by Robert Merton (1957) to capture differences in actors’ receptiveness to outside influences, we refer to the two groups in our study as *cosmopolitans* and *locals* (cf. Beck, 2006; Levy et al., 2016). We show and theorize how and why these groups responded differently to the foreign regulators’ enforcement events as a result of their different territorial categorization schemes and of the different levels of clarity with which the foreign regulators communicated their territorial category in the two events.

## Research Setting

### The Swiss Offshore Private Wealth Management Industry

Our organizational population consisted of 93 banks with a Swiss-licensed PWM entity that engaged in an offshore PWM business with U.S. clients.^2^ Here, *U.S. clients* are individuals who face tax liabilities in the U.S. and hold bank accounts in Switzerland. *Offshore PWM* is the provision of financial services to private clients through entities located and licensed outside a client’s country of residence. The offshore business serves international markets through a licensed entity in the so-called home country and without a foreign-licensed subsidiary.

The offshore business generally builds on jurisdictional arbitrage by exploiting legal differences across jurisdictions (e.g., Carruthers & Lamoreaux, 2016; Cerny, 1994; Fleischer, 2010; Riles, 2014; Tarko & Farrant, 2019). Like other secrecy jurisdictions (OECD, 2000), Switzerland had rules that were favorable to the offshore PWM business. Swiss law conceived only of tax fraud (i.e., active deception, such as the falsification of documents), not of tax evasion (i.e., the nonreporting of assets), as a criminal charge (Felber, 2009). Thus, Swiss banks’ facilitation of U.S. clients’ tax evasion (Permanent Subcommittee on Investigations, 2006) was not considered a criminal offense and was tolerated under Swiss law. Swiss regulators, i.e., the Swiss Financial Market Supervisory Authority (FINMA) and Swiss courts, emphasized their sovereignty and generally denied competing jurisdictional claims (Gully-Hart & Carron, 2012; Lembo & Hari, 2012). Banks were prohibited by the Swiss banking secrecy law to disclose information about their clients (Bundesgesetz über die Banken und Sparkassen, 1934) except if the clients themselves agreed that the banking secrecy be waived.

With their offshore PWM business, Swiss banks facilitated U.S. clients’ tax evasion. They accepted, hosted, and often managed undeclared assets of U.S. clients by offering offshore bank accounts. Some also offered structured products and related services—such as depots, coded accounts, shell structures, insurance wrappers, or hold mail agreements^3^—that further concealed the undeclared assets from U.S. regulators’ scrutiny (DOJ, 2015, 2018; Permanent Subcommittee on Investigations, 2006). In 2006, experts estimated that U.S. citizens evaded between USD 40 billion and 70 billion in U.S. taxes per year (Permanent Subcommittee on Investigations, 2006). The Swiss offshore PWM industry flourished: In 2010, undeclared accounts were cautiously estimated to amount to CHF 200–300 billion (Schöchli, 2010), a 27 percent share of the global cross-border private banking market in 2007, with approximately 70 percent of the premium volume originating from abroad (Zulauf, 2010). Because Swiss banks operated through their Swiss-licensed PWM entity, they enabled U.S. clients to book assets under Swiss laws and supposedly out of foreign regulators’ reach (DuPasquier and Fischer, 2010).

### The U.S. Regulator: The Conspiracy Statute, the Protective Principle of Jurisdiction, and Goal-Based Territorial Categorization

Most Western regulators follow the territoriality principle (cf. Meyer et al., 1997): a foundational principle in international law, enshrined in the constitutions and legal codes of most Western democracies (Ryngaert, 2015), that defines regulatory territory geographically. For the Swiss regulator, this principle appears in Article 3 of the 1937 Swiss Criminal Code. The U.S., however, regards itself as less restricted by public international law than most Western countries do (Ryngaert, 2015). U.S. regulators are authorized to engage in extraterritorial law enforcement, e.g., to protect U.S. interests (Dodge, 2020; Kirmayer, 1988; Ryngaert, 2015; Verdier, 2019). In the foundational case *United States v. Bowman* (1922), the U.S. Supreme Court held that U.S. law should be enforced extraterritorially when U.S. governmental agencies are the victims, for instance, in “conspiracies to defraud the United States.” The conspiracy statute (18 U.S.C. §371) creates an offense if “two or more persons” conspire either to “commit any offense against the United States, or to defraud the United States, or any agency thereof in any manner or for any purpose” (BBC, 2017).^4^ Drawing on the protective principle of jurisdiction, the Court stated that the statute seeks to protect the U.S. government from crime, wherever perpetrated and irrespective of particular geographical attributes (Dodge, 2020; Kirmayer, 1988; Ryngaert, 2015).

The protective principle provides U.S. regulators with what we refer to as a *goal-based territorial category* (cf. Durand & Boulogne, 2017; Durand & Paolella, 2013). Unlike attribute-based categories, which associate members with the category based on category members’ shared attributes, goal-based categories associate members with the category based on the categorizer’s goal even if category members differ greatly in their attributes (Boghossian & David, 2021; Durand & Boulogne, 2017). Accordingly, whether actors are inside or outside a state’s goal-based territorial category does not depend on actors or their actions exhibiting certain geographic attributes; it depends on whether their prosecution advances the state’s regulatory goal (i.e., to protect the state from harm, in this case, from conspiracies against it), no matter where the violations originate (Fichtelberg, 2014; Ryngaert, 2015).

### U.S. Extraterritorial Law Enforcement Against Swiss Banks and Variations in General Deterrence in Switzerland’s PWM Industry

In contrast to Swiss law, the “aiding and abetting” of tax evasion constitutes a criminal offense in the U.S. Code, namely a “conspiracy to defraud the United States” in tax matters (DOJ, 2009: 1), which can be prosecuted extraterritorially. However, the U.S. regulators—the Department of Justice (DOJ) and the Internal Revenue Service (IRS)—did not extraterritorially enforce this national law against foreign banks until 2008. In that year, the DOJ’s first extraterritorial enforcement event targeted UBS, Switzerland’s largest bank and a global PWM leader, for having committed a conspiracy to defraud the U.S. in tax matters. In 2011, a second extraterritorial enforcement event again relied on the conspiracy statute and targeted Switzerland’s oldest bank, Wegelin & Co.

To enforce national law extraterritorially, states require high coercive power. They typically impose compliance on foreign private actors via the threat to otherwise cap their access to a critical resource (e.g., Kaczmarek & Newman, 2011; Kalyanpur & Newman, 2018). The U.S. regulators had sufficient coercive power to impose compliance with their laws on the prosecuted banks (Kalyanpur & Newman, 2018): They could threaten to otherwise cap banks’ access to the U.S. dollar system, which they depend on for interbank transactions.

**Figure 1.**
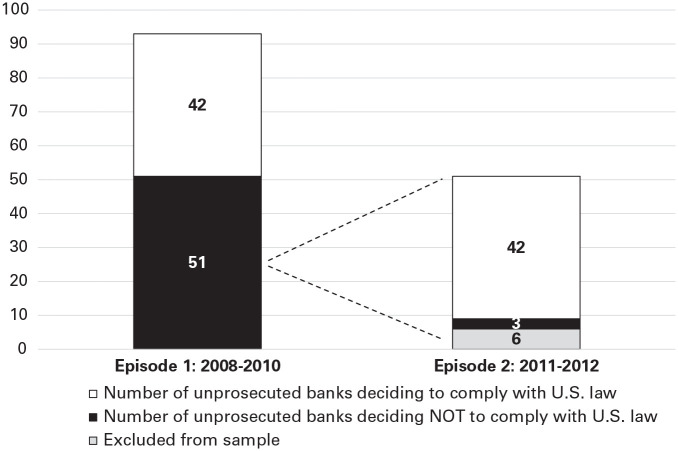
Overview of Banks’ Decisions to Stop Violating U.S. Law per Episode

To understand and explain these two U.S. extraterritorial law enforcement events’ general deterrence effects on Switzerland’s offshore PWM industry, we focused on a sample of 93 Swiss banks. Before 2008, none of the sampled Swiss banks complied with U.S. law. The deterrence message that U.S. regulators intended to send to the entire Swiss PWM industry by conducting the extraterritorial prosecution of UBS in 2008 was partially received (Bharara, 2013): Of the 93 banks, 42 decided to comply with U.S. law as a result of the 2008 prosecution, and 51 did not. In contrast, the 2011 prosecution of Wegelin had a more substantial general deterrence effect. Of the 51 noncompliant banks, we excluded from our subsequent analysis Wegelin plus five other banks.^5^ Of the 45 remaining noncompliant banks in our sample, 42 became compliant with U.S. law during our observation window, and three banks became compliant shortly after. Figure 1 shows an overview of banks’ decisions after each event. Below, we explain and theorize the variations in these extraterritorial law enforcement events’ general deterrence effects.

## Research Methods

### Data

Our primary data source (see Table 1) was archival. Because all 93 banks in our sample settled with the DOJ through nonprosecution or deferred prosecution agreements after our observation period, we could access the DOJ’s detailed statements of facts (SOFs) for every bank (DOJ, 2018), hearings, and investigation reports. We also used some banks’ board minutes and available annual reports, as well as 355 public, real-time, direct or indirect statements from bankers and regulators that appeared in newspaper articles. To grasp the interpretations of the people affected by these two prosecution events (Manning, 1986), we participated in industry events and conducted 103 semi-structured interviews with executives and legal professionals from 67 banks, Swiss regulators, and other experts (i.e., representatives of the banks’ legal consulting firms and associations, analysts); see Table 2 for an overview of our key informants. Given the topic’s sensitivity, we could not interview every bank. We sampled interviewees for their unique insights and offered them strict confidentiality agreements, guaranteeing that no statement would trace back to any individual or organization, to ensure the statements’ veracity (Miles & Huberman, 1994). Our interviews increasingly focused on cultural–cognitive factors and territorial categorization processes. To account for potential alternative explanations, we crafted quantitative measures based on data from sources such as *TheBanks.eu* and *moneyhouse.ch* databases, the *Bilanz* ranking of Swiss private banks, and the DOJ’s statement of facts.

**Table 1.** The Data

Data Sources	Amount	Use in Analysis
**Archival data**	**Approx. 7,035**	
Statements of facts by the DOJ for the banks that concluded nonprosecution or deferred prosecution agreements	1,291 pages	Uncover the banks’ illegal behaviors, underlying rationales, and the time of their decisions to comply with U.S. law, as well as some bank information, e.g., assets under management, headquarters location, international presence, legal status, markets, U.S. assets/accounts
U.S. Senate’s Permanent Subcommittee on Investigations (PSI)	2,872 pages	Uncover the nature of the U.S. regulators’ law enforcement acts; detailed descriptions of some prosecuted banks’ illegal behaviors; and in-depth insights into some executives’ interpretations and justifications
IRS and DOJ accusation documents, reports, and media releases	245 pages	
Reports by Swiss banking industry consultants and experts	872 pages	Triangulate the banks’ actions
Annual reports, media releases, and websites of some banks	1,200 pages	Triangulate the bankers’ interpretations of the events, and some bank information
Swiss authorities’ documents and reports	555 pages	Uncover the Swiss regulator’s interpretations and decisions
*TheBanks.eu* database		Information on banks’ age, AUM, market share, ownership
The *Bilanz* rating		Information on the firms’ prominence
The *moneyhouse.ch* database		Triangulate bank information
**Additional archival media data**		
Real-time interviews and interview statements (extracted from media data mentioned below)	355 interviews & statements	Uncover Swiss bankers’ and the Swiss regulator’s real-time interpretations of events and key decisions
Swiss and international newspaper and outlet articles from: Blick (B); Financial Times (FT); Finanz&Wirtschaft (FW); Handelszeitung (HZ); Neue Zürcher Zeitung (NZZ); Reuters (R); Swissinfo (online news portal); Tagesanzeiger (TA); Weltwoche (WW)	Approx. 7,000 pages	Extracted the abovementioned 355 public interviews and interview statements with key actors; timeline of key events; initial immersion in the field
Television data (Swiss National Broadcasting Agency, SRF), documentaries, and live debates	Approx. 27 hours	Triangulate the bankers’ interpretations of events
**Retrospective interviews** with key representatives of Swiss banks, Swiss regulators, and leading experts on the issue	103 interviews (average length: approx. 60 min)	Triangulate and validate the theoretical model in 28 interviews and fine-tune the underlying mechanisms; triangulate and validate the derived interpretations and decisions; initial immersion in the field
**Field-level immersion** at Swiss private banking events (2012 to 2014)	Approx. 160 hours	Triangulate the bankers’ interpretations of events

**Table 2.** Key Informants

Informant	Retrospective (Own) Interviews	Real-Time Interview Statements (Extracted from the Newspaper and Outlet Data)
Swiss bank executives		
CEO	19	140
TMT member/legal councils	45	28
Chairman/board member/partner	13	40
Legal/industry experts	24	59
Swiss authorities	2	88
**Total**	**103**	**355**

### Data Analysis

Our purpose was to open the black box between the extraterritorial law enforcement events and unprosecuted banks’ (non)compliance and to unpack the process, specifically the sequence of variables and mechanisms that connects these two phenomena (e.g., Bennett & Checkel, 2014; Knight & Reed, 2019). We used comparative case study (Skocpol & Somers, 1980) and inductive theory-building methods (Corley & Gioia, 2004; Locke, 2001).

First, drawing on extensive real-time data, we created a thick description of key events, actors, and decisions. This served as an “organization device” to reduce and order our extensive raw data, while retaining a “high degree of authenticity” (Langley, 1999: 695). Because the enforcement acts against UBS and Wegelin were turning points for Swiss banks’ decisions, we treated them as events (e.g., Trevino, 1992; Yiu et al., 2014). We bracketed the period after the key events (see Langley, 1999; Skocpol & Somers, 1980) as follows: Episode 1: the UBS case (2008 to 2010) and Episode 2: the Wegelin case (2011 to the end of 2012).

Using the statement of facts, we measured the banks’ decision to (not) comply with U.S. law as the year in which the bank’s management or board decided to (not) stop violating U.S. law by requiring signed IRS W-9 or W-8 forms from all existing and new U.S. clients.^6^ The bankers knew that complying would reveal their business with U.S. clients’ undeclared assets to the IRS and that they would need to stop facilitating U.S. clients’ tax evasion in their PWM business and to settle with the U.S. regulators on past violations of U.S. law. We used our archival data from the DOJ to further validate that the banks subsequently enacted their compliance decision. We validated the key events, actors, and differences in decision patterns in the interviews. We found that banks varied greatly regarding whether they complied in Episode 1 or in Episode 2.

**Figure 2.**
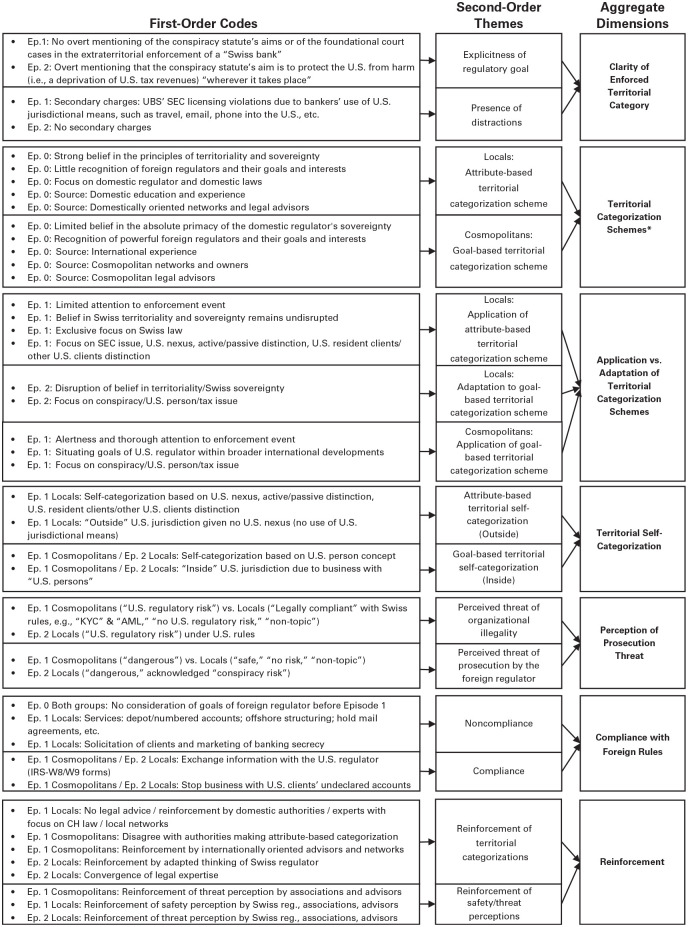
Data Structure* * Not part of theoretical model (see Figure 3). Informs Tables 4 and 5.

Second, this first finding triggered within-case analyses (Yin, 2009) of every bank so that we could derive the factors and rationales that led to their decision to (not) comply. Initially, we focused on a subset of cases in which these factors and rationales were “transparently observable” (Eisenhardt, 1989: 537). We identified the bankers’ interpretations of the U.S. regulators’ jurisdictional reach as the key factor to explain these decisions. Here, the literature on territory became central. To develop a systematic understanding of these interpretations, we coded our data thematically (Strauss & Corbin, 1994); see Figure 2. We started coding fragments of data (Locke, 2001), resulting in a total of approximately 990 codes, which evolved through constant comparison into more-abstract themes (Strauss & Corbin, 1994). By iterating between themes and relevant literatures, we made theoretical sense of why themes emerged. For instance, codes such as “a strong belief in the territoriality of Swiss law” and “Swiss law applies on Swiss soil” were clustered under the axial theme “strong belief in the territoriality principle.” These contrasted with codes such as “we have to deal with realpolitik” and “there were already precedents where the Americans had done this before,” which we clustered under the theme “recognition of powerful regulators’ territorial goals.” This led us to inductively derive what we refer to as *territorial categorization schemes* as the key interpretive mechanism that led to banks’ (non)compliance decisions. These schemes foreground a particular point of emphasis—in our case, either the territoriality principle or regulators’ territorial goals—that makes a certain set of interpretations salient (cf. Entman, 1993): using either geographic attributes or regulators’ goals as evaluative features to circumscribe regulatory territory’s scope. To conceptualize them into attribute-based vs. goal-based territorial categorization schemes, we drew on the cultural–cognitive perspective on categorization (Durand et al., 2017; Grodal, 2018; Jepperson & Meyer, 2021).

Third, to derive a generalizable theory of general deterrence, we sought to replicate the emerging concepts and the relationships across all banks to spotlight similarities and differences in interpretations and decisions (Leonard-Barton, 1990: 255; Yin, 2009: 226), as well as patterns across time (Skocpol & Somers, 1980). We identified patterns in interpretive data that suggest the role of cultural categorization schemes for explaining differences in the compliance decisions across two groups of banks, as well as evidence for change in these schemes over time for one group. Our evidence suggests that these schemes were collectively constituted and were shared mental representations among executives and legal professionals within a given bank and across the banks in a group. As we probed why banks held one of these two schemes, we discovered that they had different orientations. Following insights in cultural sociology by Merton (1957) (cf. Beck, 2006; Hannerz, 1990; Levy et al., 2016; Robertson & Wind, 1983), we refer to banks that are more open to outside influences as *cosmopolitans*, while *locals* are more inward-oriented. We derived these cultural groups in three steps. First, using our various data sources, we inductively developed a taxonomy for the cultural groups and then refined and validated it with experts. Second, we compiled evidence on each bank for each dimension of the taxonomy and coded each bank into one of the cultural groups, with either a cosmopolitan (46 banks) or a local orientation (47 banks). Third, we validated these codings with experts by using a card-sorting procedure. We asked the experts to independently place each bank into either a more cosmopolitan or local group based on the taxonomy (Neuendorf, 2002). We then discussed the similarities and differences between the experts’ and our own categorizations of the banks until we reached agreement. We found that these cultural orientations largely explained the differences in the timing of the banks’ compliance decision: The cosmopolitans complied after the UBS event (with four exceptions), and the locals complied after the Wegelin event (with three exceptions).

The concepts are represented in a code structure (see Figure 2). Exemplary evidence is listed in Table 3 and in Online Appendix I. We further validated our inductively derived model in interviews (Vuori & Huy, 2016); see Table 2. Finally, to account for possible alternative explanations based on established literatures, we assessed whether there were important differences between the two enforcement events and between the two groups of banks (Online Appendix II).

**Table 3.** Exemplary Evidence for the Codes*

Episodes & Actors	Codes & Quotes	Quote No.
	Foreign Regulator: Clarity of Enforced Territorial Category	
**Episode 1**	**Explicitness of regulatory goal**	
	“. . . charging UBS with participating in a conspiracy to defraud the United States and its agency the Internal Revenue Service (‘IRS’) in violation of 18 U.S.C. § 371.” (United States District Court Southern District of Florida, CASE NO. 09-60033-CR-COHN, DPA: 1)	OA1
	“UBS . . . participated in a scheme to defraud the United States and its agency, the IRS, by actively assisting or otherwise facilitating a number of United States individual taxpayers in establishing accounts at UBS in a manner designed to conceal the United States taxpayers’ ownership or beneficial interest in these accounts.” (United States District Court Southern District of Florida, CASE NO. 09-60033-CR-COHN, DPA: 1)	OA2
	**Presence of distractions**	
	“These client advisers [of UBS] travelled to the U.S. . . . In many instances, the client advisers attended exclusive events such as art shows, yachting events, and sporting events that were often sponsored by UBS, for the purpose of soliciting and communicating with United States cross-border clients. UBS also used other U.S. jurisdictional means such as telephones, facsimiles, mail and e-mail to provide securities services to its U.S. cross-border clients.” (U.S. Securities and Exchange Commission, Litigation Release No. 20905 / February 18, 2009)	OA7
	“UBS took action to conceal its use of U.S. jurisdictional means to provide securities services. Among other things, client advisers typically travelled to the U.S. with encrypted laptop computers that they used to provide account-related information, to show marketing materials for securities products . . . As charged in the SEC’s complaint, as a result of its conduct, UBS violated Section 15(a) of the Securities Exchange Act of 1934 and Section 203(a) of the Investment Advisers Act of 1940.” (U.S. Securities and Exchange Commission, Litigation Release No. 20905 / February 18, 2009)	OA8
**Episode 2**	**Explicitness of regulatory goal**	
	With the Wegelin case, U.S. authorities intended to “send a message of deterrence to banks . . . who would believe that, without a physical presence in the United States, they cannot be reached by U.S. law enforcement. . . . the lack of physical presence will never be an impediment to U.S. law enforcement’s acting to protect the IRS’ ability to collect revenue from U.S. taxpayers.” (DOJ130225, 2013)	OA15
	“. . . the prosecution of Wegelin is well within the bounds of the extraterritorial application of federal criminal law. Our Constitution permits, consistent with constitutional due process, the extraterritorial application of federal criminal law to non-citizens acting entirely abroad ‘when the aim of that activity is to cause harm within the United States or to U.S. citizens or interests.’” United States v. Al Kassar, 660 F.3d 108, 118 (2d Cir. 2011); see also United States v. Mardirossian, 818 F. Supp. 2d 775, 776 (S.D.N.Y. 2011) (noting that presumption against extraterritorial application of criminal statutes does not apply to statutes that are ‘not logically dependent on their locality for the government’s jurisdiction, but are enacted because of the right of the government to defend itself against obstruction, or fraud wherever perpetrated’) (citing United States v. Bowman, 260 U.S. 94, 98 (1922).” (Rakoff in DOJ, 20130225: 14)	OA16
	**Presence of distractions**	
	n.a.	
	Unprosecuted Organizations: Territorial Categorization Schemes	
**Cosmopolitans**	**Goal-based territorial categorization scheme**	
	*Emphasis of territorial categorization scheme: Foreign regulator’s interests and goals*	
	Bank8X: “One must not only look at these things in a purely legal and principled way, of course, but above all also in a factual way . . . The power of the factual.” (Int. 56)	OA30
	Bank5X: “A greater openness to and also a greater respect of the legal situation in the U.S.” (Int. 89)	OA34
	*Sources of territorial categorization schemes*	
	“They had a different type of socialization, a different type of openness to international issues and better international sensitivity. They reacted faster and more strongly to this extraterritoriality.” (Int. 82)	OA57
	“Networks play a role, and so do associations. Banks are herd animals. And then they talk about it in the Bankers Association. That has enormous power. This Group Think is enormously prevalent.” (Int. 83)	OA94
	Bank7X: “We had contacts. You could exchange ideas. We had a connection with lawyers in Washington, New York.” (Int. 92)	OA115
**Locals**	**Attribute-based territorial categorization scheme**	
	*Emphasis of territorial categorization scheme: The principles of territoriality and sovereignty*	
	Bank9X: “A strong belief in the territoriality of Swiss law, so that basic idea that Swiss law applies on Swiss territory, on Swiss grounds. No other regulator can just come and displace it.” (Int. 87)	OA120
	“The affinity to international compliance topics in general was not developed at all.” (Int. 83)	OA136
	*Sources of territorial categorization schemes*	
	“Trust in Swiss law taught at Swiss universities.” (Int. 64)	OA161
	Managers with a “fairly national orientation. They have rarely spent longer periods abroad and possess no international education. . . . they also tend to accumulate mandates in the Swiss political field.” (Araujo, 2020: 107)	OA168
	“The association of [domestic banks] and its sub-associations in which members convened and exchanged information regularly.” (Int. 54)	OA174
	Bank0X: “Our compliance consultant did not deal with the intricacies of U.S. law. Rather, he focused on Swiss law. What works and what doesn’t work in Swiss law.” (Int. 62)	OA175
	Unprosecuted Organizations: Application vs. Adaptation of Territorial Categorization Schemes	
**Episode 1**		
**Cosmopolitans**	**Application of goal-based territorial categorization scheme**	
	Bank4X: “There was a different mood that was perceived, it was not conceivable that you would violate laws of other countries, so overall this orientation or this interpretation, this greater perception of these international dynamics, seems to have been more present here than perhaps in other banks.” (Int. 66)	OA187
	Bank3X: “It is about tax law and that of course starts with U.S. person and not the residence.” (Int. 59)	OA192
	Bank7X: “The whole UBS affair is not an SEC affair at all. . . . It’s really a tax case.” (Int. 92)	OA196
**Locals**	**Non-adaptation to goal-based territorial categorization scheme**	
	Bank1X interpreted that “[t]he exceptional case of UBS” was due to the UBS’ business in the U.S. Similar regulatory issues would not be faced by banks that would focus on “Switzerland” only. (SOF14, 2015)	OA257
	Bank1X: “One looked primarily at these licensing aspects and at this residency status.” (Int. 84)	OA258
**Episode 2**		
**Locals**	**Adaptation to goal-based territorial categorization scheme**	
	“After the Wegelin case, Swiss banks realized: Stop. The U.S. regulator can very well take action, even extraterritorially.” (Int. 65)	OA286
	Bank79: Wegelin was “indicted in the U.S. and charged with conspiracy to defraud the U.S. by assisting U.S. taxpayers in opening and maintaining undeclared accounts in Switzerland.” (SOF79, 2015)	OA298
	Unprosecuted Organizations: Territorial Self-Categorization	
**Episode 1**		
**Cosmopolitans**	**Goal-based territorial self-categorization (Inside perceived territorial category of foreign regulator)**	
	[Bank22] needed “to ensure that U.S. customers complied with their U.S. tax obligations.” (SOF22, 2015)	OA313
	Bank9X: “[W]e have the risk of aiding and abetting if we offer our very normal banking services without checking or clarifying whether a customer is taxed.” (Int. 73)	OA339
**Locals**	**Attribute-based territorial self-categorization (Outside perceived territorial category of foreign regulator)**	
	Bank0X: “management was aware that U.S. authorities were pursuing Swiss banks that facilitated tax evasion for U.S. accountholders in Switzerland. But [Bank0X’s] management was not deterred by this action because the Bank had no U.S. presence.” (SOF2, 2015)	OA344
	Bank2X considered itself “as a local bank under Swiss law [because] we do not give any advice abroad, neither by e-mail nor by phone.” (SOF26, 2015)	OA361
**Episode 2**		
**Locals**	**Goal-based territorial self-categorization (Inside perceived territorial category of foreign regulator)**	
	Bank1: “U.S. taxpayers without a Form W-9” were seen as a gateway for U.S. exposure. (SOF1, 2015)	OA431
	Bank21 felt that banks would be subject to “U.S. law” with their “U.S. person” business. (SOF21, 2015)	OA438
	Unprosecuted Organizations: Perception of Prosecution Threat	
**Episode 1**		
**Cosmopolitans**	**Perceived threat of organizational illegality**	
	Bank4: “In mid to late 2008, in the wake of the UBS investigation, [Bank4] began to assess the risks of its own U.S. cross-border business. . . . [Bank4] sought to avoid . . . risks that could arise from the unauthorized solicitation of U.S. clients” in relation to U.S. law. (SOF4, 2015)	OA458
	Bank5: “this conduct violated U.S. law.” (SOF5, 2015)	OA459
	**Perceived threat of prosecution by the foreign regulator**	
	Bank5X: “After the UBS case . . . we thought: This story is only just beginning. In any case, they’ll be looking at who’s doing what in the financial center as a whole.” (Int. 68)	OA486
	“[W]e can be prosecuted by . . . foreign jurisprudence, as accomplices of tax evasion. At the moment, I am having this point investigated within the bank.” (Interviewee; Weltwoche12/03/09)	OA493
**Locals**	**Perceived threat of organizational illegality**	
	Bank13: “. . . perceived low level of risk exposure for the Bank.” (SOF13, 2015)	OA504
	“On the surface, we have no need for action due to the legal situation. . . . I feel confident. The legalistic position is well-anchored. . . . A simple tax evasion is no reason not to accept money due to the double litigation principle coupled with banking secrecy.” (Interviewee; DieWeltwoche 20/03/08)	OA525
	**Perceived threat of prosecution by the foreign regulator**	
	Bank14: “Bank Executive #I also stated that ‘there is practically no risk if U.S. customers travel to Switzerland and a customer account is handled locally.’” (SOF14, 2015)	OA536
	Bank38: “assessed the risk . . . to fall under U.S. bank supervision as low.” (SOF38, 2015)	OA546
**Episode 2**		
**Locals**	**Perceived threat of organizational illegality**	
	Bank19 “modified its policies and procedures to gradually ensure that it no longer assisted undeclared U.S. taxpayers in evading U.S. income tax.” (SOF19, 2015)	OA587
	Bank68 “instituted policies that were intended to limit its potential criminal and civil tax liability by ensuring compliance with U.S. laws.” (SOF68, 2015)	OA601
	**Perceived threat of prosecution by the foreign regulator**	
	Bank14 Board Member #I: “There is the latent risk that previous revenues from this U.S. strategy of [Bank14] are seized or that corresponding fines are imposed on the bank. Based on the current developments in the industry, the probability of occurrence has increased.” (SOF14, 2015)	OA609
	Bank1X: “the potential risk of not even being convicted in the U.S., but being hauled into an American court, having to defend yourself there, and imagining the consequences that that could have.” (Int. 91)	OA611
	Unprosecuted Organizations: Compliance with Foreign Rules	
**Before Episode 1**	**Noncompliance**	
	Bank5X: “didn’t really care about that . . . it was up to the customer what he wanted to do.” (Int. 78)	OA626
	Bank9X: “The general attitude was, it’s not our issue. You’re responsible for your taxes.” (Int. 87)	OA628
**Episode 1**		
**Cosmopolitans**	**Compliance**	
	In December 2008, Bank39 ensured clients’“compliance with their U.S. tax obligations, (ii) waive Swiss banking secrecy, and (iii) provide a Form W9.” (SOF39, 2015)	OA662
	Bank50: “On November 20, 2008, Bank50 decided not to conduct business with U.S. clients unless these persons were willing to disclose their assets . . . to U.S. tax authorities. Later in 2009, Bank50 also started to require U.S. clients . . . to provide W-9 forms.” (SOF50)	OA665
**Locals**	**Noncompliance**	
	“Wegelin was undeterred by the investigation and prosecution of UBS . . . kept assisting U.S. taxpayers in evading taxes and did so for a significant period of time.” (DOJ130225, 2013: 13)	OA737
	“UBS then inevitably threw out its undeclared US clients. And there were, unfortunately, banks that picked up the nuclear waste that UBS had thus deposited.” (Int. 69)	OA780
**Episode 2**		
**Locals**	**Compliance**	
	Bank21: “In February 2012, the Bank’s management [required] . . . all existing U.S. clients to submit to the Bank a Form W-9. Accounts of clients who failed to provide the form were closed.” (SOF21, 2015)	OA863
	Bank75 “started its exit program in 2011. . . . began requiring . . . a Form W-9.” (SOF75, 2015)	OA879
	Epistemic Authorities: Reinforcement	
**Episode 1**		
**Cosmopolitans**	**Reinforcement of territorial categorizations**	
	“FINMA . . . didn’t warn, we didn’t understand that, and we still don’t understand that today.” (Int. 68)	OA891
	“The [association of international banks] has held many events. U.S. law firms also appeared.” (Int. 69)	OA894
	**Reinforcement of threat perceptions**	
	“Bankers went to the meetings of the [bank association]. We have been advised that this is an issue. Or bankers . . . had sensitivities with the US . . . because of their experience.” (Int. 82)	OA923
	Bank57: “The [foreign] Group management had a lot of respect for these risks. . . . has always reacted rather critically to Swiss management and always had an uneasy feeling somewhere. . . . this has a very strong influence on how you perceive risks.” (Int. 65)	OA925
**Locals**	**Reinforcement of territorial categorizations**	
	Swiss regulators highlighted: “A problem arises, if client advisors of Swiss banks travel on-site, hence to the clients abroad, to render such services. They then have to obviously fulfil the legal prescriptions of the target country.” (GPK, 20100530: 3392)	OA938
	Bank0X: “Our compliance consultant said, no, that wasn’t a problem, because he looked after many banks, where he also continued to drive exactly this track.” (Int. 62)	OA943
	Bank78 “executives did not believe outside legal advice was necessary. In their view, the Bank could open accounts for U.S. taxpayers to give them a place to park their undeclared assets.” (SOF78, 2015)	OA945
	**Reinforcement of safety perceptions**	
	“The Federal Council at the time said that banking secrecy would never be betrayed, over my dead body. And that already gave the feeling of security.” (Int. 64)	OA959
	“They were in the same soup, in the same network, and exchanged information and kind of reassured themselves that nothing can happen. That they can just close the fences.” (Int. 82)	OA960
**Episode 2**		
**Locals**	**Reinforcement of territorial categorizations**	
	“From a criminal perspective, banks and their employees can make themselves liable to prosecution for facilitating tax offenses. This can even apply, if they are only active in Switzerland.” (Interviewee, FINMA, Finanz&Wirtschaft 24/03/10)	OA965
**Epistemic Authorities: Reinforcement**		
	“From a foreign law perspective, it is very easy to challenge Swiss banking secrecy, in that one says, the conduct is conspiracy, it is indictable.” (Int. 8)	OA966
	**Reinforcement of threat perceptions**	
	FINMA “calls on [Swiss] institutions to also comply with foreign supervisory law and define an appropriate service model for each target market.” (FINMA, 22/10/11)	OA986

* The OA number refers to the number of the quote in Online Appendix I.

## The Case Study

We rely on our first-order codes (see Figure 2) to present our findings. Below, we first present the two inductively derived groups of banks (see Table 4) and then detail how they responded to the U.S. extraterritorial law enforcement events (Episode 1 and Episode 2). We share the cited empirical references with the abbreviation OA in the Online Appendix.

**Table 4.** Two Groups of Banks

	Local Banks (47)	Cosmopolitan Banks (46)
Bank types*	1. Local Swiss-owned inland banks (20) 2. Local Swiss-owned pure players orhybrids (19) 3. Local pure players or hybrids withforeign owners from other secrecyjurisdictions (8)	1. Cosmopolitan Swiss-owned inland banks (6) 2. Cosmopolitan Swiss-owned pure players or hybrids (6) 3. Internationally oriented Swiss-owned hybrids oruniversals (7) 4. Banks owned by foreign multinational banks (23) orforeign corporations (4)
International presence	Overall limited Some pure players or hybrids have a presence in other secrecy jurisdictions Banks with U.S. presence: 0	Overall limited (but slightly higher than local banks) Except for the internationally oriented Swiss-owned banks, which are more internationalized Banks with U.S. presence: 3 (of which only 1 PWM-related)
Compliance departments	Predominant focus on Swiss law Rather small and potentially outsourced to a consultancy	More focused on cross-border regulatory issues Larger and better resourced
Executives and compliance professionals	Domestic education, domestic industry experience, experience with domestic regulatory issues	International education, international industry experience, experience with cross-border regulatory issues
Epistemic authorities	Domestic regulator as ultimate epistemic authority Domestically oriented legal advisors Locally oriented associations, other locally oriented banks, and other actors	More emancipated relationship to the domestic regulator as epistemic authority (especially if in conflict with other regulators’ claims) Internationally oriented legal advisors Cosmopolitan associations, other cosmopolitan banks, and other actors

* **Local bank types:** (1) Swiss inland banks are universal banks (see footnote 2 for definitions of pure, hybrid, and universal banks). They focus on the national, cantonal, or regional communities where they are based, without any international presence. Most are owned either by the state, the canton, or the local community. (2) These are traditional, Swiss-owned pure players or hybrids with little to no international presence. (3) These pure players and hybrids are subsidiaries of foreign owners (typically from other secrecy jurisdictions). Some have an international presence (typically in other secrecy jurisdictions).

**Cosmopolitan bank types:** (1) and (2) are Swiss inland banks, pure players, or hybrids with little to no international presence that had either experienced prior cross-border regulatory issues and/or had more internationally oriented executives, and/or had strong ties to other cosmopolitan banks. (3) The internationally oriented Swiss banks were Swiss-based yet globally active hybrids or universal banks with an extended, mostly non-U.S. international presence. (4) These are separate, fairly small Swiss-licensed legal entities (pure players, hybrids, or universal banks) of multinational banks headquartered abroad that had a strong influence.

### The Local and the Cosmopolitan Banks and Their Territorial Categorization Schemes

The two groups of banks differed in their taken-for-granted schemes for categorizing the jurisdictional reach of national regulators, such as the U.S. regulator (see Table 5). Both groups held these schemes before the first U.S. extraterritorial law enforcement event.

**Table 5.** Two Territorial Categorization Schemes

	Attribute-Based Territorial Categorization Scheme (Local Banks)	Goal-Based Territorial Categorization Scheme (Cosmopolitan Banks)
Territorial categories and their evaluative features	Territorial attributes: Perceived geographic attributes in line with the territoriality principle	Territorial goals: Perceived goals of powerful foreign regulators as enacted in their (extraterritorial) enforcement events
Emphasis of territorial categorization scheme	Strong belief in territoriality and sovereignty as default principles in international law Focus on the domestic regulator and on the primacy of domestic laws Little recognition of powerful foreign regulators’ territorial goals	Limited belief in the absolute primacy of the domestic regulator’s sovereignty Recognition of powerful foreign regulators’ territorial goals
Underlying view of territorial authority and world order	Idealism: States are perceived as equal. They are seen as similarly obeying collectively agreed-upon norms and rules.	Realism: States are perceived as unequal. Powerful, and especially hegemonic, states are seen as following and enforcing their own territorial goals.

#### The local banks and their attribute-based territorial categorization schemes

The first group (47 banks) held what we refer to as an *attribute-based territorial categorization scheme* (cf. Durand & Paolella, 2013). They were united by a strong belief “in the territoriality of Swiss law, so that basic idea that Swiss law applies on Swiss territory, on Swiss grounds. And no other foreign regulator can just come and displace Swiss law” (OA120). Territoriality is a foundational principle of international law, and these bankers believed that national regulators would follow it (OA121, OA122, OA127). The bankers demarcated the scope of a national regulator’s jurisdictional authority geographically, believing that their banks would be inside a foreign regulator’s territory only if their legal violations had particular geographic attributes (i.e., if violations were committed within the foreign regulator’s geographic borders). If a bank was based (thus, licensed) in Switzerland—i.e., if its PWM entities had a “physical presence” (OA158), “ratione loci” (OA159), or “domicile” (OA160) in Switzerland—the Swiss regulator would be the sovereign or “lead” legal authority over their cross-border business (OA156).

Because their categorization scheme led them to focus on domestic regulatory issues, we refer to these banks as *locals* (cf. Levy et al., 2016; Merton, 1957). With their belief in the primacy of Swiss law (OA141), the locals had little recognition of foreign regulators’ goals and the legal challenges that could arise in cross-border business (OA135, OA145). They did not focus on the regulations of the clients’ country of origin but only on the Swiss ones (i.e., anti-money laundering, know-your-customer, etc.) (OA144). Their “insular way of thinking” (OA137) led them to “close the fences” (OA960), to “ignore other country laws . . . [and] think that you can run the business in a very local way in a homegrown jurisdiction with only homegrown actions” (OA40).

For the locals, the domestic regulator was their ultimate *epistemic authority*, i.e., source of jurisdictional expertise (Kruglanski, 1989; Zürn, 2018), whose interpretations strongly influenced their understanding of their regulatory environment. For legal advice, they predominantly contacted domestically oriented rather than internationally oriented legal advisors (OA987, OA988). Further, locals participated in Swiss-focused banking networks and associations, such as an association focused on banks with a domestic emphasis or one focused on private banking (OA173, OA174), in which the locals’ jurisdictional schema was largely shared and reinforced.

Independent of the banks’ size, the locals’ compliance departments were rather small and/or outsourced to local consultancies (OA176). The locals’ executives and compliance professionals had a distinct profile that constituted and/or reinforced their jurisdictional schema. They had predominantly studied in Switzerland and made their careers in domestic organizations (OA166, OA167) or in other secrecy jurisdictions that equally rejected foreign regulatory interference (OA163, OA170). For them, traditional Swiss values, the sovereignty of the nation-state, and the reliability of its laws and institutions had been foundational tenets (OA164).

#### The cosmopolitan banks and their goal-based territorial categorization schemes

In contrast, the second group (46 banks) held what we call a *goal-based territorial categorization scheme* (cf. Durand & Paolella, 2013) because they demarcated the spatial scope of a regulator’s jurisdictional authority based on the perceived jurisdictional goals of that regulator. Uniting this group was their limited belief in the absolute primacy of the territoriality principle and in the domestic regulator’s sovereignty (OA26). Following a less idealistic and more realistic view (Zuolo, 2012), the cosmopolitans did not believe that all national regulators adhered to the international principles, such as territoriality (OA27). Instead, they took for granted that powerful regulators can deviate from this principle to pursue their own goals (OA42, OA47).

Since their categorization scheme led these banks to be open to foreign influences (OA34, OA36), we refer to this group as *cosmopolitans* (cf. Beck, 2006; Levy et al., 2016; Merton, 1957). Before the extraterritorial enforcement against UBS, they had already recognized that some national regulators, especially in the U.S., had engaged in extraterritorial jurisdiction in other issue domains, for instance to fight corruption (e.g., Christensen et al., 2021; Parrish & Ryngaert, 2023), money laundering, and sanctions violation (e.g., Mallard & Sun, 2022), no matter where the conduct occurred (OA38). They paid attention to prior cases of extraterritorial jurisdiction targeted at actors based in Switzerland, e.g., Marc Rich, the founder of the commodity trader Glencore (e.g., Ammann, 2009). They were “more attentive to these developments in the U.S., more sensitive, more open, and also better able to absorb them” than the locals were (OA46).

Compared to the locals, the cosmopolitans had a more emancipated relationship with the domestic regulator as primary epistemic authority, particularly if that regulator’s interpretations contradicted other powerful regulators’ claims (OA885). The cosmopolitans were more connected to internationally oriented legal professionals (OA52, OA57, OA115, OA119). Their jurisdictional schemes were largely shared and reinforced in more internationally oriented networks, in which there was a greater openness to foreign regulatory goals. For instance, a network focused on private banking in Geneva considered international regulatory developments, with Geneva as the host of countless international organizations (OA96, OA107). An association focused on foreign banks regularly invited international legal experts (OA109). Also, the subsidiaries of foreign multinational banks and/or owners of some Swiss banks were embedded in cross-border networks (OA55, OA56, OA118).

Independent of the banks’ sizes, the cosmopolitans’ compliance function was more developed than that of locals. Their executives and compliance professionals had a distinct profile that constituted and/or reinforced their jurisdictional schema. These professionals had often been socialized and/or educated abroad and/or had international regulatory experiences either in Switzerland or in leading international banking centers (OA77, OA78). Compared to their local counterparts, they were less driven by local or national interests and embraced a more international field of action (OA81, OA91). Some self-classified as global citizens living in multiple countries and holding multiple passports (OA71, OA72).

#### Both groups’ noncompliance with U.S. law before Episode 1

Before the UBS event, both groups tended to be similarly inattentive to their U.S. clients’ tax status (OA632) and similarly in violation of U.S. laws, although for different reasons. Both the locals and the cosmopolitans believed that in light of Swiss law, the aiding and abetting of tax evasion was permissible (OA634, OA635). Owing to their belief in the territoriality of Swiss law, the locals categorically excluded the possibility that they could be prosecuted by a foreign regulator for their conduct in Switzerland. Although the cosmopolitans took the territoriality principle far less for granted and were more open to powerful foreign regulators’ goals, they also felt safe because foreign countries had never enforced their laws extraterritorially in this issue domain (i.e., conspiracy in tax evasion) before (OA633). Thus, they did not yet perceive a major prosecution risk from the U.S. (OA634). This changed after the UBS event.

### Episode 1: The Extraterritorial Law Enforcement Against UBS and Its Partial General Deterrence Effect in the Swiss Offshore PWM Industry (2008 to 2010)

#### The U.S. regulators

Following a whistleblower’s revelations (Birkenfeld, 2020), for the first time U.S. regulators mobilized the conspiracy statute to extraterritorially enforce U.S. law against a Swiss bank in July 2008 (Verdier, 2019, 2020). The DOJ and the IRS prosecuted UBS for its offshore private wealth management business’s alleged conspiracy in defrauding the U.S. government (OA1), i.e., for facilitating U.S. clients’ tax evasion in Switzerland (OA2). By unilaterally enforcing national law without Switzerland’s consent, the U.S. regulators deviated from the territoriality principle, undermined the Swiss regulator’s de facto sovereignty, and overruled the prior diplomatic path. They charged UBS for conspiracy because it created offshore structures for U.S. clients (e.g., shell companies) in Swiss UBS branches to hide U.S. clients’ untaxed assets from disclosure to the U.S. regulators (OA2).

After the prosecution of UBS in 2008, the U.S. regulators assumed that the Swiss offshore PWM industry should “have been deterred by the Department of Justice’s tax-related investigation of UBS and concluded that it should exit the business of assisting U.S. taxpayers in evading taxes” (OA6). However, in the enforcement event against UBS, the U.S. regulators did not clearly communicate the goal-based territorial category and extraterritorial reach of the U.S. conspiracy statute. They did not explicitly emphasize that the statute’s purpose was to protect the U.S. government and its agencies from harm no matter where such harm originated, for instance, by mentioning foundational court cases such as *United States v. Bowman* (1922). Thus, they did not clearly communicate that Swiss banks could be charged for committing a conspiracy against the U.S. no matter where geographically their conduct occurred. Further, the enforcement event distracted from the conspiracy statute’s extraterritorial reach in a second way. Although the primary charge levied against UBS was commission of a conspiracy against the U.S., UBS was *also* charged for having committed U.S. Securities and Exchange Commission (SEC) license violations (OA9). UBS’s bankers from Swiss-based PWM units had often traveled to the U.S. to solicit clients and “to market Swiss bank secrecy to United States clients interested in attempting to evade United States income taxes” (OA13). UBS was charged for using “U.S. jurisdictional means” without a U.S. banking license (OA7).

Following the enforcement, several UBS executives were indicted. Two, the head of the U.S. banking business and the global head of private banking, were arrested while abroad. They were extradited and jailed in the U.S. UBS stopped its non-U.S.-regulated PWM business with U.S. clients and, in a deferred prosecution agreement, paid USD 780 million in fines and restitution (OA4). Given that UBS was severely affected by the global financial crisis and had to be rescued by Switzerland’s government from potential bankruptcy in November 2008, banks considered this fine to be very high. Yet, despite these measures, the prosecution of UBS deterred only some Swiss banks from violating U.S. law. The two cultural groups strongly differed in their interpretations of and reactions to the U.S. regulators’ enforcement against UBS.

#### The local banks

In response to this event, the locals applied their attribute-based territorial categorization scheme. They attended only in limited ways to the UBS enforcement event and to the international outcries following the accusation of UBS’s assistance in U.S. clients’ tax evasion (OA210). They foregrounded the secondary charges against UBS (the SEC licensing violations) because they were congruent with the taken-for-granted territoriality principle and the according geographical jurisdictional attributes (OA215). Any deviations from the territoriality principle and the jurisdictional implications of the primary charges (i.e., conspiracy) were largely ignored. The locals erroneously thought that the U.S. regulators’ reach encompassed only Swiss-licensed banks’ activities with a presence in the U.S., or a U.S. “nexus” (OA220, OA411), believing that jurisdictions would be mutually exclusive and congruent with states’ geographic borders. The locals refined the geographic attributes that designated a U.S. nexus to include U.S. dollar transfers; bankers’ travel to the U.S.; and communication into the U.S. via (e)mail, phone, and fax (OA260). They believed that Swiss banks that did not use such U.S. jurisdictional means were “purely Swiss” with “no offerings outside the jurisdiction of Swiss law” (OA400) or “subject only to Swiss banking legislation” (OA394, OA410). Only in retrospect, the locals acknowledged that they had “underestimated ‘the extraterritorial reach of the U.S. authorities’” (OA266) and overestimated “Swiss territoriality” (OA146, OA154).

Their geography-focused, attribute-based territorial categorization scheme shaped the local banks’ self-categorizations about whether they were within the U.S. regulators’ reach: “[P]eople used this as an argument that it is a question of whether you go to the U.S., whether you send an e-mail to the U.S. or accept calls from your clients who are in the U.S.—whether you have a U.S. nexus—which triggers this jurisdictional element” (OA220). Based on the U.S. nexus and concepts such as “U.S. jurisdictional means” (OA7) and distinctions between actively seeking U.S. clients versus passively accepting their money (OA273), the locals assessed their location relative to the U.S. regulators’ jurisdiction. They quickly and erroneously concluded that they were not affected. Because the locals had no subsidiaries in the U.S. and most also had never traveled to the U.S., they believed that they had no U.S. nexus. Thus, the locals self-categorized as “outside” the U.S., solely “inside” Swiss jurisdiction (OA342, OA349, OA358, OA406), and “subject only to Swiss banking regulation” (OA394).

Since they self-identified outside U.S. jurisdictional reach (OA560) and as subject exclusively to Swiss law, “protected by Swiss banking secrecy laws” (OA545, OA399), many locals did not assess the risks under U.S. rules (OA520, OA524). And if they did, they concluded that “[t]here is practically no risk if U.S. customers travel to Switzerland and a customer account is handled locally” (OA536), or they considered such risk to be “low” (OA498, OA546, OA573). They emphasized, “We are a legally compliant bank” (OA534), “legal according to Swiss law” (OA532), according to which “tax evasion is not an offense” (OA520, OA524).

The locals’ territorial categorizations and perceptions of safety were largely reinforced by their epistemic authorities, most notably the Swiss regulator, FINMA. In official statements during and after the enforcement event, the Swiss regulator repeatedly emphasized that Swiss sovereignty would remain untouched, no foreign regulator could impose itself on Swiss soil, and “on Swiss territory exclusively Swiss law applies” (OA932). The Swiss regulator concluded that Swiss banks needed to follow foreign legal prescriptions only if the banks had a U.S. “nexus,” were on site abroad, or used “U.S. jurisdictional means” (OA934, OA935), as these means established a physical touchpoint with “U.S. soil” (OA940). They thereby recognized the U.S. regulators’ legal authority on U.S. soil but not beyond it. FINMA, whose mandate is to draw banks’ attention to potential risks, failed to emphasize the risks associated with the U.S. conspiracy statute but emphasized the role of the banks’ U.S. “nexus” for provoking U.S. scrutiny (OA934, OA940).

Further, the locals’ perceptions were reinforced by their interactions with their legal advisors, who also focused primarily on Swiss law, and with like-minded representatives in various associations that focused on inland banks or on private banking, for example (OA944, OA953). They believed that banks without a U.S. nexus that engaged only in passive (not active) U.S. business would be beyond U.S. jurisdictional reach and would not face legal risks (OA946).

#### The cosmopolitan banks

In contrast, the cosmopolitans applied their goal-based territorial scheme to make sense of the enforcement event. Like the locals, most cosmopolitans did not have a major U.S. nexus (neither a U.S. presence nor did they travel to the U.S.). Yet, because they did not take the territoriality principle for granted, they quickly realized that the U.S. regulators’ goal in the UBS enforcement was to stop tax evasion and to prosecute those who assisted in it, no matter where they were located (OA192). This interpretation was reinforced by outcries on the tax evasion issue in the international political environment (OA187, OA190).

Accordingly, the cosmopolitans foregrounded the jurisdictional implications of the primary charges against UBS (the conspiracy statute), rather than the secondary charges (the SEC licensing violations), to assess the U.S. regulators’ jurisdictional reach (OA194, OA196, OA207). They concluded that the U.S. regulators had effectively engaged in extraterritorial enforcement in this case and that any act of conspiracy to defraud the U.S. government would fall under U.S. jurisdiction, even if it took place in Switzerland (OA206). Because banks did not know whether their U.S. clients’ assets were properly taxed, the banks’ business with any U.S. person could expose them to U.S. jurisdictional claims (OA194).

As they realized the U.S. regulators’ protective goal, the cosmopolitans categorized their banks as situated within U.S. jurisdictional reach in the U.S. tax domain. These banks referred both to the conspiracy (OA204) and the tax issue (OA198, OA200) and concluded that they would be within the U.S. regulators’ reach whether or not they had a U.S. nexus. They drew on the *U.S. person* concept to locate their banks in relation to the U.S. regulators’ jurisdiction (OA321, OA323): “Bank22 realized it was within the U.S. jurisdiction. It needed ‘to ensure that its U.S. related customers complied with their U.S. tax obligations’” (OA313).

Recognizing that they were located inside U.S. jurisdiction if they conducted business with any U.S. person, the cosmopolitans also understood the risk that their conduct was noncompliant under U.S. law. With the UBS case, the U.S. authorities had shown that banks’ PWM business with U.S. clients’ untaxed assets could be interpreted as a facilitation of clients’ U.S. tax evasion: “In mid to late 2008, in the wake of the UBS investigation, [Bank4] began to assess the risks of its own U.S. cross-border business. . . . risks that could arise from the unauthorized solicitation of U.S. clients in relation to U.S. law” (OA458). The cosmopolitans also perceived the risk of prosecution by the U.S. regulators as high (OA483, OA487, OA493).

The cosmopolitans’ categorizations of U.S. jurisdictional reach after the UBS enforcement and their threat perceptions were largely reinforced by the actors they relied on for jurisdictional expertise. Unlike the locals, the cosmopolitans were skeptical of the Swiss regulator’s official statements because they perceived that Swiss sovereignty had been violated and that the U.S. would not stop targeting conspirators after the UBS event (OA494). The cosmopolitans involved experts from the more internationally oriented law firms (OA904), other international banks (OA901) or, if they had a foreign owner, from foreign corporate headquarters that were eager to address any U.S.-related risks and their implications for banks. The cosmopolitans’ perceptions of U.S. jurisdictional reach were also largely reinforced by the associations in which these banks participated (OA492, OA896).

#### General deterrence of most cosmopolitans but not of locals

The UBS prosecution had a general deterrence effect on the cosmopolitan banks. Of the 93 unprosecuted banks (see Figure 1), 46 were cosmopolitans, and 42 of those 46 became compliant in Episode 1. Owing to the looming threat of prosecution by the U.S. regulators, the cosmopolitans regarded continuing to serve clients who evaded their U.S. tax obligations as too dangerous (OA479). They decided to comply with U.S. law, requiring their U.S. clients to sign a banking secrecy waiver and declare their assets with IRS W-9 or W-8 forms (OA640, OA660). While many cosmopolitans exited their U.S. clients, some registered as an “investment advisor” in the U.S. in order to fulfill the SEC license obligations (OA647).^7^

In contrast, as the locals felt safe and did not perceive a threat of prosecution after the UBS event, none of the 47 locals became compliant with U.S. law; they all continued to serve U.S. clients without establishing whether their clients’ assets were properly taxed (OA737, OA779). Of the 47 locals, 35 aimed to eliminate any U.S. nexus, such as by introducing explicit travel bans or prohibiting employees from communicating with clients in the U.S., as they thought doing so would keep them outside U.S. jurisdictional reach.

Most locals did not seek to attract the cosmopolitans’ former U.S. clients. But locals with a strong “entrepreneurial approach” (OA822) or explicit “growth strategy” (OA819) regarded these clients as a perfectly legal “business opportunity” and increased client inflow (OA739, OA771, OA774). They began to advertise that, with their “purely Swiss” position (OA400), they offered “legally compliant” solutions for undeclared assets (OA534). According to the U.S. prosecutor, “The message of deterrence generated by the fact that one of the largest and most prominent Swiss banks was being investigated for helping U.S. taxpayers evade their tax obligations simply was not received” (OA738).

### Episode 2: The Extraterritorial Law Enforcement Against Wegelin and Its Extensive General Deterrence in the Swiss Offshore PWM Industry (2011 to 2012)

#### The U.S. regulators

In 2011, following some clients’ voluntary disclosure of undeclared assets, U.S. regulators launched a second extraterritorial enforcement event against the oldest Swiss-licensed bank, Wegelin (Schönig & Straumann, 2023), for “conspiracy” in defrauding the U.S. government in tax matters (OA18). This time, the U.S. regulators clearly communicated the goal-based territorial category of the conspiracy statute and its extraterritorial reach. U.S. attorney Preet Bharara stated in the *United States v. Wegelin* sentencing memorandum (OA16),

Indeed, the prosecution of Wegelin is well within the bounds of the extraterritorial application of federal criminal law. . . . [The U.S.] presumption against extraterritorial application of criminal statutes does not apply to statutes that are “not logically dependent on their locality for the government’s jurisdiction, but are enacted because of the right of the government to defend itself against obstruction, or fraud wherever perpetrated” (citing *United States v. Bowman*, 260 U.S. 94, 98 (1922)). Because Wegelin was assisting U.S. taxpayers in depriving the United States of tax revenue, Wegelin plainly had the aim of causing harm in the United States. Nothing more than this was required to hail Wegelin into a U.S. court.

Unlike in the UBS enforcement, the U.S. regulators did not levy U.S. license violations as a secondary charge against Wegelin because Wegelin had fully contained its activities in Switzerland (it had no U.S. nexus). U.S. regulators thus overrode the Swiss regulator’s and the local banks’ erroneous belief that only banks with a U.S. nexus would be within U.S. reach. Wegelin agreed to pay USD 74 million in fines and restitution to the U.S. authorities (United States Attorney’s Office, 2013). With this event, the U.S. regulators clarified “that the lack of physical presence will never be an impediment to U.S. law enforcement’s acting to protect the IRS’[s] ability to collect revenue from U.S. taxpayers” (OA15).

#### The local banks

The Wegelin enforcement forced the locals to adapt their entrenched territorial categorization scheme. For the locals, the Wegelin enforcement was a “shock” (OA283), a “slap in the face” (OA281), or a “wake-up call” (OA288) that disrupted their taken-for-granted beliefs about the U.S. regulators’ jurisdictional reach and the Swiss regulator’s sovereignty. Following the Wegelin enforcement, 38 other locals acknowledged that the previously assumed “principle of territoriality” (OA290, OA291)—the congruence between state borders and jurisdictions—had been undermined. The event clarified that purely Swiss banks “that were never active on U.S. soil” but that engaged in a conspiracy (OA301, OA293, OA308) with undeclared assets of “U.S. persons” (OA294, OA295) were inside the U.S. regulators’ jurisdiction (OA303).

Based on the *U.S. person* concept, the locals reassessed their position regarding the U.S. regulators’ jurisdiction (OA436). They now realized that their conduct, whether or not it occurred on U.S. soil (OA456), was inside U.S. jurisdiction and was subject to U.S. law if they had direct or indirect (e.g., through external asset managers) relationships with U.S. clients (OA438, OA455). Banks realized that although they “never had a U.S. desk,” never did “business outside Switzerland,” and “did not encourage marketing outside Switzerland” (OA437), they now “would be obligated to identify U.S. persons and share their information with the IRS” (OA436).

After locals realized that their client relationships placed them within the U.S. jurisdiction, they reassessed their risk of prosecution by U.S. regulators as “high” (OA591). First, they assessed the risk of their conduct being noncompliant (OA606). Under the applicable U.S. law (OA588), “the aiding and abetting of tax evasion” constituted a conspiracy risk (OA586). The board minutes of one local bank identified its business with “U.S. customers as a risk” (OA606). Second, the locals acknowledged that the idea to “limit my business to Switzerland only” was misconceived (OA623). They now saw a “latent risk that previous revenues from this U.S. strategy . . . are seized or that corresponding fines are imposed on the bank. Based on the current developments in the industry, the probability of occurrence has increased” (OA609).

The adaptation of the locals’ entrenched jurisdictional schemes and of their threat perceptions was reinforced by the domestic regulator, which no longer foregrounded Swiss sovereignty. In official communications (OA972), the Swiss regulator confirmed the conspiracy statute and its extraterritorial reach (OA966, OA987), suggesting that banks identify their “account holders and/or beneficial owners domiciled in the U.S.A.” (OA617). The domestic regulator now explicitly stated that any Swiss bank could be within the U.S. regulators’ jurisdictional reach and could face legal risks if it conspired in U.S. tax evasion, whether or not it had a U.S. nexus and even if the bank was “only active in Switzerland” (OA989). Among the more domestically oriented law firms that the locals had traditionally worked with, the Wegelin case had similarly triggered a “convergence in legal advice” about U.S. jurisdictional reach (OA992). The locals now also engaged with more internationally oriented legal experts (OA973, OA978). Finally, locally oriented associations also emphasized the U.S. extraterritorial reach and the need to embark on a *white money* strategy—ensuring clients’ compliance with U.S. tax liabilities—to avoid future prosecution risks.

#### General deterrence of (most) locals

Following this enforcement event, 42 previously noncompliant banks became compliant with U.S. law: the four cosmopolitans that had not complied in Episode 1, along with 38 locals; see Figure 1.^8^ With their changed threat perceptions, most locals now regarded noncompliance as an option that was no longer viable. They decided to require IRS W-8/W-9 forms in order to stop facilitating U.S. clients’ tax evasion (OA826, OA832). The U.S. regulators’ general deterrence message to Swiss banks had taken effect.

## Theory Development: Territorial Self-Categorization and Cross-Border General Deterrence

To explain the observed variations in Swiss banks’ compliance with U.S. law following the two U.S. extraterritorial law enforcement events, we inductively developed an interpretive theory of cross-border general deterrence. Our theory integrates deterrence research with a novel organization-centered and cultural–cognitive perspective on regulatory territory, which we developed from our data and by blending constructivist research on territory from international relations and international law (e.g., Agnew, 1994; Berman, 2002; Buxbaum, 2009; Krasner, 1988; Sassen, 2013) with cultural–cognitive perspectives on categories and categorization (DiMaggio, 1997; Durand et al., 2017; Glynn & Navis, 2013).

### Introducing Our Novel Theoretical Framework

**Figure 3.**
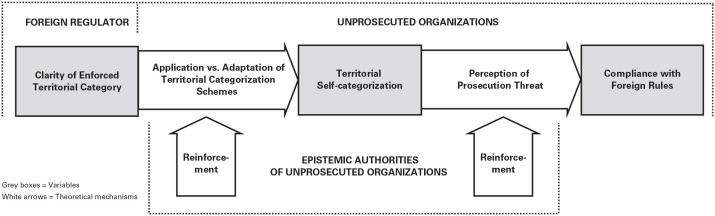
Theoretical Framework

Our novel theory (Figure 3) unpacks the role of what we call the unprosecuted organizations’*territorial self-categorization*, its antecedents, and its general deterrence outcomes. Our theory is based on variables and mechanisms that we induced from our data (see Figure 2). Table 6 summarizes how they are represented in the two episodes of our case.

**Table 6.** Representation of Derived Concepts in both Episodes

Key Actors	Variables and Mechanisms	Episode 1		Episode 2
Foreign regulator	Clarity of enforced territorial category	Low		High
Unprosecuted organizations	Application vs. adaptation of territorial categorization schemes	Locals: Application of attribute-based territorial categorization schemes	Cosmopolitans: Application of goal-based territorial categorization schemes	Locals: Adaptation to goal-based territorial categorization schemes
	Territorial self-categorization	Locals: Attribute-based territorial self-categorization (outside)	Cosmopolitans: Goal-based territorial self-categorization (inside)	Locals: Goal-based territorial self-categorization (inside)
	Perception of prosecution threat	Locals: Perception of safety	Cosmopolitans: Perception of prosecution threat	Locals: Perception of prosecution threat
	Compliance with foreign rules	Locals: Noncompliance	Cosmopolitans: Compliance	Locals: Compliance
Epistemic authorities	Reinforcement	Epistemic authorities of locals: Reinforcement of attribute-based territorial categorizations and safety perceptions	Epistemic authorities of cosmopolitans: Reinforcement of goal-based territorial categorizations and threat perceptions	Epistemic authorities of locals: Reinforcement of goal-based territorial categorizations and threat perceptions

We propose that, when faced with a foreign regulator’s extraterritorial law enforcement against a peer organization, unprosecuted organizations engage in territorial self-categorization—a type of spatial self-categorization (cf. Huttenlocher et al., 1991) by which they assess their location in relation to regulatory territory—which is a key factor for cross-border general deterrence. Such self-categorizations may be either attribute-based or goal-based. Attribute-based territorial self-categorization may lead organizations outside the foreign regulator’s geographic borders to conclude that they are also outside the foreign regulator’s territorial category (as the locals did in Episode 1). But if unprosecuted organizations engage in goal-based territorial self-categorization (i.e., based on the foreign regulators’ enforced goal-based territorial category), some organizations whose activities are outside the foreign regulator’s geographic borders may see themselves as inside the latter’s territory. This occurred for the cosmopolitans in Episode 1 and for the locals in Episode 2. But as long as organizations engage only in attribute-based territorial self-categorization, as the locals did in Episode 1, they may regard themselves as outside the foreign regulators’ reach.

Territorial self-categorization critically explains cross-border general deterrence because it shapes unprosecuted organizations’ perceptions of a prosecution threat. As the deterrence literature (e.g., Nagin, 2013) emphasizes, such perceptions critically shape legal compliance. These threat perceptions have two dimensions: a perceived threat of organizational illegality (i.e., the risk of violating foreign regulators’ legal rules) and a perceived threat of prosecution (i.e., the risk of facing law enforcement by foreign regulators). An unprosecuted organization may perceive a high threat of prosecution for legal violations by foreign regulators only if it categorizes itself as being inside the foreign regulators’ territory. Only then will the organization accept the foreign regulators’ rules as valid and binding, and realize that it faces a prosecution threat if it violates these rules (Dornbusch & Scott, 1975; Weber, 1978). As long as organizations (such as the locals in Episode 1) self-categorize as being located outside foreign regulators’ territory, they may perceive a low prosecution risk and remain noncompliant with foreign rules.

Regarding the antecedents of territorial self-categorization, our cultural–cognitive perspective suggests that unprosecuted organizations may engage in territorial self-categorizations differently depending on two factors: (1) the level of clarity with which a foreign regulator communicates the goal-based territorial category that underpins its extraterritorial law enforcement and (2) the unprosecuted organizations’ entrenched territorial categorization schemes. For the first factor, we found that regulators can mobilize and communicate a goal-based territorial category in an extraterritorial law enforcement act with more or less clarity. Such clarity involves the extent to which regulators make the regulatory goal that underpins their territorial categorization explicit and transparent in an enforcement event (see Harmon, 2018), and it also entails the absence of ambiguity and distractions (Connelly et al., 2010). In our case, this clarity was low in enforcement Episode 1 (UBS) and high in Episode 2 (Wegelin).

Considering the second factor, we divided the organizations in our study sample into *locals* and *cosmopolitans*. Following their inward orientation (cf. Levy et al., 2016; Merton, 1957) that foregrounds the domestic regulator as their primary epistemic authority (cf. Kruglanski, 1989; Loveman, 2005), locals (initially) mobilized the highly taken-for-granted *attribute-based* territorial categorization scheme. Geographic attributes circumscribe the scope of a regulator’s territory in this scheme, which is grounded in an idealistic view of international order (cf. Zuolo, 2012) according to which regulators respect the territoriality principle as a shared international rule and the domestic regulators’ sovereignty. In contrast, cosmopolitans have a more emancipated relationship to the domestic regulator as epistemic authority. Given their greater receptiveness to foreign regulators’ territory claims (cf. Beck, 2006; Hannerz, 1990; Merton, 1957), they mobilized a *goal-based* territorial categorization scheme that emphasizes foreign regulators’ interests to circumscribe the scope of their territory, unconstrained by geographical attributes. This scheme is grounded in a realist view of international order whereby powerful states may enact their goals beyond geographic boundaries (Krasner, 1999).

When foreign regulators communicate their goal-based territorial category with *low clarity* in the extraterritorial law enforcement event, locals may fail to adapt their entrenched attribute-based territorial categorization scheme. This insight is in line with cultural–cognitive perspectives, which suggest that cultural schemas serve as powerful filters that shape actors’ interpretations if their informational environment exhibits high ambiguity and uncertainty (DiMaggio, 1997; Edelman, 1992; Scott, 2014; Suchman, 1997). Consequently, locals may (erroneously) self-categorize as outside a foreign regulator’s territorial reach, perceive low or no threat of prosecution, and remain noncompliant. Specifically, if locals take their attribute-based territorial categorization scheme for granted, this scheme may not only constrain their rational choices and consequentialist assessments (e.g., Ingram & Clay, 2000) but may also override and suppress them. Because the organizations may not perceive alternatives to their entrenched beliefs (cf. Esser and Kroneberg, 2015), the threat posed by foreign regulators may remain virtually invisible to them.

Locals may overcome their cultural–cognitive inertia (see Seo & Creed, 2002) and territorial myopia only if a foreign regulator communicates its goal-based territorial category with *high clarity*. Such clarity forces locals to adapt their attribute-based territorial categorization scheme and to adopt a foreign regulator’s goal-based territorial category. This adaptation is a bottom-up, inductive approach to territorial categorization (cf. Porac & Thomas, 1990), whereby attribute-based schemas are altered into goal-based ones to fit the new information. Only then will locals self-categorize as being inside a foreign regulator’s territory, perceive a high prosecution risk, and become compliant with foreign rules.

In contrast, cosmopolitans may already apply their goal-based territorial self-categorization when clarity is low. They may thus self-categorize as inside a powerful foreign regulator’s territory, perceive a high threat of prosecution, and become compliant with foreign rules. Obviously, we also suggest that cosmopolitans may respond similarly when clarity is high.

Finally, we propose that cosmopolitans and locals rely on distinct epistemic authorities (Kruglanski, 1989; Zürn, 2018). In our setting, the locals relied on the domestic regulator as the ultimate epistemic authority, on domestically oriented legal advisors, and on other locals. In contrast, the cosmopolitans relied on internationally oriented legal advisors and on other cosmopolitans. This finding aligns with research suggesting that actors tend to embed themselves in communities of similar others (Levy et al., 2016; McPherson et al., 2001; Rey et al., 2021). Yet, as their epistemic authorities may share the unprosecuted organizations’ own interpretations, these authorities may have a reinforcing (rather than disruptive) effect on the organizations’ territorial categorizations and threat perceptions (cf. McDonald & Westphal, 2003).

### Alternative Explanations

Drawing on different literatures, including deterrence, neo-institutionalism, and social comparison, we conducted extensive supplementary qualitative and quantitative analyses to account for possible alternative explanations for our findings (see Online Appendix II). We assessed whether there were important differences between the two U.S. law enforcement events^9^ and between the two groups of banks^10^ that could explain the differences in their responses. We could thus rule out the possibility that our findings can be explained with existing theory. These analyses further legitimated our inductive and grounded theory-building approach (cf. Edmondson & McManus, 2007).

## Discussion

### Contributions to the Research on Organizations’ Legal Environment

We have shown that organizations’ regulatory environment can be conceived as a *territory*. We have introduced the *regulatory territory* concept into the research on organizations’ regulatory environment (e.g., Djelic & Quack, 2018; Edelman & Suchman, 1997; Schneiberg & Bartley, 2008) and to organization studies generally. The organizational research has treated a state’s regulatory territory as a given—as fixed and geographically defined by a state’s boundaries, as if it were a naturalistic and largely invariable attribute of the state (e.g., Edelman & Suchman, 1997; Guillén, 2001; Schneiberg & Bartley, 2008; Scott, 2017). International relations and international law scholars conceive of states’ regulatory territories as the result of regulators’ territory claims, for instance, in their law enforcement actions (see Agnew, 1994; Buxbaum, 2009; Raustiala, 2005; Ruggie, 1993; Sassen, 2013). However, both conceptions are insufficient: A regulator’s *manifest* territory (cf. Wrong, 1979) is neither necessarily geographically fixed nor merely the result of regulators’ territory claims. Instead, it depends on whether the firms in question—the cosmopolitans and the locals (cf. Beck, 2006; Levy et al., 2016; Merton, 1957)—self-categorize as inside what they perceive as the regulator’s territory. Only then do they realize that they cannot violate legal rules without threats of prosecution (Dornbusch & Scott, 1975).

This conception of regulatory territory opens new avenues for research on states’ cross-border legal coercion and deterrence (e.g., Gunningham et al., 2005; Reid & Toffel, 2009; Short & Toffel, 2010). It emphasizes that states’ legal coercion—and not only *soft law* mechanisms mobilized by transnational and private regulatory actors (cf. Djelic & Quack, 2018; Scherer & Palazzo, 2011; Vogel, 2008)—plays a role in organizations’ transnational governance. We have proposed territorial self-categorization as a necessary mechanism for cross-border general deterrence and as a driver of organizations’ perceptions of prosecution risk. Owing to the assumption in the research of fixed, geographically defined regulatory territories, territorial self-categorization may have been considered self-evident and thus not in need of explicit study. Yet, in order to explain general deterrence in settings in which this assumption does not hold, this self-categorization becomes central.

We have also emphasized that deterrence is critically mediated by cultural–cognitive mechanisms. We responded to calls to reconcile the deterrence and neo-institutional perspectives and to develop a more “integrative model” for explaining organizations’ legal (non)compliance (Edelman & Suchman, 1997: 507). The deterrence research has foregrounded organizational responses to law enforcement based on a logic of consequences (Short, 2013). The neo-institutional research has emphasized organizations’ taken-for-granted beliefs and a logic of appropriateness, focusing on the introduction of new laws that have remained unenforced (see Davis & Greve, 1997; Dobbin & Sutton, 1998; Edelman, 1992; Suchman & Edelman, 1996; Zhang, 2022). Both perspectives have evolved separately with little cross-fertilization (Edelman & Suchman, 1997; Short, 2013; Vaughan, 1998). However, each perspective provides only “a partial account of the forces at work” in firms’ responses to their legal environments (Hall & Taylor, 1996: 955). We have shown how organizations’ entrenched cultural beliefs about the legal environment may constrain or even suppress their rational choices and threat perceptions following law enforcement events (cf. Esser & Kroneberg, 2015; Ingram & Clay, 2000).

### Contributions to Organizational Research on Categories

Our study makes several contributions to the organizational literature on categories. First, we have advanced territorial categories as a novel regulatory category type (Funk & Hirschman, 2014; Ozcan & Gurses, 2017). Extant research has focused on how the regulatory categorizations of law enforcement authorities and other social control agents demarcate the line between right and wrong (or between legal and illegal) organizational conduct (Edelman & Suchman, 1997; Greve et al., 2010; Norton, 2014; Palmer et al., 2016). Our focus on territorial categorizations suggests that regulatory categorizations involve not one but two dimensions: not only *what* occurred (i.e., the conduct type, as [non]compliant) but *where*. Thus, actors considered compliant may become classified as wrongdoers not only following a shift in the line between legal and illegal behaviors but also following a control agent’s expanded territorial reach.

Our study also adds to the research on category membership of firms, which prior research derived primarily from aggregated product features (e.g., Arjaliès & Durand, 2019; Hsu & Grodal, 2021; Syakhroza et al., 2019). Our study shows that organizations’ self-categorization shapes their behavior and product characteristics. Hence, categories act as judgment devices not only because they entail value-laden and moral attributes but also because organizational self-categorization obeys higher-order normative principles, such as the territoriality or the protective principle. These principles define the legal (or moral) domain as enforceable or not and represent the cultural–cognitive bases of strategic groups and competitive boundaries in the legal space (cf. Cattani et al., 2017; Cattani et al., 2024; Porac et al., 1995).

Further, our study contributes to research on goal-based categories (e.g., Boghossian & David, 2021; Durand & Boulogne, 2017; Durand & Paolella, 2013), particularly on the conditions in which they elicit the intended effect in an organizational field (cf. Glaser et al., 2019). Our study suggests that cultural–cognitive factors may critically mediate the prediction that low information clarity may enable goal-based categories to provoke their intended effect (Durand & Paolella, 2013). In our case, only the cosmopolitans, whose cultural schemes were aligned with the enforced goal-based category, complied in conditions of low clarity. To achieve this effect among the locals, whose entrenched schemes were incompatible with the enforced category, high clarity was needed for them to adapt their categorization schemes. Hence, in contrast to extant research conceiving of goal-based categories as ad-hoc categories (cf. Barsalou, 1983; Porac et al., 1999), we found that they are grounded in taken-for-granted cultural schemes (cf. DiMaggio, 1997; Durand & Thornton, 2018).

### Generalizability and Future Research

Our framework may be generalizable, first, to instances of extraterritorial enforcement by other national regulators and supranational authorities with sufficient coercive power, such as Canada, China, the U.K., the EU, and large EU countries (e.g., Kaczmarek & Newman, 2011; Kalyanpur & Newman, 2018). The framework helps to illuminate how regulators enforce law based on different extraterritorial principles of jurisdiction (cf. Fichtelberg, 2014; Ryngaert, 2015; Shaffer et al., 2014) to deter organizations from committing various offenses. Some of these offenses include corruption (Kaczmarek & Newman, 2011), human rights violations (Deva, 2021; Reinecke & Ansari, 2016; Schrempf-Stirling & Wettstein, 2017), environmental crimes (Vagliasindi, 2019), organized crime (Slattery, 2018), cybercrime (Hildebrandth, 2013), or corporate trade with countries under sanctions, rogue regimes, or enemies of the state (e.g., Gaur et al., 2023; Mallard & Sun, 2022; Meyer, 2009), especially if these offenses are not criminalized in the locations where they are committed.^11^ Our study can further inform research on organizations operating in seemingly nonterritorial settings, such as offshore, cyberspace, outer space, the high seas, or the deep sea (cf. Kobrin, 2001; Munro, 2016; Ruggie, 1993). Such settings may motivate firms to evade regulatory demands and regulators to enforce novel goal-based territorial categorizations to govern them.

Our theory can help to conceptualize governance attempts by other social control agents, e.g., professional/religious authorities and nongovernmental or supranational organizations (e.g., Djelic & Sahlin-Andersson, 2006; Schneiberg & Bartley, 2008; Vogel, 2008). Such agents may likewise attempt to enforce territorial categorizations, claiming that rules are valid in certain spaces, and seek to deter organizations from transgressing these rules through soft or hard enforcement.

In our case, the locals (initially) mobilized the largely taken-for-granted attribute-based categorization scheme, and the cosmopolitans mobilized a goal-based scheme that drove their compliance decisions. A core assumption of our model is that organizations follow through on their self-categorizations. Future research could investigate further variations within these groups’ territorial self-categorizations. As we identified from our analyses of the few outliers (see footnotes 6 and 7), some organizations self-categorize as inside but remain noncompliant due to other factors affecting prosecution threat perceptions, e.g., being too small, being protected by a particular status, having too few U.S. clients, or the self-categorizations not being sufficiently shared among key decision makers and compliance professionals. Competing enforcement issues may also constrain compliance decisions.

More generally, our framework could also inform scholarship on spatial categorizations (cf. Huttenlocher et al., 1991; Tversky, 2003). It should help scholars study interpretive processes of demarcating social and geographic spaces, organizational locations within such spaces, and the rules and norms that govern them. These spaces may be located within but may also reach across national borders whereby, just like in our study, membership in such spaces may be both attribute- and goal-based (cf. Durand & Paolella, 2013). The normative rules guiding spatial governance need not necessarily be legal in nature but may also be based on other types of values and ethical criteria (e.g., Arjaliès & Durand, 2019; Syakhroza et al., 2019).

Moreover, since territory has been generally defined as the spatial scope of an actor’s power (Storey, 2012), our framework could help scholars study other types of territories beyond legal and moral ones. These may include the spheres of influence of firms (D’Aveni, 2004), employees (Brown et al., 2005), professions (Abbott, 1988), local communities (Bergemann & Brandtner, 2024), criminal organizations (Vaccaro & Palazzo, 2015), social movements (Guidry et al., 2000), or states during geopolitical dynamics (e.g. Witt, 2019). In these cases, organizational actors may engage in territorial self-categorizations to assess the scope of such territories and their location in relation to these territories.

### Boundary Conditions

We acknowledge that we observed a very high general deterrence effect in both episodes, which may be explained by the banks’ intention to conduct jurisdictional arbitrage. Once the locals realized that they were within U.S. jurisdiction, this arbitrage opportunity vanished. Results may differ if noncompliance is based on other motives. Moreover, in terms of the domestic regulator, we witnessed the typical scenario whereby the regulator seeks to maintain the myth of its sovereignty for as long as possible (see Biersteker, 2013; Krasner, 1999). Future research could investigate more-atypical scenarios in which the domestic regulator maintains sovereignty claims even in the presence of clear competing claims or withdraws them earlier. With regard to organizations’ epistemic authorities, we observed a strong similarity in orientation between each group of banks (locals and cosmopolitans) and its respective communities and advice networks. Future research may further investigate situations in which the organizations and their epistemic authorities’ orientations conflict with each other. Finally, in our case, the international political environment endorsed U.S. extraterritorial law enforcement. Future research may further investigate organizations’ interpretations and responses when the international political environment disapproves of such enforcement acts, for instance, due to their violation of international principles or because of geopolitical tensions and rivalries.

## Supplemental Material

sj-pdf-1-asq-10.1177_00018392251334366 – Supplemental material for Regulatory Territory and General Deterrence Across Borders: Swiss Banks’ Territorial Self-Categorizations and Responses to U.S. Extraterritorial Law Enforcement Supplemental material, sj-pdf-1-asq-10.1177_00018392251334366 for Regulatory Territory and General Deterrence Across Borders: Swiss Banks’ Territorial Self-Categorizations and Responses to U.S. Extraterritorial Law Enforcement by Emmanuelle Reuter, Florian Überbacher and Andreas Georg Scherer in Administrative Science Quarterly

## Footnotes

1 Extraterritorial law enforcement differs from conventional transnational governance types that focus on less coercive or *soft law* mechanisms by nonstate actors (e.g., Djelic & Quack, 2018; Scherer & Palazzo, 2011; Vogel, 2008). It also differs from other transnational policy diffusion processes wherein foreign actors target states and public policymakers (e.g., Dobbin et al., 2007; Halliday & Carruthers, 2007) rather than companies and their members directly.

2 The banks had one of three business model types: *Pure players* (26 banks) focus on private banking only, *hybrids* (34 banks) additionally focus on asset management, and *universals* (33 banks) additionally focus on investment and retail banking activities. The sample included banks in different headquarter locations across Switzerland and reflected a wide range of firm sizes, ages, legal statuses, and ownership types.

3 Depot accounts (also securities or custody accounts) are established for the management of financial securities, and sometimes to obscure income from tax authorities, by exploiting the confidentiality associated with these accounts. Coded accounts are financial accounts that assign a unique number (rather than the account holder’s name) to an account sometimes to conceal the identity of the true owner. Shell structures enclose sometimes untaxed assets in shell companies often without active business operations and sometimes to conceal the identity of the true owner. Insurance wrappers bundle sometimes untaxed assets in an insurance cover.

4 In a 1924 case, *Hammerschmidt v. United States*, Chief Justice William Taft defined *defraud:*“To conspire to defraud the United States means primarily to cheat the Government out of property or money,” he said. “But it also means to interfere with or obstruct one of its lawful governmental functions by deceit, craft or trickery, or at least by means that are dishonest” (BBC, 2017).

5 As these five banks also came under investigation after the Wegelin prosecution, the mechanism of compliance was not one of general deterrence.

6 The IRS W-9 (W-8) form confirms clients’ U.S. (non-U.S.) tax status toward the IRS.

7 The four cosmopolitan banks that remained noncompliant acknowledged the extraterritorial U.S. reach but thought they would remain under the radar of U.S. regulators (Interview 53). They became compliant in Episode 2.

8 There were nine locals (of 47 total in our sample) that we did not record as deciding to comply during Episode 2. One was Wegelin. Five other locals were eventually investigated. That leaves three locals—two small community-oriented banks with a very limited number of U.S. clients, and one private bank that also faced several other enforcement issues—that all became compliant after our observation window (in 2013, when the U.S. and Swiss regulators jointly announced a program that enabled all Swiss banks to settle in nonprosecution agreements).

9 The comparison was based on the sanctions’ severity, the U.S. regulators’ access to evidence, their coercive capacity, the clarity of the goal-based territorial categorization, and the prosecution events’ salience.

10 We conducted bivariate as well as binary logistic regression analyses to assess whether membership in the compliant and noncompliant groups was based on the following variables: percentage of U.S. assets, percentage of U.S. accounts, status similarity, business model similarity, geographic similarity, firm age, firm size, prominent firms, and ownership type.

11 Recent examples of extraterritorial jurisdiction include U.S. (secondary) sanctions on foreign firms constructing the Nord Stream 2 gas pipeline in the Baltic Sea between Russia and Germany (Eckert, 2020); foreign firms doing business with Russia (Ren, 2022); human rights violations in China’s Xinjiang Uyghur Autonomous region (Brunnstrom, 2020; Uyghur Forced Labor Prevention Act, 2020); Chinese tech firms (Swanson et al., 2020); European firms trading with Iran (Chellaney, 2018; Geranmayeh & Rapnouil., 2019); the EU’s extraterritorial expansion of its data protection regulations that aim to cover foreign internet firms (Court of Justice of the European Union, 2020); or China’s new Hong Kong Security Act (Elegant, 2020) and its provisions against so-called *unreliable entities*—foreign companies that allegedly threaten China’s national security with their conduct outside China (Rachman, 2020).
