# Supplementary material for: Regulatory Territory and General Deterrence Across Borders: Swiss Banks’ Territorial Self-Categorizations and Responses to U.S. Extraterritorial Law Enforcement
Source: Adm Sci Q. 2025 May 7;70(3):821–64. doi: 10.1177/00018392251334366 (PMC12301513; doi:10.1177/00018392251334366)
Supplement: sj-pdf-1-asq-10.1177_00018392251334366 – Supplemental material for Regulatory Territory and General Deterrence Across Borders: Swiss Banks’ Territorial Self-Categorizations and Responses to U.S. Extraterritorial Law Enforcement [file sj-pdf-1-asq-10.1177_00018392251334366.pdf]

REGULATORY TERRITORY AND GENERAL DETERRENCE ACROSS BORDERS: SWISS BANKS' TERRITORIAL SELF-CATEGORIZATIONS AND RESPONSES TO U.S. EXTRATERRITORIAL LAW ENFORCEMENT

ONLINE APPENDICES

Online Appendix I: Additional Empirical Evidence for Codes .... **Page 2**

Approx. 990 numbered empirical quotes in support for each of the codes in our data structure.

Online Appendix II: Alternative Explanations .... **Page 52**

Qualitative and quantitative exploration of alternative theoretical explanations of our findings.

ONLINE APPENDIX I: ADDITIONAL EMPIRICAL EVIDENCE FOR CODES

Please note that excerpts of the quotes marked in 'grey' are also used in the "Table 4. Exemplary evidence for codes" of the paper.

Actors and Episodes	Quote No.	Codes and Quotes
		**FOREIGN REGULATOR: CLARITY OF ENFORCED TERRITORIAL CATEGORY**
**EPISODE1**		**FOREIGN REGULATOR: CLARITY OF ENFORCED TERRITORIAL CATEGORY**
		**Explicitness of regulatory goal**
	OA1	"...charging UBS with *participating in a conspiracy to defraud the United States* and its agency the Internal Revenue Service ("IRS") in violation of 18 U.S.C. § 371." (United States District Court Southern District of Florida, CASE NO. 09-60033-CR-COHN, DPA: 1, *emphasis added*)
	OA2	"Beginning in 2000 and continuing until 2007, UBS, through certain private bankers and managers in the United States cross-border business, *participated in a scheme to defraud the United States and its agency, the IRS, by actively assisting or otherwise facilitating a number of United States individual taxpayers in establishing accounts at UBS in a manner designed to conceal the United States taxpayers' ownership or beneficial interest in these accounts*. In this regard, these private bankers and managers facilitated the creation of accounts in the names of offshore companies, allowing United States taxpayers to evade reporting requirements and to trade in securities as well as other financial transactions (including making loans for the benefit of, or other asset transfers directed by, the United States taxpayers, and using credit or debit cards linked to the offshore company accounts)." (United States District Court Southern District of Florida, CASE NO. 09-60033-CR-COHN, DPA: 1, *emphasis added*)
	OA3	"Raoul Weil, a senior executive of a large Swiss bank with offices worldwide, including the United States, has been charged with *conspiring with other executives, managers, private bankers and clients of the banking firm to defraud the United States*, the Justice Department and Internal Revenue Service (IRS) announced today. According to the criminal indictment, between 2002 and 2007, Weil oversaw the Swiss bank's cross-border private banking business that provided services to some 20,000 U.S. clients who reportedly *concealed* approximately $20 billion in *assets from the IRS*. Weil, who allegedly referred to this business as "toxic waste," mandated that Swiss bankers grow the cross-border business, despite knowing that this would cause bankers to violate U.S. law." (DOJ, 20081112: 1, *emphasis added*)
	OA4	"UBS AG, Switzerland's largest bank, has entered into a deferred prosecution agreement on *charges of conspiring to defraud the United States* by impeding the Internal Revenue Service (IRS) (...) UBS has further agreed to pay $780 million in fines, penalties, interest and restitution. (...) In November 2008, UBS executive Raoul Weil was indicted by a federal grand jury in Fort Lauderdale and *charged with conspiring to defraud the United States* for his alleged role in overseeing the United States crossborder business. (...) In June 2008, former UBS private banker Bradley Birkenfeld pleaded guilty to a *charge of conspiring to defraud the United States* for similar conduct." (DOJ, 20090218: 1-2, *emphasis added*)
	OA5	"This kind of conduct, which actively facilitates tax evasion, amounts to a declaration of war by offshore secrecy jurisdictions against honest, hardworking taxpayers. We are determined to fight back and end the abuses inflicted on us by those tax havens... UBS is headquartered in Switzerland and is one of the largest banks in the world. During our July hearing, UBS admitted publicly for the first time that an estimated 19,000 U.S. clients had opened UBS accounts in Switzerland with nearly $18 billion in assets that were not disclosed to the U.S. Internal Revenue Service (IRS). Since then, new evidence suggests that there may be far more than 19,000 U.S. clients with hidden accounts at that Swiss bank. A 2004 UBS internal report, which was introduced in court by the United States and we have marked as Exhibit 12,2 analyzes the U.S. client accounts opened in Switzerland. It states: 'The number of account relationships in WM&BB in Switzerland with U.S. residents where the account holder has not provided a W–9 is approximately 52,000 (representing CHF 17 billion''—which means 17 billion Swiss francs—''in assets).'" (PSI, 2009: 2)
	OA6	"The disclosure of the UBS invetigation presented [banks] with a stark choice…" They "could have been deterred by the Department of Justice's tax-related investigation of UBS and concluded that it should exit the business of assisting U.S. taxpayers in evading taxes." (United States District Court Southern District of New York, S1 12 Cr. 02 (JSR): 6)

Presence of distractions

OA7 "The SEC also alleges that UBS conducted that cross-border business largely through client advisers located primarily in Switzerland, who were not associated with a registered broker-dealer or investment adviser. These client advisers *traveled to the U.S., on average, two to three times per year* on trips that generally varied in duration from one to three weeks. In many instances, the client advisers attended exclusive events such as art shows, yachting events, and sporting events that were often sponsored by UBS, for *the purpose of soliciting and communicating with United States cross-border clients*. UBS also used other *U.S. jurisdictional means such as telephones, facsimiles, mail and e-mail to provide securities services to its U.S. cross-border clients*." (U.S. Securities and Exchange Commission, Litigation Release No. 20905 / February 18, 2009)

OA8 The SEC further alleges that "UBS was *aware that it was required to be registered with the SEC*. UBS *took action to conceal its use of U.S. jurisdictional means to* provide securities services. Among other things, client advisers typically *traveled to the U.S.* with encrypted laptop computers that they used to provide account-related information, to show *marketing materials* for securities products (...) As charged in the SEC's complaint, as a result of its conduct, UBS violated Section 15(a) of the Securities Exchange Act of 1934 and Section 203(a) of the Investment Advisers Act of 1940." (U.S. Securities and Exchange Commission, Litigation Release No. 20905 / February 18, 2009)

OA9 "In connection with the establishment of these offshore company accounts, UBS private bankers and managers accepted and included in UBS's account records IRS Forms W- 8BEN (or UBS's substitute forms) provided by the directors of the offshore companies which represented under penalty of perjury that these companies were the beneficial owners, for United States federal income tax purposes, of the assets in the UBS accounts. In certain cases, the IRS Forms W-8BEN (or UBS's substitute forms) were false or misleading in that the United States taxpayer who owned the offshore company actually directed and controlled the management and disposition of the assets in the company accounts and/or otherwise functioned as the beneficial owner of the assets in disregard of the formalities of the purported corporate ownership. *Additionally*, these private bankers and managers would actively assist or otherwise facilitate certain undeclared United States taxpayers, who these private bankers and managers knew or should have known were evading United States taxes, by meeting with these clients in the United States and communicating with them via United States jurisdictional means on a regular and recurring basis with respect to their UBS undeclared accounts." (United States District Court Southern District of Florida, CASE NO. 09-60033-CR-COHN, DPA: 2, emphasis added)

OA10 "It was further part of the conspiracy that Managers, Desk Heads, and Bankers solicited new investments in the Cnited States cross-border business by marketing UBS secrecy to Cnited States clients interested in attempting to evade United States income taxes, in particular by claiming that Swiss bank secrecy was impenetrable." (United States District Court Southern District of Florida, CASE NO. 09-60033-CR-COHN, DPA: 6, emphasis added)

OA11 "...undeclared United States taxpayers who were actively assisted or facilitated by UBS private bankers who met with these clients *in the United States* and communicated with them *via United States jurisdictional means* on a regular and recurring basis as described in paragraph 4 of the Statement of Facts (as agreed to more fully in a separate letter between the IRS and UBS)." (United States District Court Southern District of Florida, CASE NO. 09-60033-CR-COHN, DPA: 3, *emphasis added*)

OA12 "Desk Heads *traveled to the United States* to conduct *unlicensed* banking and investment advisory activity for UBS's United States clients. (...) These Bankers were *not licensed to engage in banking and investment advisory activity in the United States*. However, these Bankers routinely *traveled to the United States* to conduct unlicensed banking and investment advisory activity for UBS's United States clients. (...) It was a part and an object of the conspiracy that defendant UBS and its coconspirators would and did increase the profits of UBS by providing *unlicensed* and *unregistered* banking services and investment *advice in the United States* and other activities intended to conceal from the IRS the identities of UBS's United States clients (....) It was further part of the conspiracy that Managers, Desk Heads, and Bankers provided *unlicensed* and *unregistered* banking services and investment advice to United States clients in person while *on travel to the United States* and *by mailings, mail, and telephone calls to and from the United States.*" (United States District Court Southern District of Florida, CASE NO. 09-60033-CR-COHN, DPA: 3-6, *emphasis added*)

OA13 "The information also alleges that Swiss bankers *routinely traveled to the United States* to market Swiss bank secrecy to United States clients interested in attempting to evade United States income taxes. Court documents assert that, in 2004 alone, Swiss bankers *allegedly traveled to the United States* approximately 3,800 times to discuss their clients' Swiss bank accounts. The information further alleges that UBS managers and employees used encrypted laptops and other counter-surveillance techniques to *help prevent the detection of their marketing efforts* and the identities and offshore assets of their U.S. clients. According to the information, clients of the cross-border business in turn filed false tax returns which omitted the income earned on their Swiss bank accounts and failed to disclose the existence of those accounts to the IRS."" (DOJ, 20090218: 1, *emphasis added*)

OA14 "[W]e had a very large group of people in Lugano, Geneva, and Zurich that *marketed directly into the U.S. market.* The private bankers would *travel* anywhere between four and six times a year to the U.S., spend anywhere from one to two weeks in the U.S., prospecting, visiting existing clients, so on and so forth. ... As I remember, there [were] around 25 people in Geneva, 50 people in Zurich, and five to ten in Lugano." (Birkenfeld deposition, 07/10/11: 46-48, *emphasis added*)

EPISODE2 **FOREIGN REGULATOR:**

CLARITY OF ENFORCED TERRITORIAL CATEGORY

Explicitness of regulatory goal

OA15 With the Wegelin case, U.S. authorities intended to "...send a message of deterrence to banks (...) who would believe that, without a physical presence in the United States, they cannot be reached by U.S. law enforcement. (...) [T]he forfeiture of funds in Wegelin's

		account *sends the message that the lack of physical presence will never be an impediment to U.S. law enforcement's acting to protect the IRS' ability to collect revenue from U.S. taxpayers*." (DOJ130225, 2013, *emphasis added*)
	OA16	"Indeed, the prosecution of Wegelin is well within the bounds of the *extraterritorial application of* federal criminal law. Our Constitution permits, consistent with constitutional due process, the extraterritorial application of federal criminal law to *non-citizens acting entirely abroad 'when the aim of that activity is to cause harm within the United States or to U.S. citizens or interests.'"* United States v. Al Kassar, 660 F.3d 108, 118 (2d Cir. 2011); see also United States v. Mardirossian, 818 F. Supp. 2d 775, 776 (S.D.N.Y. 2011) (*noting that presumption against extraterritorial application of criminal statutes does not apply to statutes that are "not logically dependent on their locality for the government's jurisdiction, but are enacted because of the right of the government to defend itself against obstruction, or fraud wherever perpetrated*") (citing United States v. Bowman, 260 U.S. 94, 98 (1922)). Because Wegelin was assisting U.S. taxpayers in depriving the United States of tax revenue, *Wegelin plainly had the aim of causing harm in the United States*. *Nothing more than this was required to hail Wegelin into a U.S. court.*" (Rakoff In DOJ, 20130225: 14, *emphasis added*)
	OA17	"'This prosecution *serves notice that the Department of Justice will not tolerate fraudulent activity designed to undermine the integrity of our income tax system*,' said U.S. Attorney for the District of Arizona John S. Leonardo." (DOJOPA, 2013, *emphasis added*)
	OA18	"[S]ince 2009, aside from UBS, DOJ has indicted only one Swiss bank, Wegelin & Co**.** (...) which eventually pled guilty to *conspiracy to defraud the United States of tax revenue...*" (PSI, 20140226: 5, *emphasis added*)
	OA19	"[A] foreign bank has been *indicted for facilitating tax evasion by U.S. taxpayers.*" (DOJ130103: 1, *emphasis added*)
	OA20	"[N]otwithstanding the U.S. Government's undeniable awareness of this activity... prior to the UBS case, (...) [*p]rosecution of Swiss banks for assisting in this conduct* was unprecedented." (WegelinReply: 22)
	OA21	The PSI pointed to Wegelin's purely Swiss presence: "Wegelin *did not maintain an office or branch in the United States* (...) *did not have offices outside Switzerland* (...) *offices only in Switzerland.*". (verified complaint against Wegelin 20130225: 3-8, *emphasis added*)
	OA22	"As of December 2010, Wegelin had *offices in 12 locations in Switzerland*, including its *headquarters in St. Gallen. Wegelin has never had any branches or offices located in the United States.*" (Exhibit A to the Plea Agreement, 20121203: 1, *emphasis added*)
	OA23	"Wegelin's crime was (...) largely *committed from overseas* [from Switzerland]" (DOJ130225, 2013, *emphasis added*)
	OA24	"WEGELIN had *no branches outside Switzerland* (...) As of in or about December 2010, WEGELIN had 12 branches in Switzerland..." (indictment Wegelin, 20130225: 1, *emphasis added*)
		**Presence of distractions**
		n.a.

UNPROSECUTED ORGANIZATIONS:

TERRITORIAL CATEGORIZATION SCHEMES

**COSMO-POLITANS**		**GOAL-BASED TERRITORIAL CATEGORIZATION SCHEME**
		**Emphasis of territorial categorization scheme: Foreign regulator's interests and goals**
		<u>Limited belief in the absolute primacy of the domestic regulator's sovereignty</u>
	OA25	[Bank4X] "never saw itself as the Swiss island and thought that was now the big protection for any messes." (Int.88)
	OA26	Bank4X: "Every lawyer who has worked for banking groups in this way has certainly had to issue a statement at one time or another… because banking secrecy, following the traditional domestic attitude, does not work at all in terms of reporting. So, you always have to find a solution there… you can't put up a wall." (Int. 88)
	OA27	Bank5X: "We have always looked at the facts above all." (Int. 68)
	OA28	Bank7X: "Even though we love our independence, our sovereignty and our neutrality, but there have always been moments in banking history that have called them into question. That is why we have always sought a reading that is not a naïve reading." (Int. 92)
	OA29	Bank7X: "I myself had a law professor ask me, "how many aircraft carriers does Switzerland have and how many aircraft carriers does the U.S. have?" If you answered that, you answered the question of sovereignty. We cannot assume that we have a sovereignty that protects us from everything." (Int. 92)
	OA30	Bank8X: "One must not only look at these things in a purely legal and principled way, of course, but above all also in a factual way... The power of the factual." (Int. 56)
		<u>Recognition of powerful foreign regulators and their goals and interests</u>
	OA31	Bank3X: "At the end of the day, it's about a power play … So, this history was certainly also there with us, that one felt: ... We are not independent of the U.S." (Int. 59)
	OA32	[Bank5X] "very cosmopolitan" [and] "always was very … careful about such foreign regulators as the U.S., even though the bank never had a foreign presence" (Int. 82)

OA33 Bank5X: “They all had a similar way of thinking.” (Int. 56)

OA34 Bank5X: “In [Bank53]'s case, … a greater openness to and also a greater respect of the legal situation in the US.” (Int. 89)

OA35 Bank5X: “A very international regulatory logic in the case of [Bank5X]. They have been very attuned to what is going on in countries like the U.S.” (Int. 89)

OA36 Bank6X: “There were a number of banks that have always been very sensitive about the Americans. [Bank6X] was obviously one of them. These people thought that Americans were more dangerous than others, that you cannot hide.” (Int. 89)

OA37 [Bank6X] “always had strong affinities to and respect of the US.” (Int. 83)

OA38 Bank7X: “It is true that U.S. extraterritoriality has annoyed us from time to time. We were very aware of that” (Int. 92)

OA39 Bank7X: “We live in an international world. We have to deal with realpolitik.” (Int. 92)

OA40 Bank7X: “The first mistake would have been to ignore other country laws. The second mistake would have been to think that you can run the business in a very local way in a homegrown jurisdiction with only homegrown actions. And that is a mistake because in our industry one requires openness with the rest of the world.” (Int. 67)

OA41 Bank7X: “For us, this surveillance of other countries, and especially of the U.S., has never been anything shocking or special” (Int. 92)

OA42 [Bank8X] “has always been much more open to the world and to what the U.S. was after than others. After UBS, it quickly saw where things were heading to.” (Int. 56)

OA43 Bank8X: “There were already precedents where the Americans had done this before. We were interested in that. We had looked at that. So that didn't surprise us so much anymore.” (Int. 56)

OA44 Bank 8X: At [Bank8X], “the U.S. was always an issue that we closely followed.” (Int. 68)

OA45 “It is about responsiveness to what is happening in the outside regulatory world.” (Int. 82)

OA46 “They were more attentive to these developments in the U.S., more sensitive, more open, and also better able to absorb them.” (Int. 81)

OA47 “Any of these banks who are part of an international group face the reality of a globalized world. And they are aware of the strong incentive of US law enforcement authorities to have an extraterritorial application of US law.” (Int. 82)

OA48 “They had a different type of openness to regulatory issues in the U.S. and better international sensitivity. They reacted more naturally and strongly to this extraterritoriality.” (Int. 86)

OA49 “They have an understanding that there are differences in the national legal systems. They take that into account, but not in terms of regulatory arbitrage. They understand that this may be difficult to sustain with countries like the U.S.” (Int. 88)

OA50 “We were used to this… understanding that you are not stand-alone in the regulatory world. You need to watch carefully what foreign regulators like the U.S. want.” (Int. 88)

OA51 “They have a networked way of thinking, no island thinking, across borders information about foreign regulators is put into connection.” (Int. 88)

Sources of territorial categorization schemes

International socialization, education, and experience

OA52 Bank3X: “These are internationally very well-connected people.” (Int. 82)

OA53 Bank5X: “It's a very small bank. But the people were very well-connected European bankers, and the families too. And so, they have a very different and very international attitude as well.” (Int. 82).

OA54 Bank5X: “There were family members in the ownership here with strong U.S. ties who then also reacted more sensitively there.” (Int. 83)

OA55 [Bank6X] “the management and the owner family all have a link to the US...” (Int. 82)

OA56 Bank8X: “Also from the owner family perspective, the bank has a strong affinity with the Americans, who had hosted parts of the family back in World War II.” (Int. 56)

OA57 “They had a different type of socialization, a different type of openness to international issues and better international sensitivity. They reacted faster and more strongly to this extraterritoriality.” (Int. 82)

A1) International education

OA58 Bank4X: “The owners went to [an elite US university].” (Int. 82)

OA59 Bank6X: “Again, there is a lot of American experience on the part of some of the partners. [Chairman of Bank6X] has studied in the United States of America. He goes there regularly.” (Int. 89)

OA60 Bank7X: “I studied at [an elite US university].” (Le Temps, 06/08/19)

OA61 “I [legal council of a cosmopolitan bank] was in [another European country], doing the LLM.”. (Int. 68)

OA62 “They have completed an education in the U.S. or they have some other personal, friendship and family connection.” (Int. 72)

OA63 “In Geneva, they send their kids to Harvard. And almost all of them had an international experience or worked in a trader in the US or something like this before becoming a managing partner of the bank.” (Int. 82)

OA64 “They had a different type of socialization … much more international.” (Int. 82)

OA65 "[Legal council of cosmopolitan bank] did an executive training in the U.S. at [an elite US university]." (DerBund, 09/10/11, emphasis added)

A2) International experience

OA66 Bank4X: “The owners are people who are by nature a very international family. They have spent many years abroad and in international trade.” (Int. 82)

OA67 Bank5X: “On the one hand, with [a member of the top management team and the legal council], we had people from major banks who were able to assess the international risks quite well. Not the same, of course, but perhaps they have followed or had to follow similar cases. Not now on US and Tax, but just other stories. And we also had other people with that kind of experience, that's our current CEO, he had just come from UBS. He was also extremely open-minded and also saw it as a risk.” (Int. 68)

OA68 Bank5X: “We all had international experience and experience in large international banks, we had the same mindset.”(Int. 68)

OA69 Bank5X: “We also had a lawyer with US experience on our board. And she also took a very close look at it and said we have to be very careful.” (Int. 68)

OA70 Bank5X: “It is, of course, the Swiss entity. The [foreign owner] does not always intervene, but the entity is managed by men who belong to the [foreign owner] who think like the [foreign owner].” (Int. 89)

OA71 Bank 6X: “People who have a very international background.” (Int. 82)

OA72 Bank7X: “I [CEO of Bank7X] live alternately in Switzerland, England and other European countries.” (Le Temps, 06/08/19) “Compliance manager careers in international and internationally oriented banks look very different from those in traditional, conservative private banks.... if someone once worked in Head Office Compliance in Frankfurt, a next function could be Compliance in London, or Compliance in Switzerland.” (Int. 65)

OA73 “I [Legal Council of cosmopolitan bank] had had contact there with about 25 lawyers from all over the world. That's when I realized for the first time, as a 28-year-old lawyer, how firmly the rest of the world is upset about Switzerland... and said: How is it possible that you are still allowed to make such a mess? Because it's perfectly clear, legally, that one day you're going to pay for this." ... And all these, let's say, insights into how the others think. We also had Americans and Canadians and so on. Of course, this Switzerland-oriented bank management typically lacked that. They were not international or internationally-minded people. They had a focus on Switzerland and on practice. And that was it.” (Int. 68)

OA74 “These are generally people who had worked in a large international company. They may also have had U.S. experience” (Int. 72)

OA75 “The general manager of many of these banks typically is someone that has been sent by the head office. So, they are not a typical Swiss banker. They are typically outside bankers, foreign nationals. And so, there's an enhanced perception of the risk, especially at that point in time, where Switzerland was already on the radar screen of those countries, to avoid situations that could be embarrassing.” (Int. 70)

OA76 “Even for small Swiss banks with no foreign activity. It's fair to say that somebody who has had more of an international experience, has been more confronted with the US in the past, would be more sensitive to this and to the concrete risk for the bank.” (Int. 82)

OA77 “we find elite bankers whose careers are also very international—but neither boundaryless, nor very qualified. Similar to those in the first fraction, these bankers have spent long periods abroad, often in one of the large international banks and across several countries. However, even when they had an international career, they did so by traveling within the same firm and by remaining within the banking sector.” (Araujo, 2020: 107)

OA78 “...we find top managers with careers that are very international, academic and non-bounded… the boundaryless and global banker, educated at the most prestigious business schools. Not only do they possess a Top MBA and have they studied abroad, they might also be foreign citizens and have worked repeatedly and for longer spells in the major financial centers..." (Araujo, 2020: 106)

A3) Experience with regulatory cross-border issues

OA79 Bank3X: “One of our US clients, this was an elderly man and he was late with his tax return. It was all transparent, all disclosed, but he was late. And then the IRS approached us and said the client is not compliant. And then we had to withhold taxes and transfer them directly to the IRS. And the Americans are very old-fashioned about that. You need to do that by cheque. You can't do a wire transfer. And at some point, one of those cheques disappeared, didn't arrive. And at the same time you have to report and say "We have now sent a cheque in the amount of XYZ US dollars." And then the IRS said the money didn't come and if we didn't pay within 30 days, then they would freeze all the assets of the bank in the US. And that's fatal, of course, because at that time we still had a custody account in the U.S. and several hundred million assets of our customers were in the U.S... That was already such a wake-up call at that time.” (Int. 75)

OA80 Bank3X: “In 2003 or so, we had the legal case with [a foreign company and a foreign regulator]. .... So, this experience, of course, has shaped us... And, because of this case, we have increased compliance and the position of compliance everywhere quite a bit.... with us, afterwards, you just heard the grass perhaps grow. We were paranoid because of the experience and the impression. There one got simply clarity, we are not definitely sovereign here in Switzerland. We can be attacked and flattened there.” (Int. 59)

OA81 [Bank4X] “We had a scandal in Jersey. Anything that had to do with offshore, that's where we were touchy afterwards.” (Int. 88)

OA82 Bank7X: “The first blow to Bank76 was, of course, Madoff. The owner then took 500 million out of his own pocket to compensate many customers and get out of there. Of course, this led to a change in the owner family's risk awareness. Virtually the entire management team was replaced. As a result of the Madoff case, special attention was also paid at UBS.” (Int. 65)

OA83 Bank8X: “In the mid 2000s, [Bank8X] faced sanctions-related enforcement in the US and paid more than USD 500m for a deferred prosecution agreement.” (Int. 82)

OA84 Bank8X: “If you look at the internal documents, you'll see that everything after that [experience with a foreign regulator] actually goes on the defensive. The attitude was generally, "We can't have this happen to us again."” (Int. 79)

OA85 Bank8X: “All the directives were then revised. The compliance department was strengthened."

OA86 “If you have worked in a large bank, you have a better understanding of what the DOJ is capable of and how brutal they can be in the end. International banks have all had more or less big crashes with other regulators at some point. Then it's part of the business to deal with these things. And a lot of the other banks, they've never faced considerations on these kinds of issues, or have never been anywhere on the radar.” (Int. 54)

OA87 “These are players who are used to this, of course, who have witnessed and lived through X procedures in X countries-.” (Int. 54)

OA88 Legal, I was already in Legal back then.” (Int. 79)

OA89 “Some of the banks who had good behavior, it's because of their own history. Whether they had an issue with foreign regulators. Whether some had been hit.” (Int. 82)

OA90 “The French banks had experiences with extraterritorial US regulation before. Some have had sanctions issues.” (Int. 82)

OA91 “International banks have had to deal with foreign regulators and other authorities all the time. Maybe not to the extent and in the way, but still. So especially for international banks. They were more used to that.” (Int. 83)

OA92 “International banks have had recurring legal problems with the United States.” (Int. 86)

OA93 “[Two banks] had an international regulatory issue shortly before, both in the U.S. and in Germany. Therefore, the banks were on guard” (Int. 81)

Internationally-open advice networks

OA94 “Networks play a role, and so do associations. Banks are herd animals. And then they talk about it in the Bankers Association. And you talk about it in the […] Association and, and, and. And then there's the […] Association. That has enormous power. This Group Think is enormously prevalent.” (Int. 83)

A1) Geneva private banking network

OA95 Bank7X: “Yes, we are not a very big bank. All the institutions in Geneva are much smaller compared to the big banks, but we are very internationally minded.” (Int. 92)

OA96 “Geneva has always been particularly open to international developments.” (Int. 70)

OA97 “French-speaking Switzerland seems to have always had a much greater affinity for the international.” (Int. 79)

OA98 “That the Geneva financial center was slightly more conservative, a little bit more careful in what they have been doing.” (Int. 82)

OA99 “These banks are all part of the old Geneva families. They have known each other for centuries.” (Int. 82)

OA100 “Those banks they always had an international sensitivity. And the Groupement des Banquiers Privés, they have been basically working on the end of banking secrecy and the impact for banks for 20 years.” (Int. 82)

OA101 “Originally, the Geneva private bankers they were all cousins.” (Int. 82)

OA102 “They were exchanging information more and doing things together. They had their own Association, which was called, Groupement des Banquiers Privés. They had their own expert also for US questions in there, too.” (Int. 82)

OA103 “They have some far family connection. You can find some routes between the private bankers, and it's a group of families who were more or less owning Geneva at the time and still do.” (Int. 82).

OA104 “[Head of a cosmopolitan Swiss bank from Geneva], you would see him as one of the top bankers in Switzerland. He's close to everyone. Those people they know each other.” (Int. 82)

OA105 “Geneva has always been open to the world.” (Int. 82)

OA106 “They were talking among themselves all the time. I know that. There's a real cluster there.” (Int. 83)

OA107 “What is true is that there is sharing. We are an ecosystem. That is, if I have a problem with a population of customers and I run into someone in charge - one of the other banks, I say, "Watch out, there's a problem."” (Int. 92)

OA108 “What is certain is that in our Geneva financial center, there is a continuous exchange and will... We cannot afford to have too different views.” (Int. 92)

A2) Banks networked in the Association of Foreign Banks

OA109 “That network there, the professional network among compliance professionals, was very pronounced.” (Int. 86)

A3) Networks among similar-minded private banks

OA110 Bank3X: “The banks that we've been in active exchange with, that we've judged to be reliable and trustworthy, have actually all gone in that direction as well, that they've said, "We're paying attention in America."” (Int. 75)

A4) Networks among cosmopolitan cantonal banks

OA111 “Of course, we have a good relationship with the Bank3X. The CEOs had a good exchange of views at the time: how do you see it or how do you go about it?” (Int. 59)

OA112 “Bank4X, Bank3X, Bank5X and Bank3X, that's a group like that, they also coordinated a lot among themselves back then. That is also a real network.” (Int. 54)

OA113 “Bank3X, and Bank4X and Bank3X, they cooperated quite a bit. Bank8X as well.” (Int. 85)

<u>Self-selection between internationally oriented banks and legal advisors</u>

OA114 Bank5X: “Bank8X and then UBS, close contacts, exchange with different people, not only from the legal department, also from the highest management.” (Int. 68)

OA115 Bank74: “We had contacts. You could exchange ideas. We had a connection with lawyers in Washington, New York.” (Int. 92)

OA116 “They have a much stronger exchange already with other people who are looking not only at Swiss law but also very much at U.S. law.” (Int. 65)

OA117 “They get at least part of the American legal culture, and they get it directly.” (Int. 81)

OA118 “The closer the Group was to the U.S. market, the sooner it understood what was at stake and also took appropriate action.” (Int. 81)

OA119 “There is then also a kind of self-selection or certain different connections between internationally oriented banks and their lawyers and rather the domestically oriented banks and their lawyers. They get completely different lawyers and advisors than the others.” (Int. 88)

LOCALS **ATTRIBUTE-BASED TERRITORIAL CATEGORIZATION SCHEME**

Emphasis of territorial categorization scheme: The principles of territoriality and sovereignty

<u>Strong belief in the principles of territoriality and sovereignty</u>

OA120 Bank9X: “A strong belief in the territoriality of Swiss law, so that basic idea that Swiss law applies on Swiss territory, on Swiss grounds. No other regulator can just come and displace it.” (Int. 87).

OA121 Bank9X: “The territorial principle was absolutely anchored. Here it was difficult to imagine that American law would be invoked. And vice versa, it would also be difficult to imagine Swiss law being enforced in the USA. Basically, one assumes that if one does not operate in this territory, one is not subject to the other laws, which, by the way, one does not know or knows very little about.” (Int. 91)

OA122 “We are on Swiss soil, Swiss law applies here. If another country, be it America or somewhere else, has a problem, then they go through requests for legal assistance, through the Swiss Federal Council. And the Swiss Federal Council will never in its life allow any extraterritorial power to implement its own law on Swiss soil. So this trust was absolute.” (Int. 64)

OA123 “One has relied on it: If they want something, they will come via mutual legal assistance. Then it would go via a request for legal assistance from the American DOJ to the Swiss Department of Justice. And then it would normally be judged there and then either rejected or forwarded. And that's it.“ (Int. 64)

OA124 “It was like a culture. A belief, as deep as that they believed that they have Swiss nationality. That they are beyond the reach of a foreign authorities.” (Int. 70)

OA125 “No information was going to be made available to the countries of residence of their clients, because, as you know, there was no administrative assistance available until late in the game.” (Int. 70)

OA126 “It was said that if it was a mere tax evasion, then banking secrecy would never be lifted. And one felt very comfortable in this sphere and very, very, very safe. And it was for a long time. And that's an important point. Legally, this is actually a very safe thing. It was impossible to open tax-evaded funds, tax-evaded accounts through a letter rogatory.” (Int. 71)

OA127 “This principle of territoriality of Swiss law is the basic norm, that each country, each state is responsible for its own country in the final analysis, as far as these legal applications are concerned. We had absolutely relied on that.” (Int. 77)

<u>Little recognition of foreign regulators and their goals and interests</u>

OA128 Bank1X: “And the focus at that time was only on the greater area [of a particular Swiss city].” (Int. 84)

OA129 Bank7X: “They never really thought beyond the borders of [a particular Swiss canton].” (Int. 83)

OA130 Bank7x: “Because, of course, you were not very sensitive to problems coming from abroad.” (Int. 89)

OA131 Bank8x: “That's really very much related to themselves, so in the canton.” (Int. 85)

OA132 Bank9x: “We are responsible for the region in which we are based. And that is our orientation.” (Int. 77)

OA133	“There was a long-term attitude by some to play the Swiss card. But in our business, which is thriving through openness to the world, it's difficult to operationalize and maintain.” (Int. 67)
OA134	“In Switzerland, in central Switzerland, there was the mindset. "Foreign countries are far away, and we are in Switzerland.” (Int. 74)
OA135	“Those banks and bankers, they were socialized in Swiss law and kind of a more inner Swiss perspective.” (Int. 82)
OA136	“The affinity to international compliance topics in general was not developed at all.” (Int. 83)
OA137	“In many banks, there was such an insular way of thinking. We are here in safe Switzerland. Period.“ (Int. 88)
OA138	“The protective wall, Switzerland as a protective wall, and yes, that was so the traditional private banker thinking.” (Int. 88)
OA139	“That kind of thinking is none of our business. We are in safe Switzerland.” (Int. 88)
OA140	“A bank that said, "I don't care about foreign countries, I'm only in Switzerland."” (Int. 89)
	<u>*Focus on domestic regulator and domestic laws*</u>
OA141	Bank1X: “The whole focus was only on compliance with Swiss law. This whole cross-border issue, it didn't play any role at that time.” (Int. 84)
OA142	Bank1X: “Our compliance department was not focused on taking care of US law.” (Int. 91)
OA143	Bank3X & Bank6X: “They just thought we were a pure Swiss retail bank. It's none of our business. That was the thinking.” (Int. 83)
OA144	Bank7X: “We never did anything forbidden. That was the real problem. We only ever did what was allowed under Swiss law.” (Int. 89)
OA145	Bank7X: “Basically, a lower openness to foreign laws.... Also a low international legal culture.” (Int. 89)
OA146	Bank7X: “This kind of Swiss territoriality, sovereignty, Swiss law full stop. Or perhaps even less, perhaps just [a specific Swiss canton].” (Int. 82)
OA147	Bank95: “That was not an issue. We took care of the fulfillment of Swiss law and what FINMA had instructed us to do. “That was to be fulfilled. Period. That's how we ran our business.... Swiss legislation, banking law and FINMA. Those were our two gods. (laughs). We had foreign customers, but foreign law didn't interest us, of course.” (Int. 77)
OA148	“This is Switzerland, there is Swiss jurisdiction. The foreigners, they come here, here we can serve them according to Swiss law.” (Int. 53)
OA149	“The single biggest mistake that private banks have done was to think that you could run a sustainable business model, as long as you were complying with your home jurisdictions laws in total ignorance for the jurisdictions of where your clients were residing.” (Int. 67)
OA150	“In the 2000 years and before, there was this view that, provided you were really following the Swiss legal framework closely, you could then pretty much do whatever you wanted with no regard for the other jurisdiction. And that basically was a mistake.” (Int. 67)
OA151	“The idea that you also have to observe other legal systems in Switzerland, that didn't even occur to them.” (Int. 75)
OA152	“The fact that they had international clients, they did not realize that this would create an international dimension to them. For them they were clients of a Swiss bank in Switzerland.” (Int. 82)
OA153	“We had quite clearly had the position. We go by Swiss law, and Swiss law is so and so”. (Int. 86)
OA154	“They didn't think too much about it. If, then everything goes according to Swiss law.” (Int. 86)
OA155	“All of these cases were arbitrage logics. There were actually none that wanted to break the [Swiss] law. It was all a matter of arbitrage.” (Int. 89)
OA156	“FINMA is also the international lead regulator for the supervision of... Swiss banks internationally (lead regulator), so that it not only supervises banking activities in Switzerland, but also those abroad.” (GPK, 2010: 3247)
OA157	“Switzerland places a strong emphasis on the principle of home country control… the principle of home country control is solidly engrained in the Swiss legal and regulatory framework. As a result, the Swiss approach to cross-border banking services is a fairly liberal one.” (DuPasquier & Fischer, 2010: 3)
OA158	"From a Swiss perspective, the regulation of cross-border banking activities on the domestic market hinges upon a «physical presence» test.” (DuPasquier & Fischer, 2010: 3)
OA159	"...the registration of a branch in the relevant Swiss (cantonal) Trade Register formalizes the ratione loci jurisdiction of the FINMA over the activities conducted by that entity." (DuPasquier & Fischer, 2010: 4)
OA160	"The Swiss legislator has decided in favour of applying the incorporation theory. ...companies are subject to the law of the state under whose regulations they are organised if they comply with the publicity or registration requirements of that law or, if no such requirements exist, if they have organised themselves in accordance with the law of that state. The domicile theory can also be applied in Switzerland, but only on a subsidiary basis." (Wyss & Zulauf, 2001: 136)

Sources of territorial categorization schemes

Domestic education and experience

OA161 "Trust in Swiss law taught at Swiss universities." (Int. 64)

OA162 "So that trust has been had." (Int. 64)

OA163 "These are experiences that have become ingrained in our DNA. When we had law lectures, the theme was always, we are Switzerland, we are leading, we have our own legal rules, we are proud of the way we solve our coexistence legally. And we have our neutrality and sovereignty, which means nobody from the outside has anything to say. I think this is a bit of a Swiss DNA problem. You also noticed then, many banks with foreign executives reacted differently. They just didn't have this basic trust in Swiss jurisdiction." (Int. 64)

OA164 "It has a lot to do with the culture of the Swiss. We grew up there in a country with a high degree of legal security. And we relied on the fact that what is legally established in Switzerland is not only valid, but that you can also refer to it and that it will be protected by a court in the event of a conflict. We were all one hundred percent convinced of that." (Int. 64)

OA165 "This strong belief that you are a Swiss bank and you operate exclusively under Swiss law, even though you have these international clients. I think that is somehow historically based. It's always been done that way. And what you always do is good. At that time, it was not yet trendy to look at the cross-border aspects." (Int. 78)

OA166 "When we examine the number of firms in which top managers have worked during their trajectories, we see that careers of about one quarter of the top bankers evolve within a single firm… more than 60 percent have always stayed within on e country... the banking sector is still more "Swiss" than the economy as a whole." (Araujo, 2020: 98-99)

OA167 "...we find exclusively national careers: these bankers tend to be Swiss, they have been abroad either for very short periods or not at all and they have rather strong links to the political and administrative spheres in Switzerland. They have studied mainly at Swiss universities." (Araujo, 2020: 101)

OA168 Managers with a "fairly national orientation. They have rarely spent longer periods abroad and possess no international education. . . , they also tend to accumulate mandates in the Swiss political field." (Araujo, 2020: 107)

Socialization in banks from other tax heavens

OA169 "Well, the banks [from another secrecy jurisdiction], they were actually like hard core Swiss banks, with Swiss mindset." (Int. 54)

OA170 "In [another secrecy jurisdiction], we had absolutely the same. So, the head office lawyers were always saying, "Yeah, secrecy prevails, so we should not worry." We have had the same thinking. Don't worry." (Int. 55)

OA171 "I mean, [another secrecy jursidiction] is one of the European countries that held on to banking secrecy for a very long time because it also played an important role in its economy. And that had a big impact on the attitude of the banks and on the reactions to international developments." (Int. 81)

OA172 "Of course, [another secrecy jurisdiction] was also relatively strong in secrecy protection and so on for private banking. Similar to the traditional Swiss banks." (Int. 83)

Domestically oriented advice networks

OA173 "Many of these banks were organized in the Swiss [private banking] association." (Int. 53)

OA174 "The association of [domestic banks] and its sub-associations in which members convened and exchanged information regularly." (Int. 54)

Self-selection between domestically oriented banks and legal advisors

OA175 Bank0X: "Our compliance consultant did not deal with the intricacies of U.S. law. Rather, he focused on Swiss law. What works and what doesn't work in Swiss law." (Int. 62)

OA176 "Small banks outsourced compliance function to external compliance consultants with exclusive focus on Swiss law." (Int. 71)

OA177 "We are Swiss lawyers and know only Swiss law." (Compliance Consultant of many local Swiss banks) (Int. 71)

OA178 "We in the legal department also had the whole set of instruments ready to support this protective wall thinking. Difference tax fraud, tax evasion, where you do not have to provide legal assistance and so." (Int. 88)

**EPISODE1**		**UNPROSECUTED ORGANIZATIONS:**<br>**APPLICATION VS. ADAPTATION OF TERRITORIAL CATEGORIZATION SCHEMES**
**COSMO-POLITANS**		**Application of goal-based territorial categorization scheme**
		Alertness and thorough attention to enforcement event
	OA179	Bank4X: “That was really a pure compulsory exercise.” (Int. 88)
	OA180	Bank4X: “I'm trying to say that this was not necessarily the wake-up call. But that was a normal action, that one said, well, so now we look at that.” (Int. 88)
	OA181	Bank5X: “I think that was really due to the fact that we had also spent a lot of time there. Not only me, but also my colleagues, even on board level, and our lawyer, to really become aware of these risks.” (Int. 68)
	OA182	Bank7X: “That's where the recognition comes from. The recognition that it is raining outside comes from the UBS case.” (Int. 92)
	OA183	Bank7X: “Once we were able to capture information [from the UBS case], the goal was to manage our risk as best we could. If, and especially, of course, if we know or learn or understand that there is a problem with a bank, whether it's UBS or another one, we have to do mitigation risk and make adjustments.” (Int. 92)
	OA184	Bank7X: “Then it's the attention you want to give to the situation. Either you try to understand, or you bury your head in the sand. We, since we already had an American sensibility, that was important for us. From then on, when the UBS case came up and especially when the DPA was published, it was clear that as soon as you have something that is public, you read it and study it carefully. Otherwise, what would we have a legal department for?” (Int. 92)
		Situating goals of U.S. regulator within broader international political developments
	OA185	Bank3X: “But we have also noticed that the whole banking secrecy will at some point no longer hold. Also with regard to these requirements on the part of the EU, which have actually become loudly visible relatively simultaneously...” (Int. 75).
	OA186	Bank4X: “The derogatory statements by [the German ministers] Theo Weigel and Peer Steinbrück [about Swiss banking] in 2008. The fact that there was a completely different mood overall, that the business model is not a business model, you just couldn't overlook that and then pull back and say, "as long as I have my feet on Swiss soil here, everything is fine.” (Int. 66)
	OA187	Bank4X: “There was a different mood that was perceived, it was not conceivable that you would violate laws of other countries, so overall this orientation or this interpretation, this greater perception of these international dynamics, seems to have been more present here than perhaps in other banks.” (Int. 66)
	OA188	“Switzerland has not had such good press in the world for a while. Of course, the pressure that the U.S. has built up, Germany, France, other countries, that's been hotly debated in leading media-, Wall Street Journal, FT and so on. And these newspapers, of course, are also read by the boards of directors. And that simply led to the fact that then the managements said to themselves, risk-return is no longer right.” (Int. 65)
	OA189	“Also France made this very strong statement at that time about tax compliance. And we took the view that it was better to deal with this issue now. ... We thought we need to quickly deal with this and put an end to what could become a very problematic situation.” (Int. 70)
	OA190	“It was also felt within the EU that at some point this would no longer be tolerated.” (Int. 75)
	OA191	“We said we'd rather adjust to a new world early on than stay in an old world that can't and won’t stay that way anyway.” (Int. 75)
		Goal-based territorial categorization
		A) Focus on conspiracy / U.S. person / tax issue
	OA192	Bank3X: “It is about tax law and that of course starts with US person and not the residence.” (Int. 59)
	OA193	Bank4X: “It has been seen, tax evasion, black money of Americans is prohibited.” (Int. 88)
	OA194	Bank5X: “... That is, any tax liable [U.S.] person.” (Int. 68)
	OA195	Bank65: “In or about spring 2008, Bank65 learned that the IRS was investigating UBS AG for tax-related criminal offenses related to UBS assisting its U.S. taxpayer clients in holding undeclared accounts at UBS in Switzerland....” (SOF65)
	OA196	Bank7X: “The whole UBS affair is not an SEC affair at all.... It's really a tax case. That was the big challenge.” (Int. 92)
	OA197	Bank80: “On February 26, 2009, following the UBS Senate hearings, Bank80’s Management Committee met again to discuss the issues affecting U.S. taxpayer-clients.” (SOF80)
	OA198	UBS 2008: “After learning this information, ... The document circulating the new policy stated that "US authorities" were taking "a tougher stance on tax avoidance and on breaches of investment regulations."” (SOF95)

OA199 Bank8X: “You've seen that you ... drive into this conspiracy story.” (Int. 79)

OA200 Bank9X: “For us, the focus has been on the tax issue. For us, SEC has actually been practically no issue.” (Int. 73)

OA201 Bank9X: “When the main act takes place in the USA… that has been the connecting point where I have said, there I understand that the Americans function and think like that.” (Int. 73)

OA202 Bank9X: “Then it rushed when the question arose, what is aiding and abetting? What is conspiracy? It suddenly happened so quickly, so turbulently, that it was not possible to form, how shall I say, a doctrinal opinion. It was not possible to form a consolidated doctrinal opinion based on in-depth analyses, but in fact one can say that the Americans have imposed their judgment and also have their interpretation until the end, have not deviated from it.” (Int. 73)

OA203 Bank9X: “The idea was that the intention of the U.S. regulator was not only to catch and punish the banks directly, but to close the way from U.S. customers to foreign banks. That is, the goal is actually, and this was also seen in the agreement, to make life difficult for the untaxed Americans, in that no more banking services were available in Switzerland.” (Int. 73)

OA204 Bank9X: “The primacy of Swiss law has not been very important for us, because we have always looked at it from the perspective of aiding and abetting. And according to the motto, if the main offense, i.e. the non-declaration of taxes, takes place in the U.S. and one makes a contribution to the offense by supporting it, then it has actually been comprehensible for me, or for us it has not been, I don't say absurd or absolutely inconceivable, that the U.S. then links it and says that an offense or an aiding and abetting is considered to have been committed there, where the main offense was committed.” (Int. 73)

OA205 Bank9X: “In the UBS case, we were of the opinion that it was not relevant what subsidiaries or companies UBS had in the US, but what was relevant was what the private wealth managers discussed together with the US taxpayer, what they did together, and at most even whether they intentionally supported him.” (Int. 73)

OA206 Bank9X: we have immediately taken up the aspect of aiding and abetting in this respect… We thought pretty quickly that the main act was the non-declaration of the assets to the IRS, that is a U.S. act. And the aiding and abetting, whether that's account maintenance, hold mail and so on, that facilitated that, the non-declaration, that's already been an act in Switzerland.” (Int. 73)

OA207 “A number of actors, ... already when the UBS case became public in the spring 2008, became more aware of the tax risk that was existing in addition to the regulatory risk. ... and so you will find a number of institutions that in 2008, 2009 started cleaning their books and asking for evidence of tax compliance of their clients.” (Int. 70)

OA208 “The situation is alarming. I have never before lived a comparably difficult situation in my 30-year-long banking career. The extraterritorial thinking of the Americans, the claim to prescribe business to sovereign countries and companies (...) is particularly tricky in the cross-border business.” (Interviewee; DieWeltwoche31/07/08, emphasis added)

OA209 “All banks will learn to live with the stronger prescriptions also [banks] in Switzerland.” (Interviewee; Sonntagszeitung24/08/08, emphasis added)

LOCALS **Application of attribute-based territorial categorization scheme**

Limited attention

OA210 Bank3X: “We were not really paying attention and not realizing that we had to.” (Int. 82)

OA211 Bank4X: “The USA did not interest us much.” (Int. 86)

OA212 Bank9X: “USA was a non-issue at the time.” (Int. 77)

OA213 “You just do your business, you’ve always done it that way. One is sure that this is how Swiss banking works. After all, it did. For decades. ...customers hidden here in Switzerland, untaxed, and they didn't see any problem either. These are misjudgments, maybe even missing judgments more than misjudgments.” (Int. 54)

OA214 “We experienced it all very far away because we have no activities in the U.S. at all, we have no travel at all.” (Int. 91)

Belief in Swiss territoriality and sovereignty remains undisrupted

OA215 Bank1X: “This extraterritorial reach was not yet so much in the foreground at that time. One has just primarily seen these *license aspects* and also thought, well, that is the UBS.” (Int. 84, *emphasis added*)

OA216 Bank1X: “That this UBS case was primarily seen as a case where *this very territoriality of Swiss law* is observed, that one just thought, well, if we are on US soil and travel there and actively acquire customers, do marketing, as perhaps UBS did, ... but if we don't do that now, but concentrate on Swiss soil, then Swiss law must still apply. And another regulator can't just come in and say, I'm now enforcing my law over Swiss law.” (Int. 84, *emphasis added*)

OA217 Bank6X: “I was duped, or rather, they were very strongly attached to *the territorial idea*.” (Int. 85, *emphasis added*)

OA218 Bank9X: “There was just the belief we're in Switzerland, and we’re under Swiss law.” (Int. 87)

OA219 Bank9X: “We were actually not yet aware of this conspiracy aspect, this aspect of aiding and abetting, which was actually already present in the UBS case. (Int. 77)

OA220 “[P]eople used this as an argument that it is a question of whether you go to the U.S., whether you send an e-mail to the U.S. or accept calls from your clients who are in the U.S. – whether you have a U.S. nexus – which triggers this jurisdictional element.” (Int. 5)

OA221 “Switzerland is a *sovereign constitutional state*”. (Interviewee; Reuters13/03/09, *emphasis added*)

OA222 “We have to internalize that we do not break the *laws of a country* in which we are *active.*” (Interviewee; Sonntagszeitung08/03/09, *emphasis added*)

OA223 “Advisors to customers often *travel abroad to* visit their clients, but traveling asset managers can quickly come into *conflict with foreign laws.*” (Interviewee; Handelszeitung12/08/09, *emphasis added*)

OA224 “*In Switzerland, basically Swiss jurisprudence applies.*” (Interviewee; Finanz&Wirtschaft03/10/09, *emphasis added*)

OA225 “Even if the American business was only entirely passive, I will *not go to America.*” (Interviewee; DerBund31/10/09, *emphasis added*)

OA226 “If one day *Parliament* finds that tax evasion is to be criminalized, then we will have to accept it.” (Interviewee; DerBund11/03/08, *emphasis added*)

OA227 Swiss “banking secrecy is *established in the Constitution* and it does not seem to me that it is in danger.” (Interviewee; Finanz&Wirtschaft01/03/08, *emphasis added*)

OA228 “[E]very *country makes its own laws.”* (Interviewee; Sonntagszeitung09/03/08, *emphasis added*)

OA229 “...in Switzerland usual procedural rights and legal actions remain guaranteed.” (Interviewee, TagesAnzeiger18/11/09, *emphasis added*)

OA230 “I do not believe that *our legal system* is called into question in the case of UBS.” (Interviewee; Finanz&Wirtschaft03/07/09, *emphasis added*)

OA231 “There are views in Switzerland that requests by foreign states [for Swiss banks’ client information] must *respect the Swiss 'sovereignty'* over its tax laws - the '*tax sovereignty*'.” (Interviewee; NZZamSonntag01/06/08, *emphasis added*)

OA232 “[W]e do not tolerate *any state interfering* in this private matter.” (Interviewee; Die Weltwoche05/11/09, *emphasis added*)

OA233 The interviewee points “to the *mutual respect for foreign legal orders* and to the *sovereignty of foreign states,* which the U.S. courts need to consider when requesting Swiss banks' client data.” (Interviewee; TagesAnzeiger2/05/09, *emphasis added*)

OA234 “The decisive factor is that Switzerland continues to apply *the Law of Switzerland and not that of the foreign state to (...) Swiss banks.*” (DerBund11/03/08, *emphasis added*)

OA235 “Banking secrecy is a purely Swiss matter*... no foreign state has any influence on it.*” (Interviewee; Weltwoche14/08/08, *emphasis added*)

OA236 “For 50 years, *Swiss banks* have lived by the following rule: We strictly uphold Swiss legislation while ignoring foreign legal norms.” (Interviewee, Swissinfo30/08/12, *emphasis added*)

OA237 “There was *this element of territoriality* and saying that you are only active in Switzerland if you offer services that are correct under Swiss law.” (Int. 89, *emphasis added*)

OA238 An interviewee stated in the aftermath: “*We overestimated the effectiveness of Swiss territoriality.*” (Interviewee; HandelsZeitung02/04/13, *emphasis added*)

OA239 “*Swiss ground, US ground*, that was the thinking for a long time. But the USA has a rather unique tax law in the sense that every American, every US citizen or green card holder is taxable, no matter where he lives. And that’s why this argumentation of Swiss banks was not valid in the first place.” (Int. 83, *emphasis added*)

<u>*Exclusive focus on Swiss law*</u>

OA240 “The belief was that U.S. tax law does not apply on Swiss soil... there was a false security.” (Int. 56)

OA241 “At the time of the UBS event, it was still like this: We comply with these laws, according to Swiss principles in Switzerland, because we are Swiss. And then that’s okay.” (Compliance Consultant of several local Swiss banks) (Int. 71)

OA242 “The traditional Swiss private banking business with the tax, and we wouldn’t know anyway if [the client assets] were taxed or not. If they’re not taxed, it’s just tax evasion. And from a Swiss perspective, we didn’t care… we looked at this UBS case with the glasses of Swiss law - just not with the US legal glasses, but *with the Swiss legal glasses*.” (Compliance Consultant of several local Swiss banks) (Int. 71, *emphasis added*)

OA243 “We looked at that legalistically and imagined what exactly is prohibited from *the point of view of Swiss law*.” (Compliance Consultant of several local Swiss banks) (Int. 71, *emphasis added*)

OA244 “...we all rightly said, we are not subject to U.S. jurisdiction.” (Compliance Consultant of several local Swiss banks) (Int. 71)

OA245 “The fact that we have a Swiss legal system that was ancient, I mean, very well-rehearsed. And the U.S. regulators had no validity in Switzerland, the Americans had no jurisdiction in Switzerland. And so on.“ (Compliance Consultant of several local Swiss banks) (Int. 71)

OA246 “Foreign authorities need to respect the course of law.” (Interviewee; TagesAnzeiger13/05/09, *emphasis added*)

OA247 “The solution found *complies without limitations with Swiss law*. This is something very important for the financial center Switzerland, whose foreign customers rely heavily on the *predictability of the Swiss legal system.”* (Interviewee; Reuters19/08/09, *emphasis added*)

OA248 “This differentiation [between tax evasion and tax fraud] is *our legal position*. *We Swiss people... We Swiss people* would be well advised, *if we did not let us impose the legal order.*” (Interviewee; Sonntagszeitung24/08/08, *emphasis added*)

OA249 “We had this idea [that the Swiss banks were only supervised by Swiss law and precisely not by the U.S. authorities] until very recently. The entire industry and not only in relation to the problem of the U.S.”. (Int. 2)

OA250 *"Swiss-based banks*, considered that they *were only bound by Swiss law*." They followed the "restrictive position that the Swiss authorities have taken with respect to the applicability of foreign law led to the *belief that, in effect, Swiss law was the only relevant law to these institutions that were only present in Switzerland, and that foreign law had no applicability*." (Int. 5, *emphasis added*)

OA251 Switzerland has "a *democratically legitimized understanding of the law."* (Interviewee; HandelsZeitung12/03/08, *emphasis added*)

OA252 "Wegelin and other Swiss banks believed that *Swiss-only* banks passively accepting US clients (...) would remain below the radar screen of US prosecutors. Wegelin's business model was typically based on the *absence of any presence on US territory*; the *lack of aggressive marketing* in view of targeting undeclared US clients; *travel bans* on bank employees to the US; a *prohibition of direct communications (by email, fax, phone, letters or others) with US resident clients*; and a broad use of *hold mail* to retain banking documentation *in the bank's Swiss offices."* (Troller et al., 2013, *emphasis added*)

<u>*Attribute-based territorial categorization*</u>

A) Focus on SEC issue / US nexus

OA253 Bank0X: "It was interpreted according to the motto, this is UBS, which is actively doing market development, *in Florida*. They get on the plane and even do it actively from Switzerland. That is *on site* with the appropriate people, but not in this way." (Int. 62, *emphasis added*)

OA254 Bank0X: "One has primarily focused on these *licensing* aspects at the time." (Int. 74, *emphasis added*)

OA255 Bank13 management believed that the U.S. regulator targeted "*the U.S. crossborder businesses at UBS*" and "significant differences" existed with a purely Swiss-focused bank. (SOF13, 2015, *emphasis added*)

OA256 Bank14: U.S. regulatory issues would not be faced by "*Swiss" banks*. (SOF14, 2015, *emphasis added*)

OA257 Bank14 interpreted that "[t]he exceptional case of UBS" was due to the UBS' business in the U.S. Similar regulatory issues would not be faced by banks that would focus on "*Switzerland" only*. (SOF14, 2015, *emphasis added*)

OA258 Bank1X: "One looked primarily at these *licensing aspects* and at this residency status." (Int. 84, *emphasis added*)

OA259 Bank20 "wrongly believed that it could accept and service U.S. account holders who it knew or had reason to believe were engaged in tax evasion so long as (...) account holders (...) held accounts nominally structured in the name of a *non-U.S. based legal entity."* (SOF20, 2015, *emphasis added*)

OA260 Bank26 believed that banks that did not "give *any advice abroad*" would not fall under U.S. jurisdiction. The U.S. jurisdiction was triggered by the bank's use of U.S. jurisdictional means, such as: "*cross-border advice, whether by phone or email*", "*travel outside of Switzerland to solicit business*", "*maintain a U.S. desk* or market its services *in the United States* or to U.S. taxpayers", "*wire-transferred assets"*, "*e-banking services*". In turn, undeclared accounts of U.S. clients could be accepted "only if the customer physically opened the account *in Switzerland*" or if "U.S. resident clients (...) designate a representative with power of attorney *in Switzerland* and (...) specify shipping instructions *outside of the U.S.*". (SOF26, 2015, *emphasis added*)

OA261 Bank9X: "Today we understand better that you, even if you sit here, you need to comply with laws of other countries as well. But then, the whole industry here did not work like that. We were thinking, we're doing banking here in Switzerland. Only later, things started to change. But at the time, in terms of our bank, we saw it more as a UBS issue because they were caught because *they did it there*." (Int. 87, *emphasis added*)

OA262 "It was mixed up because that had nothing to do with the tax issue, but that's *just the SEC issue when they don't travel*." (Int. 56, *emphasis added*)

OA263 "We take the UBS case, look at it, say that we don't have that, ... that's a special case. This is UBS, which was also over there and had *violated the SEC rules*. We didn't do all that." (Int. 71, *emphasis added*)

OA264 "...the common belief after the UBS case among Swiss bankers that *local banks without a presence in the US would not be within the reach of US authorities*. What had sealed the fate of UBS - according to the common belief - was the toxic *combination of on- and offshore business with US clients, which had made the bank liable to prosecution in America*." (Troller et al., 2013, *emphasis added*)

OA265 "[I]t is not the same thing, particularly, *if one is a bank with activities on the ground, then one has dispute matters on the ground* (...) what UBS *did in the United States* was forbidden - the extremely active solicitation of customers *there."* (Interviewee 2, *emphasis added*)

OA266 "[T]he violation was that UBS had solicited [clients] *on U.S. territory*. It solicited the clients or prospects *on U.S. territory* for moneys that were held illegally abroad. And, U.S. regulations did not allow that. So, there was a clear infringement. (...) [UBS] actively solicited clients *on U.S. territory....* the general impression was, this is a case of UBS bank or global banks in general, that do cross-border banking, but essentially *have a presence on U.S. territory*. (...) I think the UBS case demonstrates that all banks, who had solicited U.S. clients *in the U.S.*, had violated the cross-border rules, and potentially licensing rules, if those same banks *had a U.S. presence.*" The interviewee stated that Swiss banks *underestimated "the extraterritorial reach of the U.S. authorities*." (Interviewee 11, *emphasis added*)

OA267 "[T]he public perception in Switzerland was that UBS was targeted specifically for its pervasive interactions with current and potential U.S. clients *on U.S. soil*." (WegelinReply: 23, *emphasis added*)

OA268 "UBS became liable [under U.S. laws and regulations], because it did not strictly implement its *onshore* strategy in the U.S., and it *simultaneously was active offshore*." (Interviewee; HandelsZeitung25/02/09, *emphasis added*)

OA269 "Bankers know exactly that the client correspondence is deposited in the bank and *crossborder contacts via mail, fax or phone* should be avoided." (Interviewee; Bilanz27/02/09, *emphasis added*)

	OA270	"The bank [UBS] committed a sin *in the U.S.* (...) their system has been violated." (Interviewee; BernerZeitung05/03/09, *emphasis added*)
	OA271	"It is certain: Times, in which client advisors could *travel abroad without constraints*, are over." There "is an increasing number of governmental requirements, which criminalize the conventional offshore business model. For example, the U.S.A.: If a bank wants to serve American clients, *it has to be registered there*. Otherwise, it is prohibited to serve American taxpayers *on U.S. soil*. It *cannot call from Switzerland or send emails*. This constitutes a big restriction on crossborder business." (Interviewee, NZZam Sonntag29/03/09, *emphasis added*)
	OA272	[A] "bank must be registered with the U.S. authorities. Only then, it is allowed to serve American taxpayers *on U.S.* soil.... Or to contact American tax-liable clients by *phone or email.*" (Interviewee; NZZamSonntag29/03/09, *emphasis added*)
		B) Focus on active/passive distinction
	OA273	Bank4X: "We did *not actively reach out to US customers*." (Int. 86, *emphasis added*)
	OA274	Bank6X: "It was said at the time, … we are not *actively* involved. That concerns us less... We are not active, we are not international, territoriality principle, Swiss sovereignty and so on." (Int. 83, *emphasis added*)
	OA275	Bank9X: "The way it was seen in the bank, was at the time, it was an issue of UBS. We don't do that *we don't actively pursue American clients*." (Int. 87, *emphasis added*)
	OA276	"[O]ne also looked at it primarily with this *active, passive distinction*, which was also very dominant in the industry." (Int. 71, *emphasis added*)
		C) Focus on US resident clients/ other US clients
	OA277	Bank1X: "We just thought, those US citizens with residence in Switzerland, they do not fall under it. Instead, it is those who *live in the USA* who are problematic. That's why the measures we took in 2008-2009 were primarily aimed at customers living in the USA." (Int. 84, *emphasis added*)
	OA278	"[Bank4X] has focused in particular on *US clients domiciled in the US*. (Int. 74, *emphasis added*)
	OA279	Bank5X: "In the beginning, you had thought, there are the big differences: Are people *domiciled in the U.S.* or are they just U.S. citizens?" (Int. 57, *emphasis added*)
	OA280	"The perspective of Swiss banks as regards the US risk, was US residents, *people living in the US*. This is what was initially identified." (Int. 70, *emphasis added*)
**EPISODE2**		**UNPROSECUTED ORGANIZATIONS:**
		**APPLICATION VS. ADAPTATION OF TERRITORIAL CATEGORIZATION SCHEMES**
**LOCALS**		**Adaptation of territorial categorization scheme**
		**(From attribute-based to goal-based territorial categorization)**
		<u>*Disruption of belief in territoriality and Swiss sovereignty*</u>
	OA281	Bank0X: "The big bang, the big slap in the face was then in January 2012 with Wegelin. That's when everyone woke up." (Int. 62)
	OA282	Bank1X: "We are *suddenly confronted with a legal system that is not ours*, that we are not used to and that we honestly don't understand much about." (Int. 91, *emphasis added*)
	OA283	Bank1X: "I would say that it was a shock at first when it was published because we could not imagine that we could be worried about this *extraterritoriality* as conceived by the United States. It seemed quite unimaginable. That was astonishment to begin with." (Int. 91, *emphasis added*)
	OA284	Bank58: "We were only reminded by the Wegelin enforcement that, 'Oops, we might have set a bet on the wrong horse and we might have misunderstood things." (Int. 60)
	OA285	"That was then *the power of the factual*. And then the views can no longer diverge." (Int. 56, *emphasis added*)
	OA286	"After the Wegelin case, Swiss banks realized: Stop. The U.S. regulator can very well take action, even *extraterritorially*." (Int. 65, *emphasis added*)
	OA287	"The Wegelin case, that had already been fatal... that's important. And that applies to all these five banks that I'm talking about here. They reacted in roughly the same way. One would have otherwise let it run still relatively long, as it was, if the case had not come." (Compliance Consultant of several local Swiss banks) (Int. 71)
	OA288	"For a lot of Swiss banks that were more domestically based and heavily involved in cross-border business, without having to travel and without having affiliation abroad, this was really a total wake up call." (Int. 88)
	OA289	"Yes, we, bankers, cannot ignore that *a paradigm shift* takes place. (...) The big geostrategic changes cannot be ignored. We have to adapt, whether we want it or not." (Interviewee; Bank85; HandelsZeitung10/02/10, *emphasis added*)
	OA290	"The principle of territoriality has lost in significance." (Interviewee; SchweizerMonat02/13)

OA291 In the aftermath, the interviewee acknowledged that "*the territoriality of the law plays a diminishing role*, international standards - whoever decrees or enforces them - abound. I could not imagine such a development [back with the UBS case], but it eventually confirmed [with the Wegelin case]. (...) It concerns different legal doctrines. And the U.S. enforced theirs." (Interviewee, StGallerTagblatt08/10/13, *emphasis added*)

OA292 "The Bank Wegelin went down. The Bank Frey gave up. *Local banks don't have any other choice than to defer to the U.S.A."*. (HandelsZeitung24/10/13*, emphasis added)*

<u>*Goal-based territorial categorization*</u>

A) Focus on US persons / conspiracy / etc.

OA293 Bank3 "(...) under indictment in the United States for *conspiracy to defraud the United States*." (SOF3, 2015, *emphasis added*)

OA294 Bank14: "The bank observed how "[o]n February 2, 2012, the U.S. Department of Justice announced the indictment of Wegelin *for conspiring with U.S. persons to* hide more than $1 billion in financial account assets from the IRS. (...) small banks come into contact with U.S. authorities as well." (SOF14, 2015, *emphasis added)*

OA295 Bank1X: "The fact that one does not only focus on US resident customers, but rather on *this broader definition of US persons*, that only came in 2011-2012." (Int. 84, *emphasis added*)

OA296 Bank20: Swiss Banks were targeted that "*assisted undeclared U.S. taxpayers in evading U.S. income tax.*" (SOF20, 2015, *emphasis added*)

OA297 Bank77: "...under criminal investigation and later indicted in the United States for *conspiring with U.S. taxpayers to help them evade their U.S. tax obligation.*" (SOF77, 2015, *emphasis added*)

OA298 Bank79: "...indicted in the United States and charged with *conspiracy to defraud the United States by assisting U.S. taxpayers* in opening and maintaining undeclared accounts in Switzerland." (SOF79, 2015, *emphasis added*)

OA299 Bank9X: "The awareness for this *conspiracy aspect*, that came only in 2011 with Wegelin." (Int. 77, *emphasis added*)

OA300 "The whole *conspiracy* story, we all then actually became aware (Compliance Consultant of several local Swiss banks) (Int. 71, *emphasis added*)

OA301 Switzerland allowed "the United States to examine and handle additional requests for administrative assistance if they were based on a similar pattern of '*tax fraud and the like*' *as in the case of UBS.*" Thereby, Swiss regulators "would also hand over *information about banks that were never active on U.S. soil* and thus could not violate American law." (Interviewee, Sonntagszeitung30/01/11, *emphasis added*)

OA302 "The Wegelin case shows that times are over when banking secrecy could be sold to customers who wanted to evade taxes. (...) The Wegelin case illustrates as well that *those banks that are not located there have to comply with foreign laws.*" (Interviewee; NeueZürcherZeitung03/02/12, *emphasis added*)

OA303 "If they are honest, no Swiss bank with an ex-U.S. offshore business can admit that it would not become a target of the U.S. (...) All Swiss banks with *U.S. offshore clients* are in a catch-22 situation.

OA304 "(...) Naturally, we have to adhere to applicable Swiss laws." (HandelsZeitung10/03/11, *emphasis added*)

OA305 In 2008, "[m]ultiple executives of the U.S. administration already then told me into the face that I was a *criminal for the things that we then did in Cayman and also in Switzerland*. At some point, it would be our turn. The time has come ten years later." (DerBund09/10/11, *emphasis added*)

OA306 "U.S. charges Wegelin with *aiding tax fraud*". (Swissinfo03/02/12, *emphasis added*)

OA307 "[T]he U.S. Justice Department had indicted... Switzerland's oldest "pure" private bank" on charges of *enabling tax fraud*. (Meier et al., 2013, *emphasis added*)

OA308 The "*U.S. Justice Department had indicted* a *purely offshore bank* on charges of *enabling tax fraud* (...) Wegelin had *no U.S. branches,* which seemed to provide a firewall between it and U.S. tax authorities, but the U.S. Justice Department circumvented this problem." (Meier et al., 2013, *emphasis added*)

OA309 "[P]urely Swiss institutions have come under scrutiny with the Wegelin case on charges of *enabling tax fraud*." (Schelbert, 2014: 121, *emphasis added*)

UNPROSECUTED ORGANIZATIONS:

TERRITORIAL SELF-CATEGORIZATION

EPISODE1 **UNPROSECUTED ORGANIZATIONS:**

TERRITORIAL SELF-CATEGORIZATION

COSMO-POLITANS **Goal-based territorial self-categorization**

(Location <u>inside</u> the perceived territorial category of foreign regulator)

OA310	Bank12: "In the wake of UBS, beginning in 2008, Bank12's majority owner voluntarily implemented measures that it believed would stop *helping undeclared U.S. taxpayers evade U.S. taxes*." (SOF12, *emphasis added*)
OA311	Bank18: "In the wake of UBS, beginning in 2008, [Bank 18] began a comprehensive review of its policies and procedures." (SOF18)
OA312	Bank22: "In the ... September 16, 2008 memorandum, the then-head of Legal, Compliance, and Risk recommended that the Bank adopt modifications to its *practices regarding U.S. related accounts*." (SOF22, *emphasis added*)
OA313	[Bank22] needed "to *ensure that U.S. related customers complied with their U.S. tax obligations.*" (SOF22, 2015, *emphasis added*)
OA314	Bank3X: "So the idea that *you have to respect foreign law also in Switzerland*, that was a more recent development. As long as we abide by Swiss law, everything is fine - the UBS case has shown us that this is no longer the case." (Int. 75, *emphasis added*)
OA315	Bank3X: "*...that they have American customers*." (Interviewee; Bank3x; HandelsZeitung17/01/13, *emphasis added*)
OA316	Bank46: "In or around July 2008, ... Bank46 began a comprehensive review of its *business relationships with U.S. clients*. In or around April 2009, Bank46 started the process of identifying U.S. clients and examining the Bank's policies with respect to such clients." (SOF46, *emphasis added*)
OA317	Bank5X: "We really said, we have an exposure here." (Int. 68).
OA318	Bank52: "In or around February 2009, [the bank] created a task force to review the scope of its *business with U.S. persons*." (SOF52, *emphasis added*)
OA319	Bank5X: "Compared to the SEC story... we were more concerned about *the aid issue*, because it's just criminal in nature." (Int. 68, *emphasis added*)
OA320	Bank53: "In response to the UBS case in 2008, ...review of *existing U.S. accounts*." (SOF53, *emphasis added*)
OA321	Bank55: "The U.S. law obligations impinged on "*all U.S. persons*". The Bank's Board of Directors approved the more restrictive policy for *all U.S. persons* (U.S. residents and non-resident U.S. persons)." (SOF55, 2015, *emphasis added*)
OA322	Bank5X: "I think, in Switzerland, they didn't use that [active/passive distinction]." (Int. 78)
OA323	Bank62: "After announcement of the Department of Justice's February 18, 2009 deferred prosecution agreement with UBS... [the bank] began a thorough review of its business relationships with *U.S. taxpayers*." (SOF62, *emphasis added*)
OA324	Bank65: "As a result [of the UBS enforcement], Bank65 examined its handling of potentially *U.S.-related accounts*." (SOF65, *emphasis added*)
OA325	Bank65: "further assessed its *U.S. tax and reporting compliance requirements.*" (SOF65, 2015, *emphasis added*)
OA326	Bank69_1: "On July 31, 2008, Bank69/1 decided, in light of the UBS investigation by U.S. authorities, to take immediate measures to strengthen its compliance framework for *U.S. clients*." (SOF69, *emphasis added*)
OA327	Bank70: "In October 2008, Bank70 refined and enhanced its existing U.S. compliance framework by establishing a U.S. Competence Center to ensure that policies and procedures adequately addressed regulatory requirements." (SOF70)
OA328	Bank71: "[From] March 4, 2010, ... the Bank took measures to better ensure that its *United States clients* were or would become tax compliant." (SOF71, *emphasis added*)
OA329	Bank71 added "additional questions intended to assist in *identifying potential United States taxpayers*, who would then be obligated to comply with [Bank71's] more restrictive policies for United States accounts (...) implemented a new policy governing its dealings with *accounts affiliated with United States persons and entities with substantial United States ownership and/or beneficial interests*." (SOF71, 2015, *emphasis added*)
OA330	Bank7X: "We were never traveling to the US, and we were never developing specific US relationship with US persons in the US. ... but when the UBS case arrived, we had to ask ourselves whether there were *other jurisdictional issues* where we potentially could have been at risk." (Int. 67, *emphasis added*)
OA331	Bank76: "The Bank urged U.S. *taxpayers* who were customers of the Bank." (SOF76, 2015, *emphasis added*)
OA332	Bank8X: "have said, look, if they [UBS] over there have the problem, so do we. *Regardless of the license*. That was already relatively clear for us." (Int. 56, *emphasis added*)
OA333	Bank8X: "Now you can say, well, they also had the licensing problem, et cetera. That's probably what other banks, like Wegelin, thought: We are in Switzerland and don't have that problem. But we thought one step further and thought, okay, so that can't be the whole security." (Int. 56, *emphasis added*)
OA334	[Bank8X] "sought to increase its "understanding of *the legal situation in the U.S.A.*"" (Interviewee, SchweizerBank20/03/09, *emphasis added*).

OA335 Bank8X: "They said: '*US person*' in the broadest sense. ... one has just said, the English have said: 'You can't just take US residence, but there are many variations. It's *a tax issue*.'" (Int. 79, *emphasis added*)

OA336 Bank9X: "We then said relatively quickly, yes, we can't escape the exposure." (Int. 73)

OA337 Bank9X: "…suddenly became aware, you can't offer a service as a normal service if you don't ask the question at the same time, *can the normal service be abused to evade taxes*." (Int. 73, *emphasis added*)

OA338 Bank9X: "In our interpretation, the question was then strongly raised, is offering a banking service to an untaxed client *aiding and abetting*? In the end, it was *a conspiracy to defraud the IRS* together." (Int. 73, *emphasis added*)

OA339 Bank9X: "[W]e have the *risk of aiding and abetting* if we offer our very normal banking services without checking or clarifying whether a customer is taxed." (Int. 73, *emphasis added*)

OA340 Bank93: "In response to the UBS investigation, Bank93 began implementing a number of measures that ... imposed limitations on accounts held by *U.S. taxpayer-clients*." (SOF93, *emphasis added*)

OA341 "These banks started realizing that you can only take *U.S. tax-compliant clients*." (Interviewee 5, *emphasis added*)

LOCALS

Attribute-based territorial self-categorization

(Location <u>outside</u> the perceived territorial category of foreign regulator)

OA342 Bank0X: "It is only about Swiss law, *we do not actively put our foot on a foreign territory* to work the market." (Int. 62, *emphasis added*)

OA343 Bank0X: "They said, no, it's Swiss law and we focus on that. That had been the opinion and the thinking. And that's what one did then." (Int. 62)

OA344 Bank2: "management was aware that U.S. authorities were pursuing Swiss banks that facilitated tax evasion for U.S. accountholders in Switzerland. But [Bank'2] *management was not deterred by this action because the Bank had no U.S. presence*, and it was willing to take on U.S. accountholders that were being forced out of other Swiss banks." (SOF2, 2015, *emphasis added*)

OA345 Bank3 "...has never had a desk dedicated to the U.S. market or any private bankers or "relationship managers" responsible for soliciting U.S.-based clients." (SOF3, 2015)

OA346 Bank0X: "We did not feel affected at all in 2008-2009." (Int. 91)

OA347 [Bank13] "*organized under the laws of Switzerland*." (SOF13, 2015, *emphasis added*)

OA348 Bank13 "management believed that significant differences in the operation of the U.S. crossborder businesses at UBS and [Bank13] justified that decision. [Bank13] *never sent representatives to the United States* or advertised its financial services there." (SOF13, 2015, *emphasis added*)

OA349 Bank14 "is headquartered in the city and canton of Schaffhausen, Switzerland (...) and maintained a branch office (...) in the canton of Zurich. (...) [It kept] accounts *outside of the United States*." (SOF14, 2015, *emphasis added*).

OA350 Bank15 is "a private bank based in Bern, Switzerland. (...) It has *never had any branch offices outside Switzerland*. (...) The Bank *never established a U.S. presence nor held a U.S. desk."* (SOF15, 2015, *emphasis added*)

OA351 Bank1X: "And these U.S. dual citizens who have primarily Swiss residency. That means in the end, *as far as also these SEC license aspects are concerned, we are safe*, we are correct on the way." (Int. 84, *emphasis added*)

OA352 [Bank17] "had no procedures in place to require proof of U.S. tax compliance by U.S. clients when opening an account." (SOF17, 2015)

OA353 Bank 17 is "a *licensed Swiss bank."* (SOF17, 2015, *emphasis added*)

OA354 [Bank19] "established *under the Cantonal Act*." (SOF19, 2015, *emphasis added*)

OA355 Bank1X: "There was a form of perhaps a little naivety or at least ignorance of all this. Again, I would say this was supported by the fact that we never imagined that the U.S. laws could apply to *institutions operating in Switzerland and only in Switzerland*, because we also *did not make any commercial moves towards the United States*." (Int. 91, *emphasis added*)

OA356 Bank19 "did not have procedures in place that required proof of U.S. tax compliance at account opening." (SOF19, 2015)

OA357 Bank19 "never utilized a strategy to market its services to U.S. citizens or U.S. residents and did not specifically target U.S. Persons as potential clients. [The bank] *never had a U.S. desk or any foreign desk*." (SOF19, 2015, *emphasis added)*

OA358 Bank20 "has never had a U.S. desk or any other separate structure or organization for U.S. clients. [Bank20] never utilized a strategy to market its services to U.S. citizens. (...) the Bank had *no organized group seeking business beyond the borders of Switzerland*. (...) predominantly attracts customers through *walk-ins at its physical offices within the canton, some local sponsoring events, and local advertisements*. (...) *No one* from the bank, relationship managers or otherwise, has *ever traveled to the United States* to either solicit clients or to provide clients with investment advice." (SOF20, 2015, *emphasis added*)

OA359 Bank21 "focuses on providing retail banking services to *local residents*, mortgages for properties in *the region* (...) provides banking services not only to local residents, but to individuals who visit or have vacation residences located *in the region*." (SOF21, 2015, *emphasis added*)

OA360 Bank23 "organized *under the laws of Switzerland*." (SOF23, 2015, *emphasis added*)

OA361 Bank26 considered "as a *local bank under Swiss law* we do not give any advice abroad, neither by e-mail nor by phone." (SOF26, 2015, *emphasis added*)

OA362 Bank27: "*never had a presence in the United States* and its relationship managers *never traveled to the United States* to seek or service U.S. customers. (...) main office in Lucerne. It also has a small office *in Zurich*. (...) principal market is high net worth individuals, families, and family-owned companies in the *German-speaking part of Europe* (primarily Switzerland and Germany). Approximately 80 percent of [Bank27's] customers are *located in Switzerland*, and most of the remaining customers are *located in Germany* (...) [Bank27's] position was that it could assist U.S. account holders that it knew or had reason to believe were engaged in tax evasion so long as (...) the account was *nominally structured in the name of a non-U.S. based entity.*" (SOF27, 2015, *emphasis added*)

OA363 Bank28 "did not structure, operate, or supervise its U.S. Related Accounts in any way that was different or separate from its non-U.S. Related Accounts." (SOF28, 2015)

OA364 Bank28 "had an informal policy to accept clients only if they had a link with the Canton (...) and not to pursue and/or solicit *clients outside of Switzerland*. (...) never *had a U.S. desk or any foreign desk*." (SOF28, 2015, *emphasis added*)

OA365 Bank29: "Prior to November 2013, [Bank29] did not require any confirmation or proof that the beneficial owners of its U.S. Related Accounts were in compliance with their U.S. tax obligations." (SOF29, 2015)

OA366 Bank29 "had rules regarding maintaining bank accounts that focused on *compliance with Swiss [law yet, it did not consider] the impact of U.S. criminal law* (...) [the bank] did not require any confirmation or proof that the beneficial owners of its U.S. Related Accounts were *in compliance with their U.S. tax obligations."* (SOF29, 2015, *emphasis added).*

OA367 Bank30 "has never had offices, branches, or subsidiaries *outside of Switzerland* (...) [Bank30] has never had a strategy to target U.S. persons or market its services *in the United States* or to U.S. persons. The Bank has *never had a U.S. desk."* (SOF30, 2015, *emphasis added*)

OA368 Bank31: "we have *no branches in the US* (...) Until 2011, (...) the Bank had no specific strategy aimed at acquiring U.S. clients *nor did it engage in marketing efforts aimed toward U.S. clients*. Most of the Bank's U.S. clients were primarily citizens and residents of the United States who lived full or part time in the Ticino region (...) Until 2011, the Bank's account opening documentation and its policies and agreements were written only in Italian, and the Bank has not generally been equipped to service or attract non-Italian speakers (...) *no Bank employee ever traveled to the United States to solicit clients*, service clients, or provide clients with investment advice." (SOF31, 2015, *emphasis added*)

OA369 Bank32 "*place of business is the Swiss Canton of Aargau*... maintains its headquarters and twelve branches in the Swiss Canton of Aargau... *never* maintained branches *outside of* the Canton of Aargau." (SOF32, 2015, *emphasis added)*

OA370 Bank32 "did not send mail associated with some U.S. Related Accounts to the United States. More particularly, through its hold-mail service and at the request of clients (...) [Bank32] did not engage in soliciting clients located abroad and its employees *never traveled outside of Switzerland* for business purposes. [Bank32's] marketing and advertising were *limited to the Canton of Aargau* and consisted solely of German-language advertisements and marketing materials and private events for citizens of the Canton of Aargau, the local area served by [Bank32]. [Bank32's] population of U.S. Related Accounts arose out of the connections of the holders of U.S. Related Accounts to the Aargau area or to [Bank32]." (SOF32, 2015, *emphasis added*)

OA371 Bank34: "Although [Bank34] had U.S. Related Accounts over the Applicable Period, at no time did [Bank34] have an organized business unit or a business strategy to attract and service non-Swiss account holders (...)." (SOF34, 2015, *emphasis added*)

OA372 "No [Bank34] employee ever traveled outside of Switzerland for purposes of soliciting U.S. persons or servicing U.S. Related Accounts. (...) [Bank34] did not seek to have U.S. persons as clients. For example, [Bank34] did not engage in soliciting clients abroad. [Bank34's] non-institutional marketing and advertising were solely in German and French and *strictly limited to certain regions in Switzerland in* the form of advertisements, private events for Swiss citizens, and referrals from existing clients." (SOF34, 2015, *emphasis added*)

OA373 Bank38 "certainly not a big player on the American market, *does not process emails from the U.S. anymore,* and faxes and letters sent from there are no longer accepted. Phone calls from there remain unanswered." (Interviewee; Weltwoche13/08/09, *emphasis added*)

OA374 Bank38: "At [a] meeting of the Bank's executive board, one executive noted that (...) *as a Swiss bank* with a business strategy aimed at a Swiss customer base, we would not in any case be in the crosshairs for the U.S. authorities." (SOF38; 2015, *emphasis added*)

OA375 Bank38 "is a corporation organized *under the laws of Switzerland*." (SOF38, 2015, *emphasis added*)

OA376 Bank4X: "That if you try to reduce this nexus to the U.S., you remain in the end-, you see yourself as *a bank operating in Switzerland* and would like to be this-, to be *active in this Swiss jurisdiction* in the end... We are *domiciled in Switzerland* and *do not have a foreign branch* like the big banks, which are also represented there." (Int. 74, *emphasis added*)

OA377 Bank4X: "At the time, we thought: Well, we are different from the UBS case. We have not been so active on U.S. soil. But if we *concentrate on Swiss soil*, which means *no travel* to the U.S., *no communication* with the U.S., *no e-banking*, *no Internet connections*, then you could say that we fall under Swiss law. And there, what we do is not criminal, it's fine." (Int. 74, *emphasis added*)

OA378 Bank45 "does not have *any branches or subsidiaries located in the United States*." It "has local management, a local banking team, and a client base with *accounts held in Switzerland* that is distinct from, and tracked and managed separately from the [Bank45] operations in other jurisdictions." (SOF45, 2015, *emphasis added).*

OA379 Bank48's "business traditionally has been focused in and around [the Canton] and surrounding communities. [Bank48] *did not market its services in the United States*. It never maintained a desk dedicated to the U.S. market, assigned private bankers' responsibility for soliciting U.S. business, or encouraged private bankers to solicit U.S. business. [Bank48's] U.S. clients by and large had personal connections to the (...) region, including many who at one time resided, or had families who resided, in and around the (...) region." (SOF48, 2015)

OA380 Bank48 "did not pursue a plan or strategy to solicit business from the United States. It did not market its services in the United States, and did not allow client relationship managers to travel to the United States on business. (...) *banned written and telephonic communications with clients in the United States, and all e-banking contacts with U.S.-domiciled persons*." (SOF48, 2015, *emphasis added*)

OA381 Bank49 is a "Swiss private bank (...) *never had a U.S. desk or presence*, *never marketed to U.S. persons*, advertised *in the United States*, *nor traveled to the United States* to prospect for or service clients." (SOF49, 2015, *emphasis added)*

OA382 Bank54: "*...because of Swiss bank secrecy laws*, Switzerland would not freely exchange account information with the United States." (SOF54, 2015, *emphasis added)*

OA383 Bank5X: "We said after the UBS case, 'Well, *the territoriality principle is still intact* and that has not been crossed by the Americans. And what we did was fully compliant with Swiss law.'" (Int. 60, *emphasis added*)

OA384 Bank5X: "We have also never actively worked *in the USA*, already therefore, it is just not relevant for us." (Int. 57, *emphasis added*)

OA385 Bank59 "*Organized under the laws of Switzerland*." (SOF59, 2015, *emphasis added*)

OA386 Bank60 did not "ensure that all its clients with a U.S. nexus fully complied with their U.S. tax obligations." "...when deciding whether to accept U.S. persons as clients, [Bank60] applied the same procedures that it applied to all of its prospective clients. These procedures were *based on Swiss banking laws and regulations*, including know-your-client ("KYC") and anti-money laundering ("AML") rules." (SOF60, 2015, *emphasis added*)

OA387 Bank61 "…has 32 branches throughout Switzerland. It has never had offices, branches, or subsidiaries outside the country." (SOF61, 2015)

OA388 Bank63 "*never had operations in the United States*, and *never operated a U.S. desk*." "The bank maintains four offices in Switzerland to service clients in French-, German-, and Italian-speaking regions of the country. (...) all of the Bank's banking services are provided *by the Basel office*." (SOF63, 2015, *emphasis added*)

OA389 Bank64 "In accepting new clients, the Bank conducted diligence that *met the standards set by Swiss law and regulations* and complied with [Swiss law]. *As required by Swiss regulations,* the Bank identified on Forms A and T the beneficial owners of accounts opened in the name of legal entities." (SOF64, 2015, *emphasis added*)

OA390 Bank66: "At all times relevant to this matter, [Bank66] was organized *under the laws of Switzerland*." (SOF66, 2015, *emphasis added*)

OA391 Bank67 "[A] bank *under Swiss law*" ... "was required *by Swiss law* and government mandate." (SOF67, 2015, *emphasis added*)

OA392 Bank75: "With respect to U.S. customers, by *not sending bank statements and other mail relating to the accounts to the United States, documents reflecting the existence of the accounts remained outside the United States and generally beyond the reach of U.S. tax authorities*. With respect to all clients in the United States, the Bank mandated the use of *hold mail*." (...) In another email dated May 6, 2010, Credit Suisse Relationship Manager #2 employee contacted an employee of [Bank75] regarding the transfer of an account to the Bank: "I'm away during his stay. I have now ordered the gold so he can *take it physically* and can carry it 'over the road.'*"* (SOF75, 2015, *emphasis added*).

OA393 Bank75 "*…did not believe that it had an obligation to collect tax forms from U.S. account holders* (...)" (SOF75, 2015, *emphasis added*).

OA394 Bank7X "…is *subject only to Swiss banking regulation*". (Int. 75)

OA395 Bank78 "The actual situation *in the US* (UBS, Birkenfeld, etc.) has nothing to do with [Bank78] (...) [Bank78] *did not maintain a U.S. desk* or other *unit with a particular focus on U.S. clients*." (SOF78, 2015, *emphasis added*)

OA396 Bank79 "…chose to continue to service U.S. clients without disclosing their identity to the IRS and *without considering the impact of U.S. criminal law* on that decision. [Bank79's] view was that it could continue to accept and service U.S. account holders, even if it knew or had reason to believe they were engaged in tax evasion." (SOF79, 2015, *emphasis added*)

OA397 Bank79's "meeting minutes further state that "in the context of compliance, *all relevant [Swiss] regulatory rules* have to be strictly abided by *(inter alia CDB and AML)," referencing anti-money laundering and "know-your-client" regulations* (...)". There is no explicit mention in the March 9, 2009 board meeting minutes that *any discussion of the tax status of the U.S. customers took place*. (...) Following the March 2009 board meeting, a management board meeting was held on March 13, 2009, where the management board discussed opening accounts for U.S. taxpayers. Executive #1 noted that "*[b]ased on current law* the Board is giving us the green light to continue reviewing potential US clients (including ex-UBS) (...) in accordance with the *rules currently in force* (...) There is no explicit mention in the March 13, 2009 management board meeting minutes that *any discussion of the tax status of the U.S. customers took place*. (...) During the 2009 and 2010 years, the written agreements between [Bank79] and external asset managers made *no mention of U.S. tax compliance*. For example, an agreement between [Bank79] and an external asset management firm, EAM #1, signed July 30, 2009, confirms that EAM #1 has knowledge of *various Swiss laws*, including *antimoney-laundering laws,* and also contains an agreement that EAM #1 will not introduce to the Bank any clients whose assets originate from a crime or a criminal organization. However, there is *no mention of tax compliance."* (SOF79, 2015, *emphasis added*)

OA398 [Bank8X] "had *never (...) actively acquired or visited customers on U.S. territory. Neither, did it try to acquire customers via telephone or e-mail from Switzerland*." (Interviewee; BaslerZeitung14/09/11, *emphasis added*)

OA399 Bank8X: A "*Swiss"* bank committed to the *"compliance with the requirements of the Swiss legislature*." (Int. 18, *emphasis added*)

OA400 Bank8X: "Purely Swiss" [Bank8X] has "*no branches abroad"* ... "no offerings outside the jurisdiction of Swiss law." (Int. 18; *emphasis added*)

OA401 Bank89 "*had no branches or offices in the United States*, and because of its understanding that it acted in accordance with and not in violation of Swiss law, and that such conduct was common in the Swiss banking industry." (DOJ, 2013: 14, *emphasis added*)

OA402 Bank9X: “Because *we didn’t go to the US* to solicit clients. We did not advertise.” (Int. 87, *emphasis added*)

OA403 Bank9X: “We are not affected, we don’t have to work out our own position, except that we, yes, that this is none of our business.” (Int. 77)

OA404 Bank9X: “[P]eople simply said: No, that doesn’t affect us.” (Int. 77)

OA405 Bank9X: “Attitude: It doesn’t affect us, it’s UBS, they went too far, they were far too *active*, they were *on U.S. soil*, actively marketing themselves there, acquiring customers. We didn’t do all that.” (Int. 77, *emphasis added*)

OA406 “If you look at [the UBS case], there are particularly *active* facts described there that I have not encountered in this form… And therefore, of course, they saw themselves *outside the scope of the USA*.”

OA407 Swiss bankers “held the view that sales do not have to be registered [in the U.S.], insofar as the discretionary mandate was *issued outside the U.S.A.* and no regular contact existed with the client.” (GPK, 2010: 3245, *emphasis added*)

OA408 “We are not UBS. We are not comparable at all. We *don't operate in the United States*. And that was always the mantra that the banks held on to enormously. They thought that was the straw that broke the camel’s back, that they could distinguish themselves.” (Int. 83, *emphasis added*)

OA409 “...the belief that, in effect, Swiss law was the only relevant law to these institutions that were *only present in Switzerland*, and that foreign law had no applicability. (...) Mr [X] took the view that as [Bank8X] had *no offices* abroad, the only law they should abide by was *the law of Switzerland* (...) [Bank8X] took the position, '*We're in Switzerland. We are subject to Swiss law. We don't care about anything but Swiss law*. But this is basically the position taken by many banks [after the UBS case].” (Int. 5, *emphasis added*)

OA410 “We are a Swiss bank, which has to respect Swiss laws.” (Interviewee, SchweizerBank21/07/09).

OA411 “Most cantonal banks, with their client base, thought they were really not at risk, in the sense that *none of these banks have presence abroad*, *none of these banks were doing marketing abroad*.” (Int. 70, *emphasis added*)

OA412 “That was the misjudgment in part, that UBS was a special case, so to speak, that one was differently situated.” (Int. 72)

OA413 “The banks [from another secrecy jurisdiction], because we also come from a country with banking secrecy, thought the Americans couldn’t do anything to us. We didn’t do anything that was against either the *parent company law* or our *Swiss law*. And then we just continued to do that relatively blindly.” (Int. 86, *emphasis added*)

OA414 “We all said, "No, no, it’s our problem."” (Int. 87)

OA415 “[S]ome banks thought, ‘I am [Bank5x], or something like that, in Zurich. I just have *a presence in Zurich*. *Nothing else anywhere else*. I'll continue to do that.’ They opened their books to money that was coming out of UBS, etc., and then they ultimately got caught.” (Int. 11, *emphasis added*)

OA416 The interviewee “emphasizes that as a banker, he acts on *the territory of Swiss law*". (Interviewee; BaslerZeitung13/03/09, *emphasis added*)

OA417 The “bank operates its private banking *in Switzerland* under the *here valid rules*. This comprises also banking secrecy.” (Interviewee, TagesAnzeiger31/03/09, *emphasis added*)

OA418 The “*Swiss legal system counts* and one cannot release client data under pressure just like that.” (Interviewee; BaslerZeitung14/03/09, *emphasis added*)

OA419 “Like all bank advisers [that are registered] in Switzerland, he is obliged to examine the origin of the money entrusted to him. The question of *whether this is black money is not part of this examination*”. (Interviewee; BaslerZeitung13/03/09, *emphasis added*)

OA420 “They felt that they were really not at risk. At the time, as far as the US is concerned, the main risk that was identified was the *SEC risk*, and this was really what banks were focusing on. And they understood that by *being present in the US*, by *traveling to the US* or by *using Interstate Communication* means they could *run afore of SEC regulations*.” (Int. 70, *emphasis added*)

OA421 “And the type of bank that did not react after the UBS case did not see itself as an international player. Instead, this focus on the domestic market, even though they had customers from all over the world, fits in well. They saw themselves as *a Swiss bank*, which is simply not comparable with a UBS.” (Compliance Consultant of several local Swiss banks) (Int. 71, *emphasis added*)

OA422 “The one who said, what do we care, we are *here in Switzerland*.” (Int. 56, *emphasis added*)

OA423 “I think you just felt a little bit untouchable there.” (Int. 69)

OA424 “We don’t do *active crossborder business*, so we are not concerned [by U.S. rules].” (Int. 2, *emphasis added*)

OA425 “We are a Swiss bank, we *operate in Switzerland*, we have, *serve the Swiss market*. It does not affect us. We are *outside the reach of the U.S. regulator*. Maybe UBS is, because they were very active there, but we are at most *passive*. And then the UBS case happened and what was done, of course, was to look in great detail at these, let’s say, written channels that existed for the UBS case. And from these came pretty much exactly this conclusion that one said, of course, one must *not travel to the United States*, one must not make large demands, write and make phone calls to U.S. clients. But our banks didn’t do that anyway.” (Int. 71, *emphasis added*)

OA426 “And, of course, we investigated that a little bit more, what we had here and said, yes in all rule this is, if at all, a normal tax evasion and then we are on the safe side.” (Int. 71)

OA427 “We said very strongly that we are *not present in the U.S. at all*.” (Int. 61, *emphasis added*)

OA428 “With the consequence that people have, of course, falsely lulled themselves into a sense of security and said, yeah, like, we don’t have that. We have *little or no travel to the United States*.” (Int. 72, *emphasis added*)

OA429 “So, we are not doing that, we are very careful. We don’t have a *presence in the US*, we’re not *marketing clients*, we *don’t go to the US*. And so that was a general reaction of many in the Swiss banking industry.” (Int. 70, *emphasis added*)

OA430 “If you look at the Statement of Fact in the UBS DPA, you will see that there are certain actions in the foreground there, for example, *traveling to the U.S.* with diamonds in the toothpaste and things like that. And because of that, a lot of people have also been misled that you don’t fall under that at all.” (Int. 72, *emphasis added*)

EPISODE2 **UNPROSECUTED ORGANIZATIONS: TERRITORIAL SELF-CATEGORIZATION**

LOCALS **Goal-based territorial self-categorization**

(Location inside the perceived territorial category of foreign regulator)

OA431 Bank1: “*U.S. taxpayers without a Form W-9*” and “any new U.S. client account” were seen as a gateway for *U.S. exposure*. (SOF1, 2015, *emphasis added*)

OA432 Bank13: “...conducted a comprehensive review [in line with U.S. law].” (SOF13, 2015)

OA433 Bank14: “On July l, 2011, [Bank14] issued a formal directive concerning tax matters. The directive stated that, to *prevent the aiding and abetting of tax evasion, [Bank14] employees must not do anything that promotes or facilitates client breaches of their tax obligations or declarations or that conceals the existence of assets*. For example, the directive prohibited employees from delivering or collecting cash at a client’s domicile or assisting in the concealment of a beneficial owner.” (SOF14, 2015, *emphasis added*)

OA434 Bank17: “caused [Bank17] management to first consider the possibility that the Bank might be responsible for monitoring the tax compliance of non-Swiss clients *in other* jurisdictions... the *observance of the law and rule of the local country*.” (SOF17, 2015, *emphasis added*)

OA435 Bank20 “Effective July 2012, the Bank established a “Transnational Financial Services Business Policy,” which emphasized *controls that apply to almost all countries, including the United States.*” (SOF20, 2015, *emphasis added*)

OA436 Bank20 “*would be obligated to identify U.S. persons and share their information with the IRS.*” (SOF20, 2015, *emphasis added*)

OA437 Despite Bank21 “never had a U.S. desk or any other separate structure or organization for U.S. clients,” did not do “business outside Switzerland” and “did not encourage marketing outside Switzerland,” “the Bank would be *obligated to identify U.S. persons and share their information with the IRS*.” (SOF20, 2015, *emphasis added*)

OA438 Bank21 felt that banks would be subject to *“U.S. law”* with their *“U.S. person”* business. (SOF21, 2015, *emphasis added*)

OA439 Bank23 “has, among its clients, individuals and entities resident in Switzerland along with individuals and entities resident outside of Switzerland, including certain *clients who were or became citizens or residents of the United States.*” (SOF23, 2015, *emphasis added*)

OA440 Bank28 realized that U.S. clients led it to fall under U.S. tax laws (SOF28, 2015, *emphasis added*).

OA441 Bank28 recognized that “new clients domiciled outside of Switzerland sign a cross-border certificate (“Cross-Border Certificate”) attesting that the assets deposited at [Bank28] have been declared to *tax authorities in their home country.*” (SOF28, 2015, *emphasis added*)

OA442 Bank29 realized that U.S. legal claims encompassed “Swiss banks attendant to cross-border financial services”, which involved *“investment soliciting and/or advisory”* with respect to *“U.S. persons”*. Bank29 perceived to be under U.S. jurisdiction “*identifying the client as a U.S. person according to bank records.”* (SOF29, 2015, *emphasis added*)

OA443 Bank31 “recognized that ‘[w]e can no longer have clients who are U.S. persons who have not signed the W-9 form.’” (SOF31, 2015)

OA444 Bank41 identified “*U.S. clients domiciled in the United States.*” (SOF41, 2015, *emphasis added*)

OA445 Bank47 analyzed “its cross-border business with *U.S. Related Accounts*. To do so, [Bank42] has, among other things, conducted database searches and interviews with relationship managers and members of management, reviewed client dossiers, and analyzed relevant internal documents.” (SOF47, 2015, *emphasis added*)

OA446 Bank48 identified its “*U.S. clients*.” They were primarily “*U.S. accounts*” that generally arose out of the Bank’s domestic business.” (SOF48, 2015, *emphasis added*)

OA447 Bank54: “...revised its policies regarding *persons with U.S. tax obligations*.” (SOF54, 2015, *emphasis added*).

OA448 Bank64: “In March 2012, the Bank initiated a review of *U.S. client accounts* to identify any clients who had not filed a Form W-9 (or W-8BEN or W-81MY as appropriate).” (SOF64, 2015, *emphasis added*)

OA449 Bank66’s “relationships with *clients with residence in the United States*, and all *domiciliary companies with a U.S. person as the beneficial owner*.” (SOF66, 2015, *emphasis added*)

OA450 Bank75: “Also beginning in 2011, the Bank has required *U.S. customers* to execute an Acknowledgement of Regulatory Restrictions.” (SOF75, 2015, *emphasis added*)

OA451 Bank77: “...U.S. clients.” (SOF77, 2015)

OA452 Bank79: “On September 8, 2011, Executive # l told the management board that the *review of undeclared U.S. customers* was “well advanced” and continued “rigorously” (translated from German).” (SOF79, 2015, *emphasis added*)

OA453 “...that caused us [Swiss banks] to be brought *under American jurisdiction...* a solution that has caused us to *actually be directly under their supervision*.” (Int.2, *emphasis added*)

OA454 “So, they used withholding mail, no travel, a correspondent bank account at UBS... they really did everything possible to avoid a direct contact between themselves and U.S. territory... except that they held U.S.-resident clients”. After the Wegelin case, Swiss-

based banks started realizing "*the regulatory constraints that could apply in the jurisdictions of residence of their clients*". (Int. 5, *emphasis added*)

OA455 After the Wegelin case, it became clear that "*...this is the path through which they ultimately fall under U.S. law.*" (Int. 8, *emphasis added*)

OA456 I didn't have an employee who went to the U.S. to solicit funds. But if I was accepting funds from the U.S. via an external asset manager, I had to make damn sure that the external asset manager was himself compliant." (Int. 11)

OA457 "The cross-border business with private clients is changing tremendously. Times of tax arbitrage are over. An adaptation to a world *without fiscal banking secrecy* is necessary." (Interviewee, SchweizerBank21/04/10)

UNPROSECUTED ORGANIZATIONS:

PERCEPTION OF PROSECUTION THREAT

EPISODE1

COSMO-POLITANS

UNPROSECUTED ORGANIZATIONS:

PERCEPTION OF PROSECUTION THREAT

Perceived threat of organizational illegality

OA458 Bank4: "In mid to late 2008, in the wake of the UBS investigation, [Bank4] *began to assess the risks* of its own U.S. cross-border business. (...) [Bank4] sought to avoid ... risks that could arise from the unauthorized solicitation of U.S. clients" in relation to U.S. law. (SOF4, 2015, *emphasis added*)

OA459 Bank5: "this conduct *violated U.S. law.*" (SOF5, 2015, *emphasis added*)

OA460 Bank6 recognized the "heightened *risk that some U.S. account holders who had opened and maintained accounts at the bank were not complying with their U.S. income tax and reporting obligations.*" (SOF6, 2015, *emphasis added*)

OA461 Bank8 "knew that it was highly probable that some U.S. taxpayers who had opened and maintained accounts at the Bank *were not complying with their U.S. income tax and reporting obligations* (...) efforts were undertaken as part of a major initiative at [Bank8] intended to *augment compliance* across its private banking business through enhanced rules of conduct for businesses in (...) the United States." (SOF8, 2015, *emphasis added*)

OA462 Bank9: "In late December 2008, (...) [Bank9] informed External Asset Manager1 it would not accept additional U.S. clients *until the tax compliance status of the first 11 accounts was proven*. [Bank9] also advised the External Asset Manager that it could not engage in any activity associated with the management of the U.S. clients' accounts." (SOF9, 2015, *emphasis added*)

OA463 Bank10: "[I]n 2008, the Bank adopted a policy (the "2008 policy") that sought to avoid... risks that could arise from the unauthorized solicitation of U.S. clients in relation to U.S. securities laws." (SOF10, 2015)

OA464 Bank24 sought to enhance "the Bank's compliance with all applicable U.S. laws." (SOF24, 2015)

OA465 Bank24 "has, among other things, conducted email searches, reviewed client dossiers, and analyzed relevant internal documents. (...) Through its managers, employees and/or others, [Bank24] knew that some U.S. taxpayers who had opened and maintained accounts at the Bank *were not complying with their U.S. income tax and reporting obligations*" (SOF24, 2015, *emphasis added*).

OA466 Bank33: "Recognizing that certain *accounts had been opened under prior policies, including certain U.S. taxpayer accounts that might not have been disclosed to the IRS*, the Bank instituted a legacy account remediation project (...) those *account holders who were non-compliant as to any U.S. tax obligation.*" (SOF33, 2015, *emphasis added*)

OA467 Bank3X: "Of course, it depends on which risks a bank takes. For example, with American customers, the greatest caution was and is always recommended." (Interviewee; Bank3X; HandelsZeitung17/01/13)

OA468 Bank3x: "when the UBS issue came up, ... of course *we then looked very closely at our own business* and very quickly said: we certainly can't accept anything. Because if UBS now comes under pressure, it will certainly not end well if we were to say that U.S. clients are welcome here." (Int. 59, *emphasis added*)

OA469 Bank40 in 2010: "The Bank's Legal and Compliance Departments updated the Bank's Cross Border Manual for the United States and *prohibited various types of contacts with and services for U.S. clients.*" (SOF40, 2015, *emphasis added*)

OA470 Bank52: "In or around February 2009, [Bank52] created a task force to *review the scope of its business with U.S. persons*." (SOF52, 2015, *emphasis added*)

OA471 Bank62: "On February 27, 2009, [Bank62] adopted new policies restricting the opening of new accounts involving U.S. taxpayers." (SOF62, 2015)

OA472 Bank65: "By or about summer 2008, [Bank65] had *recognized the risks inherent in its U.S. client base* and reevaluated its U.S. client policies. Because of its *heightened awareness of U.S. tax. compliance risks, [Bank65] assessed and avoided taking on additional U.S. risks*. (...) The Bank alerted its relationship managers to *this potential compliance exposure item.*" (SOF65, 2015, *emphasis added*)

OA473 Bank68: "adopted a policy of encouraging *U.S. clients who had undeclared accounts* to declare those accounts to the IRS (...) identified one relationship manager who *violated* this policy by discouraging U.S. clients from declaring their accounts." (SOF68, 2015, *emphasis added*)

OA474 Bank70: "U.S. clients, some of whom did not comply with U.S. tax or FBAR obligations." (SOF70, 2015)

OA475 Bank74 “was aware that RM-1 may have *engaged in significant misconduct* with respect to the referral of U.S. Related Accounts to the Bank.” (SOF74, 2015, *emphasis added*)

OA476 Bank76 recognized that “U.S. clients that presented the greatest *risk of non-compliance with U.S. tax obligations.*” (SOF76, 2015, *emphasis added*)

OA477 “As regards the distinction between tax evasion and tax fraud, the interviewee replies: “*There is no comprehension for this distinction outside of Switzerland*. We have to find a solution.” (Interviewee; NeueZürcherZeitung15/02/09, *emphasis added*)

OA478 “[S]ome banks still rely too much onto the ‘depot’-type of business, which formerly has been the norm – the sole storage of money for people, for which it is *unclear as to whether they face tax problems abroad.*” (Interviewee; SchweizerBank20/11/08, *emphasis added*)

OA479 “May 2008, we have the announcement of the UBS investigation, then Spring 2009, we had the settlement. And at that time, banks started realizing that *there was an issue*, started looking into the SEC aspect and regularly also at the tax aspect. And some did it. Others just ignored it. (...) You have the banks that saw the UBS case and started taking action, and basically, I would say banks that reacted then, started really reacting in relation to the SEC aspect. They started realizing that going and prospecting clients in the U.S. *was a dangerous thing and that one should not do this*.” (Interviewee 5, *emphasis added*)

OA480 There are “risks with international payments”. (Interviewee; BaslerZeitung11/11/08)

OA481 “We are not interested in undeclared accounts from the U.S. (...) I cannot imagine that it would be the right strategy for Swiss banks, to have U.S. clients who do not declare their assets.” (Interviewee; Finanz&Wirtschaft12/01/09)

OA482 “We had clear rules. We exited the U.S.-crossborder business in 2008.” (TagesAnzeiger27/12/11)

Perceived threat of prosecution by the foreign regulator

OA483 Bank4X: “We perceived the U.S. regulator at that time to be *a consistent regulator*... as very rigorous… So, when *he threatens*, he doesn’t do it in the void.” (Int. 66, *emphasis added*)

OA484 Bank4X: “Honestly, I can’t imagine that you couldn’t *perceive [the threat]*. (laughs) I mean, I can’t look away from it. I actually think the other way around... On the contrary, I think you have to react to it. Period, end, closing time.” (Int. 66, *emphasis added*)

OA485 Bank5X: “We said: We are certainly *not flying under their radar*.” (Int. 68, *emphasis added*)

OA486 Bank5X: “After the UBS case ... we clearly thought: This story is *only just beginning*. In any case, they’ll be looking at who’s doing what in the financial center as a whole. So, that was already something we knew at the time.” (Int. 68, *emphasis added*)

OA487 Bank53: “Without any obligation to do so under Swiss law, the Bank, with a view to anticipating and *responding to potential regulatory requests as well as to properly manage its risk*, established and executed a series of increasingly strict and conservative policies and account reviews designed to reduce its existing population of U.S. Related Accounts and prevent the opening of new U.S. Related Accounts.” (SOF53, 2015, *emphasis added*)

OA488 Bank5X: “[T]hey’re *going to bash the Swiss subsidiary*… and ask them what they’re doing.” (Int. 89, *emphasis added*)

OA489 Bank5X: “Because Bank5X had such an exposure in the USA, it occurred to them earlier that they had to do something there… So, *vulnerable with that*… It’s easy for the regulator and the tax authorities to *build up pressure*. Whether that’s legal or not, whether it’s allowed or not, that doesn’t matter to the Americans.” (Int. 78, *emphasis added*)

OA490 Bank8X: “... if there is a problem, you have to address it. You can’t sit it out. *It will catch up with you*... There is nothing to sit out.” (Int. 56, *emphasis added*)

OA491 Bank8X: “We *don’t wait until they knock on our door*.” (Int. 56, *emphasis added*)

OA492 They “try not to break too much there [in the U.S.].” (Int. 81, *emphasis added*)

OA493 “Hitherto, every foreigner was able to come and say: 'Establish a foundation for me'. All banks did this. If we establish foundations at face value in the future, *we can be prosecuted by (...) foreign jurisprudence, as accomplices of tax evasion*. At the moment, I am having this point investigated within the bank.” (Interviewee; Weltwoche12/03/09, *emphasis added*)

OA494 Foreign authorities “have accelerated their pace against offshore banking practices. *New attacks are luring*.” (NeueZürcherZeitung15/02/09, *emphasis added*)

OA495 “My paramount priority at the moment is to *avoid that my employees are criminalized.”* (Interviewee; Weltwoche20/08/09, *emphasis added*)

OA496 *“They fear the risk that their employees could be arrested abroad and prosecuted criminally for abetting tax evasion*. UBS' problems in the U.S. started exactly like this.” (Interviewee; Weltwoche20/08/09, *emphasis added*)

OA497 Banks “recognized that they faced *a very real risk of prosecution akin to the case against UBS.*” (WegelinReply: 21, *emphasis added*)

UNPROSECUTED ORGANIZATIONS:

PERCEPTION OF PROSECUTION THREAT

Perceived threat of organizational illegality

OA498 Bank1: "However, in the wake of the Department of Justice's criminal investigation into UBS' U.S. cross-border business, Bank1 "began to assess the risks of its own U.S. cross-border business. (...) It considered the activities as *low risk*, as it hid behind the contractual relationships with EAMs." Bank1's "internal records reflected the identity of the U.S. clients associated with these accounts, in *compliance* with *Swiss law."* (SOF1, 2015, *emphasis added*)

OA499 Bank2 "[P]rior to 2012, [Bank2] *did not require any confirmation or proof that the beneficial owners of its U.S. Related accounts were in compliance with their U.S. tax obligations*. (...) [Bank2] *perceived value in Swiss bank secrecy* (...) chose to continue to service U.S. customers without disclosing their identity to the IRS and *without considering the impact of U.S. criminal law on that decision.*" Bank2 "made no effort to determine *whether such an entity was valid for U.S. tax purposes.*" (SOF2, 2015, *emphasis added*)

OA500 Bank3's "position was that it could actively assist U.S. clients that it knew or had reason to believe were engaged in tax evasion." (SOF3, 2015)

OA501 Bank3 "…performed no client due diligence concerning U.S. tax compliance... without considering *the impact of U.S. criminal law* on that decision." (...) Bank3 "made no effort to determine *whether such an entity was valid for U.S. tax purposes.*" (SOF3, 2015, *emphasis added*)

OA502 Bank7 "*did not violate* U.S. Securities and Exchange Commission ('SEC') rules." (SOF7, 2015, *emphasis added*)

OA503 Bank11 "*in compliance with Swiss law*." (SOF11, 2015, *emphasis added*)

OA504 Bank13: "These developments caused [Bank13] management to assess the Bank's U.S. cross-border business periodically throughout 2008 and 2009. (...) even after FINMA officials publicly addressed the legal risks of cross-border businesses by Swiss banks (...), [Bank13] management did not examine in detail the Bank's relationships with U.S. persons. At that time, the Bank deemed such an examination unnecessary given a *perceived low level of risk exposure for the Bank*." (SOF13, 2015, *emphasis added*)

OA505 Bank14 "ensured that its operations were *in compliance with 'Swiss law'*. For instance, 'Swiss law required EKS to identify the true beneficial owners of the entities on a document called a 'Form A''. (...) Bank Executive I 'added that he could imagine accepting new seriously high net worth customers from the USA at [Bank14] provided the *basic legal requirements are complied with* and provided there is an ongoing review' (...) the Bank would ensure that '*the FINMA conditions are fulfilled at all times*.' The risk of the Bank's U.S. cross-border business 'creating problems' was 'rather low'. The bank believed that it '*observes all U.S. regulations and conditions*.'" (SOF14, 2015, *emphasis added*)

OA506 Bank21: "In December 2008, (...) [t]he Bank knew, or had reason to know, that two of those accounts were undeclared. (...) the regulatory requirements for managing U.S. clients 'was stringent,' it decided that it would 'minimize risks' by pooling U.S. clients into the private banking division.' 'Likewise, in the Bank's 2009 training materials (...) explained to its employees (...) that it could accept and service U.S. account holders who it knew or had reason to believe were engaged in tax evasion.'" (SOF21, 2015)

OA507 Bank29 "opened and maintained undeclared accounts for some of these U.S. taxpayers *without considering that*, by doing so, *[Bank29] was helping these U.S. taxpayers violate their legal duties*." (SOF29; 2015, *emphasis added*)

OA508 Bank38's handbook stated: A "U.S. person has the option not to disclose to U.S. tax authorities." Bank38 "placed these account holders in a restricted category requiring additional monitoring by the Bank's compliance department. The Bank issued this directive after its auditor had approved it as *consistent*." (SOF38, 2015, *emphasis added*)

OA509 Bank44 implemented "more stringent measures to ensure that its clients *complied*." (SOF44, 2015, *emphasis added*)

OA510 Bank59 "was aware of the risk that certain U.S. clients might have been maintaining undeclared accounts at [Bank59] for the purposes of evading their U.S. tax obligations in violation of U.S. law. (...) As of February 19, 2010, then, [Bank59] *renounced its previous practice of accepting 'manifestly untaxed assets from foreign clients.'*" (SOF59, 2015, *emphasis added*)

OA511 Bank60: "In August 2008, (...) the Bank *continued to accept new U.S. clients, but only after review by the Bank's compliance department and approval by an executive board member.*" (SOF60, 2015, *emphasis added*)

OA512 Bank63: "*Under Swiss law*, an account holder *has the right to* withdraw his or her funds through any means including cash, gold, or physical securities, including bearer shares." (SOF63, 2015, *emphasis added*)

OA513 Bank72: "The bank implemented conditions that were intended to *prevent the Bank from 'falling into the trap of providing active assistance to tax evasion.'"* (SOF72, 2015, *emphasis added*)

OA514 Bank75: "Starting in or about June 2009, only employees specifically trained to handle U.S. customers are allowed to serve as relationship managers on U.S. Related Accounts to *ensure compliance with the appropriate rules*. The bank 'explained in an internal memorandum, *the existing regulations can be circumvented* by using such people [External Asset Managers].' (...) perceived to be '*compliant with U.S. law in* light of the issues then facing UBS. (...) had always *complied with the laws of Switzerland*.'" (SOF75, 2015)

OA515 Bank77: "[A]ccounts [were] *consistent with Swiss law* (...) Because Swiss law requires [Bank77] to identify the true beneficial owner of structures on a document called a Form A, it knew that these were U.S. client accounts. Nonetheless, for certain U.S. client accounts, [Bank77] private bankers and other employees aided and assisted some of these U.S. clients in concealing these assets and income from the IRS." (SOF77, 2015, *emphasis added*)

OA516 Bank78: "The Bank's anti-money laundering documentation was *in compliance with Swiss law*." (SOF78, 2015, *emphasis added*)

OA517 [Bank78] "*strictly complies with* [U.S. law] - and has always done so in the past." Bank78's "anti-money laundering documentation" was *in compliance with Swiss law.*" (SOF78, 2015, *emphasis added*)

OA518 Bank8X "was thus convinced that it was complying with Swiss law." (Troller et al., 2013)

OA519 "[T]he Swiss bank, more generally, practiced the *Swiss distinction between tax evasion and tax fraud*... Yes." (Int. 2, *emphasis added*)

OA520 "[T]he very firm belief in Switzerland that *tax evasion is not an offense*. I do remember (...) in like '93 or so (...) people were offering to open numbered accounts everywhere. That was quite normal. That was normal. And, yeah. As long as you do *nothing illegal, yeah, just optimizing your taxes was not a problem*. (...) I think a lot of the institutions thought that (...) that they were *within the law*." (Int. 10, *emphasis added*)

OA521 "As an important factor, it must be added that Switzerland (...) distinguishes between tax evasion and tax fraud." (Interviewee; BaslerZeitung07/03/08)

OA522 "I always clearly stated to my people back then: *You cannot engage in any criminal activity*. But, of course, we never asked foreign clients whether they correctly declared their accounts in their home country. It does not concern a bank. Only one thing we did not do during my time: We did not aggressively solicit clients, for instance, in the U.S.A., lay out the red carpet and say: Bring your assets to Switzerland, here, nobody asks for them." (Interviewee, BaslerZeitung18/03/09, *emphasis added*)

OA523 In Switzerland, "criminal acts have never and will never be covered." (Interviewee, HandelsZeitung18/03/09, *emphasis added)*

OA524 "We have a different regulation of criminal tax law. A *simple tax evasion is no reason not to accept money due to the double litigation principle*. (...) The *legalistic position is well-anchored*." (Interviewee; Weltwoche20/03/08, *emphasis added*)

OA525 "On the surface, we have no need for action due to the legal situation. (...) *I feel confident. The legalistic position is well-anchored*. (...) *A simple tax evasion is no reason not to accept money due to the double litigation principle coupled with banking secrecy*." (Interviewee; DieWeltwoche20/03/08, *emphasis added*)

OA526 "[W]e in Switzerland [distinguish] between tax fraud and tax evasion" (Interviewee; DieWeltwoche20/03/08)

OA527 "We bankers need to explain well to our customers that legal certainty and banking customer secrecy remain intact as privacy protection. (...) *There are several ways, within the legal and regulatory requirements,* which it is important to discuss with the customer." (Interviewee; HandelsZeitung09/09/09, *emphasis added*)

OA528 "You can come to us; we differentiate between tax evasion and tax fraud." (Interviewee; Der Bund31/10/09)

OA529 "We always *complied with* any applicable provisions." (Interviewee, HandelsZeitung10/03/11 *emphasis added*)

OA530 "At that time, we [at Bank81] *came to the conclusion that our existing practice of offering normal account services (...) continued to be justified*. So maybe we actually closed this door a little later than other banks." (Interviewee; BaslerZeitung14/09/11, *emphasis added*)

OA531 The bank "steadily *relied on the legal position* and *did not violate rules according to Swiss law.*" (Interviewee; Finews18/07/13; *emphasis added*)

OA532 In the past, we used practices that were *legal* according to Swiss law." (Interviewee, LuzernerZeitung04/09/13, *emphasis added*)

OA533 "Wegelin's explanation of its conduct [was] that it was acting in *accordance with Swiss law."* (DOJ130225: 14, *emphasis added*)

OA534 The interviewee stated that the bank was part of a group of banks that followed the rule: "We are a *legally-compliant* bank." (Int. 14, *emphasis added*)

Perceived threat of prosecution by the foreign regulator

OA535 Bank11 had "the knowledge that *Swiss banking secrecy laws would likely prevent the Bank from disclosing their clients' identities to the IRS*." (SOF11, 2015, *emphasis added*)

OA536 Bank14: "On February 11th 2009, a compliance consultant reported to the bank's board of directors that in the case of any 'misdoings, *the Bank would have to anticipate sanctions from the Swiss financial authorities.'* (...) Bank Executive #I also stated that '*there is practically no risk if U.S. customers travel to Switzerland and a customer account is handled locally*.'" (SOF14, 2015; *emphasis added*)

OA537 Bank19: "...reducing the chances that the U.S. government would learn the identities of the taxpayers." (SOF19, 2015)

OA538 Bank1X: "In 2008-2009 we still felt protected by Swiss law." (Int. 91)

OA539 Bank21's "board believed that the regulatory requirements for managing U.S. clients 'was stringent.'" (SOF21, 2015)

OA540 Bank21: "In the wake of UBS, in November 2008, [Bank21's] executive board discussed the issue of U.S. clients (...) [Bank21] was "receiving various inquiries whether we still accept new U.S. clients," and they discussed that they needed to be "aware that *the U.S. business also involves considerable risks*." (...) [the bank's board] decided that *it would 'minimize risks' by pooling U.S. clients into the private banking division and assigning them to one of three private banking relationship managers.*" (SOF21, 2015, *emphasis added*)

OA541 Bank25: "[A] manager (...) indicated it would be *safer* to maintain an undeclared account in Switzerland." (SOF25, 2015, *emphasis added*)

OA542 Bank28 "never had a strict policy against accepting clients with U.S. citizenship or U.S. tax liabilities… [Bank28] did not have significant *concerns regarding U.S. clients."* (SOF28, 2015, *emphasis added*)

OA543	Bank29: "[W]ith the knowledge that Swiss banking secrecy laws would prevent [Bank29] from disclosing [clients'] identities to the U.S. tax authorities." (SOF29, 2015)
OA544	Bank31 "recognized both risks and rewards of handling U.S. clients. (...) the Bank also recognized an opportunity to attract new U.S. clients because many Swiss banks declined to service U.S. persons from Ticino and the Bank perceived "a huge demand"." (SOF31, 2015)
OA545	Bank32's documents remained "*protected by Swiss banking secrecy laws.*" (SOF32, 2015, *emphasis added*)
OA546	Bank38: "asssessed *the risk (...) to fall under U.S. bank supervision as low* ." (SOF38, 2015, *emphasis added*)
OA547	Bank4X: "you had *the feeling you were not exposed*." (Int. 74, *emphasis added*)
OA548	Bank54 "enabled certain U.S. taxpayers to maintain undeclared accounts in Switzerland, where they could *shield their illegal activity from the United States through reliance on Swiss banking privacy and data-protection* laws ." (SOF54, 2015, *emphasis added*)
OA549	Bank54: "On at least five occasions, [Bank54] Switzerland relationship managers discussed Swiss bank secrecy laws with U.S. tax-payer clients, including in one instance how such laws *protected* client anonymity with respect to information about the U.S. taxpayer client's accounts at [Bank54] Switzerland." (SOF54, 2015, *emphasis added*)
OA550	Bank64: "The Bank was concerned about whether or not it might be *subject to sanctions by the U.S. government* . (...) one of the partners noted: *'The bank has no knowledge as to whether it is on the radar of the U.S. or not.* This cannot be excluded with 100% certainty, but since the bank never carried out any acquisitions on U.S. territory and never sought actively to acquire U.S. clients, *the risk can be considered to be minor.'*" (SOF64, 2015, *emphasis added*)
OA551	Bank66 "evaluated the *risk related to the exit of clients from UBS* and ultimately (...) decided to take only new U.S. clients that had a direct connection to Switzerland." (SOF66, 2015, *emphasis added*)
OA552	Bank6X: "That was not seen. People rather believed that I do everything only from Switzerland. I am *quasi safe* in the Swiss community." (Int. 85, *emphasis added*)
OA553	Bank72 "intended (...) *to ensure that the Bank 'is not audited by the American authorities .'"* (SOF72, 2015, *emphasis added*)
OA554	Bank75: "An internal [Bank75] memorandum stated: It should be recorded that *the business with U.S. clients does indeed pose a risk for our bank*. The measures taken, however, result in this *risk being reduced to a reasonable level*. It remains to be stated that the bank has already had banking ties with U.S. clients since at least 2007. The close and very good collaboration with all departments involved has, in my opinion, resulted in us being *able to control and monitor this risk*." (SOF75, 2015, *emphasis added*)
OA555	Bank78: "Why should we freely throw away a good business opportunity? (...) for 'security reasons' (...) general counsel then explained to CEO#2 that the proposal was based on the following considerations: 1) '[Bank78] *does not have any exposure in the U.S.A.* '" (SOF78, 2015, *emphasis added*)
OA556	Bank8X: "With the growing interdependence of diverging legal systems, we need to ensure that our business is always rooted *within one reliable legal system*. Only then, can we avoid incalculable risks." (Int. 16, *emphasis added*)
OA557	Swiss bankers' "understanding was consistent with the *long history of non-prosecution of Swiss banks and bankers* in the United States for assisting in tax evasion." (WegelinReply: 22, *emphasis added*)
OA558	"With the belief then that *secrecy was sacrosanct*" (...) "UBS and the bigger banks had people in other offices abroad [in the U.S.] promoting some of these schemes. Promoting the fact that you could park your money in Switzerland and that it *would be safe*. I think a lot of the institutions thought that they *were safe*. That *nothing could happen*." (Int. 10, *emphasis added*)
OA559	"So, during 20 years, no one really cared. Everyone [Swiss banks] knew that there was a rule somewhere but *no one really cared [about U.S. law]*." (Int. 11, *emphasis added*)
OA560	Bank8X "was thus convinced that (...) strictly speaking it was *not within the reach of US law enforcement*. With this *false sense of security*, Wegelin and several other local Swiss banks welcomed untaxed US clients expelled from Swiss banks such as UBS." (Troller et al., 2013, *emphasis added*)
OA561	"We have a relationship of trust between the citizen and the state. (...) *There only is a criminal prosecution when someone deliberately falsifies documents; then it's tax fraud*." (Interview; HandelsZeitung17/09/08, *emphasis added*)
OA562	For Swiss banks, "[b]ank secrecy laws and practices are *serving as a cloak* (...) for misconduct by banks colluding with clients to evade taxes, dodge creditors, and defy court orders." (DOJ, 2008: 8, *emphasis added*)
OA563	Excerpt from a letter sent by UBS senior executives to its U.S. clients in 2002: "[W]e should like to underscore that a Swiss bank which runs afoul of Swiss privacy laws will face sanctions by its Swiss regulator. ... [the] Swiss banking relationship is *as safe as ever* and that *the possibility of putting pressure on our U.S. units does not change anything*." (PSI, 2008: 87, *emphasis added*)
OA564	Swiss banks are "using our treaties *as a shield to* deny us tax information instead of using those treaties as a sword to expose tax cheats as was intended (...) providing *a safe haven* and promising *ironclad secrecy laws* for tax evaders." (PSI, 2009: 6, *emphasis added*)
OA565	Swiss banks used Swiss rules as "a sword - a means to encourage U.S. taxpayers to utilize their services in order to evade U.S. taxes - and a shield - by *taking advantage of the difficulty that bank secrecy poses to investigators* who would root out such tax evasion." (DOJ130225, 2013, *emphasis added*)
OA566	According to a Wegelin executive, "Wegelin *was not exposed to the risk of prosecution that UBS faced in the U.S.* (...) Wegelin was aware that this conduct was wrong. (...) However, Wegelin believed that, as a practical matter, it *would not be prosecuted in the United States for this conduct,* because it had no branches or offices in the United States and because of its understanding that it acted in *accordance with, and not in violation of, Swiss law*." (Wegelin's Indictment, JSR: 7-27; *emphasis added*)

OA567 “Swiss banks may have failed to understand that the United States’ long-standing policy of not *prosecuting wholly foreign banks for assisting in tax evasion* had changed [with the UBS case].” (WegelinReply: 23, *emphasis added*)

OA568 “[D]espite Wegelin’s awareness of the U.S. investigation of UBS, Wegelin approved the opening of these non-W-9 accounts based on its incorrect *belief that it would not be prosecuted in the United States.*” (WegelinReply: 21; *emphasis added*)

OA569 “From today’s perspective, this was based on a wrong assessment. (...) *We did not expect the U.S. to sue the bank in this situation. ”* (Interviewee; BaslerZeitung14/09/11; *emphasis added*)

OA570 “Wegelin’s explanation (...) that *a financial institution can hide behind its own law* as a defense to actively and knowingly assisting the citizen of another country in violating the law of their home country and evading the taxes of their home country.” (DOJ130225: 14*, emphasis added*)

OA571 The Swiss-focused banks believed “*they were not affected* and (...) some took over UBS employees and clients. (...) So, [they] thought (...) that they were not concerned. (...) Bank8X built after the UBS case... they built a business case, as Bank8X did, on attracting U.S. clients that were leaving UBS. (...) but this was pretty much the mentality there, '*We're Swiss. No one will touch us*.' (...) for Bank8X or for Bank8X or a local cantonal bank: We are in Switzerland: Only in Switzerland. You can come.” (Int. 5, *emphasis added*)

OA572 “Banks, according to my perceptions (...) have *long denied that there is any risk for them in this*. They still believed, in spring of 2010, that in principle it was not a problem for them, so they repressed it for a long time.” (Int. 8, *emphasis added*)

OA573 “I’m a [bank X] in canton [X]. And not using any means, no communication and so you really *focus on Swiss territory*. No one’s going to… -I *am below the radar screen- no one's going to notice me. (...) no one’s going to come in and knock on my door.* I can still keep on harboring U.S. clients.” (...) “[T]hey assessed that there was a zero risk with [BankX] because [BankX] was a purely domestic bank with purely domestic RMS, etc., and therefore the risk was zero. (...) They didn’t know how much risks they were taking. Otherwise, they would not have taken them at all.” (Int. 11, *emphasis added*)

OA574 “[T]ruly believed, as per our lawyers, that the secrecy model was *untouchable*.” (Int. 55, *emphasis added*)

OA575 “[T]hey just relied on the *protection of the Swiss state*.” (Int. 61, *emphasis added*)

OA576 “They’re so far away, *nothing much can happen to us*.” (Int. 71, *emphasis added*)

OA577 “And said, we are *not under US jurisdiction*. One was simply *not afraid of the Americans*. Which was stupid in retrospect. I have to admit that, of course.” (Int. 71, *emphasis added*)

OA578 “You always thought you were *safe here in Switzerland*. And on Swiss soil, Swiss law applies and the Americans *can’t do that*. They *can’t take action here either*.... If they want to do something, then they have to come via legal assistance or administrative assistance and then you can always look.” (Int. 78, *emphasis added*)

OA579 A *“purely Swiss"* approach protected our business from foreign interference. (Int. 15)

OA580 “As part of their sales pitch to U.S. taxpayer-clients who were fleeing UBS, at various times, BERLINKA, FREI, KELLER, and other Client Advisors told U.S. taxpayer-clients that their undeclared accounts at Swiss Bank A would not be disclosed to the United States authorities because (...) unlike UBS, it *did not have offices outside Switzerland,* thereby *making Swiss Bank A less vulnerable to United States law enforcement pressure.*” (Rakoff In DOJ, 20130225: 7, *emphasis added*)

EPISODE2 **UNPROSECUTED ORGANIZATIONS:**

LOCALS **PERCEPTION OF PROSECUTION THREAT**

Perceived threat of organizational illegality

OA581 “We do respect the past and how this business once worked, yet, recognize the change today and accept the new realities in the interest of our customer and in our own interest. (...) for that reason, we *feel obliged to commit to large changes* and to *correctly apply laws*. There is *no way past the legalization* of undeclared assets? No.” (Interviewee; HandelsZeitung13/10/10, *emphasis added*)

OA582 Bank1: Conduct such as “the opening of any new U.S. client account” or “travel to the United States” was *prohibited.”* (SOF1, 2015, *emphasis added*)

OA583 Bank2 “Beginning in May 2010, [Bank2] adopted measures to *avoid facilitating U.S. tax evasion*” Bank2 “obliged its staff to avoid a number of specific practices in light of these concerns. While the Bank did not restrict the services it would offer its clients, the Bank directed its employees to make clear to their clients that the Bank *would not support tax evasion*. (...) In 2011, the Bank, while disclaiming any obligation to ensure that their clients have fulfilled their tax obligations, directed its employees *not to take affirmative steps that would facilitate - or appear to facilitate - clients' breaches of their tax obligations or clients' deliberate attempts to conceal their assets*.” (SOF2, 2015, *emphasis added*)

OA584 Bank3 “published on its intranet a "Voluntary Disclosure Handbook" for various countries (including the United States), and a referral list of external service providers who were willing to advise clients concerning voluntary disclosure programs in these countries, including the IRS Offshore Voluntary Disclosure Program. (...) In October 2011, the Bank implemented a policy that stated that [Bank3] would *not provide any assistance to its clients in acts aimed at deceiving Swiss or foreign authorities, particularly tax authorities, by means of incomplete or otherwise misleading attestations*.” (SOF3, 2015, *emphasis added*)

OA585 Bank13’s “directive concerned aiding and abetting tax evasion and emphasized that *while the Bank was under no obligation to ensure that clients complied with their tax duties, neither could the Bank take any action to help clients hide money or evade their tax duties*. Among other things, the directive banned any transaction that could conceal the identity of a client including (...) transactions in which clients sought to use loopholes in international law.” (SOF13, 2015, *emphasis added*)

OA586 Bank14: “On July 1, 2011, [Bank14] issued a formal directive concerning tax matters. The directive stated that, *to prevent the aiding and abetting of tax evasion*, [Bank14] employees must not do anything that promotes or facilitates client breaches of their tax obligations or declarations or that conceals the existence of assets. For example, the directive prohibited employees from delivering or collecting cash at a client’s domicile or assisting in the concealment of a beneficial owner. But the directive stated that [Bank14] was not obligated to ensure that clients met their tax obligations and declarations.” (SOF14, 2015, *emphasis added*)

OA587 Bank19 “modified its policies and procedures to gradually *ensure that it no longer assisted undeclared U.S. taxpayers in evading U.S. income tax.”* (SOF19, 2015, *emphasis added*)

OA588 Bank21’s “management decided that the Bank should proactively monitor the tax-compliance of new clients. (...) It hired a Swiss law firm and a U.S. accounting firm to reach out to U.S. account holders to persuade them *to come into compliance with U.S. tax law*. (...) In 2013, as a further *tax-compliance measure,* the Bank’s management *prohibited* the moving of assets to offshore financial centers, the withdrawal of large cash amounts, the transfer of accounts held in the name of non-U.S. corporations, foundations, trusts, or other legal entities.” (SOF21, 2015, *emphasis added*)

OA589 Bank20: “Employees *were prohibited from* sending mailings to or sponsoring events in foreign countries, and client visits and recruitment abroad *were prohibited*. The policy also expressly stated that employees *could not consult with* clients to evade taxes, especially with respect to transferring money abroad.” (SOF20, 2015, *emphasis added*)

OA590 Bank26: “Under this strategy, the account opening process would cease *when doubts arose about a customer’s tax compliance*. Further, the strategy required that the bank close existing banking relationships within 12 months after *discovering that the account holder was not tax compliant.”* (SOF26, 2015, *emphasis added*)

OA591 Bank28 “modified its Anti-Money Laundering Circular to classify the opening of new client relationships with U.S. citizens and/or U.S. residents as *high risk* and subjected them to the approval of the Compliance Department and the Management.” (SOF28; 2015, *emphasis added*)

OA592 Bank31: “In April 2012, the private banking group implemented a procedure to freeze *U.S. Related Accounts deemed to be noncompliant.*” (SOF31, 2015, *emphasis added*)

OA593 Bank32: “In or about late 2011, [Bank32] created a cross-border team based in its (...) headquarters to help bank personnel *properly service* account holders residing outside of Switzerland.” (SOF32, 2015, *emphasis added*)

OA594 Bank38: “In November 2011, the executive board concluded that the Bank's policy regarding new U.S. customers *needed to be "rethought."*” (SOF38, 2015, *emphasis added*)

OA595 Bank37 sought “to *bring the accounts into compliance.”* (SOF37, 2015, *emphasis added*)

OA596 Bank41 “adopted *additional precautions regarding the tax status of the* Bank's foreign account holders. This policy set forth an *internal compliance regime*.” (SOF41, 2015, *emphasis added*)

OA597 Bank59 started “dealing with openly declared black money and domiciled companies.” (SOF59, 2015)

OA598 Bank61 implemented “series of measures and reforms specifically intended to *ensure that its clients complied*.” (SOF61*, emphasis added*)

OA599 Bank64: “In a memorandum dated August 15, 2012 discussing an internal audit, one of the Bank’s partners wrote: (...) *accusations and procedures regarding the banks having assisted U.S-domiciled persons to commit tax evasion.*” (SOF64, 2015, *emphasis added*)

OA600 Bank66 “In December 2010, [Bank66] decided to *prohibit* new relationships with *U.S.-resident clients* and to scale back on existing relationships with such persons.” (SOF66, 2015, *emphasis added*)

OA601 Bank68: "Beginning in mid to late 2008, in the wake of the UBS investigation and deferred prosecution agreement, [Bank68] "instituted policies that were intended to *limit its potential criminal and civil tax liability by ensuring compliance with U.S. laws*." (SOF68, 2015, *emphasis added*)

OA602 Bank70: "...establishing a *U.S. Competence Center to ensure that policies and procedures adequately addressed regulatory requirements*." (SOF70, 2015, *emphasis added*)

OA603 Bank75 “Through its managers, employees and/or others, [Bank75] knew or had reason to know that *some U.S. taxpayers who had opened and maintained accounts at the Bank were not complying with their U.S. income tax and reporting obligations*.” (SOF75, 2015, *emphasis added*)

OA604 Bank77: “In June 2013, the Bank implemented *more restrictive and effective measures concerning its U.S. cross-border business*. In its account opening process adopted that month, a new client must confirm tax compliance with respect to its assets and income and affirm that it will continue to remain tax compliant. [Bank77’s] private banker or relationship manager then verifies the client’s declaration and confirms this assessment in writing.” (SOF77, 2015, *emphasis added*)

OA605 Bank77 “only wanted to have *tax compliant clients*.” (SOF77, 2015, *emphasis added*)

OA606 Bank79 “knew in August 2010 that *some of its existing U.S. customers were not tax-compliant*” (...) “During the calendar year 2010, board of directors meeting minutes and management board meeting minutes discussed *the U.S. customers as a risk*. The management board meeting minutes dated June 7, 2011 state that measures needed to be taken regarding *U.S. customers whose accounts could not be regarded as "tax compliant beyond a doubt"* (translated from German). On or about June 30, 2011, Executive #1 drafted a document entitled *"Risk Assessment [Bank79] Cross-Border Transactions with U.S. Clients"* (translated from German). This document was *the first written risk assessment of the U.S. cross-border business that was completed by [Bank79] and was provided to the Bank's board of directors*. (...) Executive #1 included statistics as part of this risk assessment that made clear that *many U.S. accounts without signed Forms W-9 also remained at the Bank*. (...) On June 25, 2012, the minutes of the meeting of the management board state that all U.S. client accounts with “unclear tax status still existed on June 25, 2012.”” (SOF79 ; 2015, *emphasis added*)

OA607 Bank9X: “I don’t want to feel like that. I thought I’m a banker. I worked for a reputable institution… I mean, a lot of employees in the industry suddenly felt that they were looked at as *criminals* as part of a *criminal organization*.” (Int. 87, *emphasis added*)

Perceived threat of prosecution by the foreign regulator

OA608 Bank14’s “annual fee revenue generated by the External Asset Manager for the Bank cannot be classified as insignificant' and must be weighed against '*the risk situation of the bank*.'” (SOF14, 2015, *emphasis added*)

OA609 Bank14: “According to the meeting minutes, a member of the Board ("Board Member #I") began the discussion by stating: ‘Although in the past, these transactions generated a nice amount of income, the Bank's security is the top priority.’ (...) There is *the latent risk that previous revenues from this U.S. strategy of [Bank14] are seized or that corresponding fines are imposed on the bank*. Based on the current developments in the industry, *the probability of occurrence has increased*.” (SOF14 , 2015, *emphasis added*)

OA610 Bank19 “began providing each new U.S. client with a disclaimer.” (SOF19, 2015)

OA611 Bank1X: “…you really didn't feel like you were guilty of anything, but probably the smartest thing to do given the *potential risk* of not even being convicted in the U.S., but *being hauled into an American court*, having to defend yourself there, and imagining the consequences that that could have.” (Int. 91, *emphasis added*)

OA612 Bank1X: “Apart from that, you would have had to travel there, defend yourself and pay the lawyers to do it. It would have taken months. It would have been very complicated. It was a *risk* we didn't want to take.” (Int. 91)

OA613 Bank20: “In August 2013, the Bank revised its Cross-Border Policy to expressly state that the Bank would not open new accounts from persons in '*high risk countries,' including the United States.*” (SOF20, 2015, *emphasis added*)

OA614 Bank29’s “objective was *to avoid any risk related to the violation of norms.*” (SOF29, 2015, *emphasis added*)

OA615 Bank34: “[T]he relationship manager identified several withdrawals from [Bank34] as "*high-risk*" because they totaled at least $250,000 and the primary owner was a U.S. beneficial owner.” (SOF34, 2015)

OA616 Bank38: “At a meeting of the [Bank38] Bank executive board on February 8, 2012, (...) another stated: ‘[T]*the Department of Justice could also file a suit against us.*’” (SOF38, 2015, *emphasis added*)

OA617 Bank64’s “*major risks lie in the field of business relationships with account holders and/or beneficial owners domiciled in the USA.*” (SOF64, 2015, *emphasis added*)

OA618 Bank75: A manager stated “it was *'really dangerous*,' tantamount to 'giv[ing] [the customer] the rope (to hang themselves).'” (SOF75, 2015, *emphasis added*)

OA619 Bank78: “[T]he head of private banking for EAM #1 informed Private Banker #2 that EAM #1 had decided to separate from its asset management business with U.S. clients due to *risk* considerations." (SOF78, 2015, *emphasis added*)

OA620 “[The Wegelin case] has led to an *intensification of risk* and compliance thinking.” (Int. 65, *emphasis added*)

OA621 “If you are honest, by now, no bank with a former U.S. offshore business can claim that it cannot get into *the U.S.'s sight.”* (Interviewee, HandelsZeitung10/03/10, *emphasis added*)

OA622 “There is a latent risk - and *by now the Americans are also targeting [other banks].*" (Interviewee; Bank3X; HandelsZeitung17/01/13, *emphasis added*)

OA623 The Wegelin case “has ultimately shown that *the risk assessment - that I simply limit my business to Switzerland only, and that in practice I am safe then. This assessment is wrong.*” (Int. 8, *emphasis added*)

UNPROSECUTED ORGANIZATIONS:

COMPLIANCE WITH FOREIGN RULES

BEFORE EPISODE 1: BOTH GROUPS

UNPROSECUTED ORGANIZATIONS:

COMPLIANCE WITH FOREIGN RULES

Non-compliance before Episode 1

OA624 Bank1X: “We never had a business model that focused on funds that were not tax compliant. But we always started from the idea that the customer is responsible for these obligations. We had not dealt with this issue before. We prepared our tax statements once a year *without necessarily paying attention to whether or not they were filed with the IRS*.” (Int. 91, *emphasis added*)

OA625 Bank36: “Prior to August 1, 2008 (...) U.S. taxpayers were able to continue depositing funds into accounts at [Bank36] (...) [Bank36] was aware that some of its U.S. clients wanted to conceal their accounts from U.S. authorities.” (SOF36; 2015)

OA626 Bank5X: “They didn’t really care about that. They said that it was *up to the customer* what he wanted to do.” (Int. 78, *emphasis added*)

OA627 Bank8X: “This very idea: ‘*Taxes* from the customer *are not our job*. After all, we are a bank and we do banking services.’” (Int. 79, *emphasis added*)

OA628 Bank9X: “The general attitude of Swiss banks was, *it’s not our issue*. You’re responsible for your taxes. I'm not doing your taxes. I don't ask about taxes, because it's not my issue.” (Int. 87, *emphasis added*)

OA629 Bank9X: “Until then, it was quite clear that *whether they were taxed or not* was the customer’s problem, primarily. So, there is the US American saying or English saying ‘don’t make your clients problems your own.’” (Int. 73, *emphasis added*)

OA630 Bank9X: “I would say that practically nobody in the whole financial center was aware *that this interpretation that taxes are a customer issue, that that’s wrong*.” (Int. 73, *emphasis added*)

OA631 “It was not specific to the US, it was a general state of mind of Swiss private banks until the end of the 2000.” (Int. 70)

OA632 “The tax risk was not identified as such, because in general, Swiss banks were pretty *color-blind, as far as tax compliance was concerned*.” (Int. 70, *emphasis added*)

OA633 “I’ve worked now more than 35 years for Swiss banks, and for the first 15 years all the questions having to do with US, *they were never tax questions*.” (Int. 70, *emphasis added*)

OA634 “In the old world, and I cannot emphasize this enough, the banks were completely *insensitive to the tax issue*. They really ignored it. As a rule, they didn’t really know where the customer was liable to pay taxes. They didn’t ask whether he was an American or a German or both. So, one was very, very insensitive.” (Int. 71, *emphasis added*)

OA635 “All Swiss banks did that. We just did business the way we did with German clients, or with French clients. They didn’t ask. They didn't want to know. And that was the end of the matter. *Tax evasion was simply the customers’ problem*.” (Int. 86, *emphasis added*)

EPISODE1

COSMO-POLITANS

UNPROSECUTED ORGANIZATIONS:

COMPLIANCE WITH FOREIGN RULES

Compliance

<u>*Compliance with US law*</u>

OA636 Bank8: “Beginning in 2009, [Bank8] undertook a thorough review of its policies and procedures in an effort to improve and enhance its controls over accounts, account documentation, and the *tax compliance of its account holders*.” (SOF8, *emphasis added*)

OA637 Bank9: “The tax compliance status of the first 11 accounts was proven (...) in order to *comply with [U.S. law].”* (SOF9, 2015, *emphasis added*)

OA638 Bank10: “all services be rendered *in compliance with local laws and regulations.*” (SOF10, 2015, *emphasis added*)

OA639 Bank24: “...ensuring the Bank’s compliance with *all applicable U.S. laws.”* (SOF24, 2015, *emphasis added)*

OA640 Bank33 “has conducted an extensive program to ensure its *ongoing compliance* with [U.S. law].” (SOF33, 2015, *emphasis added*)

OA641 In 2010, [Bank40’s] “Management Board reinforced its policy of requiring account holders and beneficial owners to completely fill out the Declaration of *Non-U.S. Person Status.*” (SOF40, 2015, *emphasis added*)

OA642 Bank55: “Following the U.S. government’s 2008 investigation and 2009 deferred prosecution agreement with UBS, [Bank55] *modified its practices and procedures* for U.S. taxpayer clients.” (SOF55, *emphasis added*)

OA643 Bank56: “In 2009, approximately concurrent with the announcement of the UBS Deferred Prosecution Agreement, the Bank began to undertake efforts to induce U.S. clients to *become compliant*.” (SOF56, *emphasis added*)

OA644 Bank76: “Beginning in mid to late 2008, in the wake of the UBS investigation and prosecution, [Bank76] instituted *policies that were intended to limit its potential criminal and civil tax liability*.” (SOF76, *emphasis added*)

OA645 Bank8X: “We’ve said if we have problems, we’ll go over there and take the Bull at the Horn. We’ll go over to the USA.... I haven’t heard of anyone else proactively going over there. And, of course, that was a courageous decision, I have to say, there were also certain risks, of course. If it is then actually only UBS, and then stupid as we are still, we deliver ourselves on the gold tablet… we went there, said, look, we have the same problem, we want to go clarify this now.” (Int. 56)

OA646 After the UBS case, “the big banks said, 'Everything, everything, *everything must be done*. [comply with foreign laws and regulation] And the others said the opposite.” (Int. 2, *emphasis added*)

OA647 “[I]t still took some time before things changed, and although some banks started looking at tax compliance, I would say *only a minority* (...) What they did, is they started trying to clear their books of their non-compliant clients or to deal with that in a more prudent manner. (...) So, the banks *who started dealing with this, I would say it's one category of banks* (...) the banks here which, back in 2008, started *cleaning their* books, *cleaning their act in terms of soliciting clients in the U.S. by stopping it or registering an entity that is an investment advisor that is allowed to do this*. This is what [Bank8X], for instance, did. [Bank8X] also did that and others followed afterwards.” (Int. 5, *emphasis added*)

OA648 “The sights are no longer on illegal, but on *legal funds*.” (Int.; Bilanz07/03/08, *emphasis added*)

OA649 “I know of many banks that *strongly reworked their business policy in the aftermath of UBS*. According to my state of knowledge, only few institutions systematically accepted UBS clients.” (Int.; HandelsZeitung17/01/13, *emphasis added*)

<u>*Exiting of all U.S. clients*</u>

OA650 In 2009, “we actually did what you would have expected from a Swiss bank. Namely, we took the UBS case seriously enough to really *do without that form of business*.” (Int. 68, *emphasis added*)

OA651 In 2009, [Bank5’s] “Management Board, with participation of the then-Chairman of the Board, decided to *terminate all U.S. account relationships* in response to the issues at UBS.” (SOF5, 2015, *emphasis added*)

OA652 Prior to June 2010, [Bank14] “did not have any U.S.-specific business policies. Thereafter, the Bank decided *not to do further business with any U.S. clients*.” (SOF14, 2015, *emphasis added*).

OA653 [Bank18] “later determined that it did not want the administrative burden of maintaining U.S. related accounts. Accordingly, in 2009, [Bank18] began implementing a policy requiring that *private banking accounts of individual U.S. taxpayers be closed*.” (SOF18, 2015, *emphasis added*)

OA654 Bank24: “In early 2009, the Bank decided to *discontinue business relations* with U.S. residents and U.S. citizens as account holders or beneficial owners.” (SOF24, *emphasis added*)

OA655 From July 2008, [Bank30’s] “formal policy has been to reject all clients who qualified as taxable under U.S. law. When the Bank later discovered clients, who were in fact subject to U.S. taxation, the Bank sought to *terminate the relationships* with those clients.” (SOF30, 2015, *emphasis added*)

OA656 In October 2009, the [Bank35] “issued a U.S. client directive ("the 2009 Policy"), which formalized the July 2008 decision, and started to *exit U.S. clients* on a case-by-case basis. During 2009 and 2010, the Bank decided on a general exit of U.S. clients. As part of this strategy, the Bank closed 24 accounts for which a U.S. taxpayer was the account holder or beneficial owner.” (SOF35, 2015, *emphasis added*)

OA657 Bank51: “[W]hen the Bank became aware that a client was *a citizen or resident of the U.S. (a "U.S. person"*), it took steps to close the account (or block it pending clarification of the client's U.S. status).” (SOF51, 2015, *emphasis added*)

OA658 Bank5X: “We concluded that we would *exit the whole U.S. book*. At that time, that was about just under 800 million under management, that was a lot for us at that time, 800 million. We had about ... six billion under management at that time. So, it was more than ten percent. That’s a momentous decision.” (Int. 68, *emphasis added*)

OA659 Bank8X “and other banks have shown no interest in taking over [undeclared] clients from UBS. (...) The *business relationships are terminated only with those customers who do not want to cooperate with the supervisory and tax authorities*. There are no problems with registered American customers.” (Int.; Bank8X; Handelszeitung17/09/08, *emphasis added*)

<u>*Requiring W9 form and banking secrecy waiver ("Regularize or leave")*</u>

A1) Cantonal banks

OA660 Bank36: “Beginning in August 2008, [Bank36] adopted measures to avoid facilitating U.S. tax evasion. In August 2008, Bank36 mandated that no new funds would be accepted from U.S. residents without a signed IRS Form W-9. In March 2010, the Bank decided for all existing U.S. clients to *either provide an IRS Form W-9* or withdraw their funds from the bank.” (SOF36, *emphasis added*)

OA661 Bank36: “In March 2010, the bank decided to ask all existing U.S. clients to *either provide an IRS Form W-9 or withdraw their funds from the bank*. (...) In spring of 2011, [Bank36] started to send letters to account holders with an identified U.S. nexus and request that they provide a signed IRS Form W-9.” (SOF36, 2015, *emphasis added*)

OA662 In December of 2008, Bank39 ensured clients’ “compliance with their *U.S. tax obligations,* (ii) *waive Swiss banking secrecy, and (iii) provide a Form W9*.” (SOF39, 2015, *emphasis added*)

OA663 Bank39, in December 2008: “New U.S. clients who failed to submit the requested documents were not supposed to be accepted (...) without *securing a Form W9*. Existing clients who failed to do so were to be exited by June 2010. In July of 2009, the Bank stopped accepting any new U.S. clients.” (SOF39, 2015, *emphasis added*)

OA664 Bank39: “In December 2008, the Bank required that all of the Bank’s new and existing U.S. clients, irrespective of domicile; (i) submit a handwritten declaration of compliance with their U.S. tax obligations; (ii) waive Swiss banking secrecy; and (iii) *provide an IRS Form W9*. In July of 2009, the Bank stopped accepting any new U.S. clients, with the exception of U.S. nationals residing in Switzerland or Swiss nationals temporarily residing in the United States.” (SOF39, *emphasis added*)

OA665 Bank50: “On November 20, 2008, Bank50 decided not to conduct business with U.S. clients unless these persons were willing to disclose their assets deposited with the [Bank50] to U.S. tax authorities. Later in 2009, Bank50 also started to require U.S. clients who did not hold U.S. securities in their accounts to *provide W-9 forms* and other documentation to curb the violations of U.S. tax laws.” (SOF50, *emphasis added*)

OA666 Bank74 sought “to ensure that new U.S. clients were *in compliance with their U.S. tax obligations,* in 2009.” (SOF74, 2015, *emphasis added)*

OA667 "[Bank8X] and [Bank8X] sent such letters to customers abroad as early as in 2009. [Bank88] told them that it considers itself "properly authorized" to "disclose the identity of the customer" to a dozen countries, including the U.S. (...). Whoever does not accept it, has to withdraw their money.” (Int.; Bank8X; DerBund28/04/12)

OA668 Bank8X: “we decided that we would no longer accept customers domiciled in the U.S.” (Int.; Bank8X; BaslerZeitung14/09/11)

OA669 Bank82: “As of June 2008, Bank82's Private Banking Unit began to require that new clients who were U.S. nationals or U.S.-domiciled and who were transferring securities accounts to Bank82 from UBS *submit a Form W-9*... In June 2009, Bank82 decided to close its business with all U.S.-domiciled clients.... For U.S. clients domiciled outside of the U.S., Bank82 sought a Form W-9 so that it could determine that the U.S. clients were compliant with their U.S. tax obligations.” (SOF82, *emphasis added*)

A2) Banks with foreign owners

OA670 Bank4: “In 2009, the Bank initiated a process to contact U.S. taxpayers to encourage them to regularize or close their accounts. In the spring of 2009, the Bank initiated a process to ascertain whether U.S. clients holding accounts with the Bank *had signed an IRS Form*

W-9. When U.S. clients held accounts without an IRS Form W-9 on file, the Bank either sought to obtain confirmation that the client had regularized the account or requested the closure of the account." (SOF4, 2015, *emphasis added*)

OA671 Bank4: "In the spring of 2009, the Bank initiated a process to ascertain whether U.S. clients holding accounts with the Bank had signed *an IRS Form W-9*. When U.S. clients held accounts without an IRS Form W-9 on file, the Bank either sought to obtain confirmation that the client had regularized the account or requested the closure of the account." (SOF4, *emphasis added*)

OA672 Bank6: "Starting in 2010, the Bank implemented a policy *requiring all U.S. clients to provide evidence that their accounts were disclosed to the Internal Revenue Service (i.e., Forms W-9) or, if not, the Bank was to terminate their relationship with the U.S. account holder."* (SOF6, 2015, *emphasis added*)

OA673 Bank6: "Starting in 2010, the Bank implemented a policy requiring all U.S. clients to provide evidence that their accounts were disclosed to the Internal Revenue Service (i.e., *Forms W-9*) or, if not, the Bank was to terminate their relationship with the U.S. account holder." (SOF6, *emphasis added*)

OA674 Bank8 "…instituted *a legacy account remediation project beginning in early 2010*. Pursuant to that project, each account held directly or indirectly by a U.S. taxpayer was required to produce evidence of U.S. tax compliance; if such accounts did not produce the required documentation, the account was terminated." (SOF8, 2015, *emphasis added*)

OA675 Bank8: "From April 2010 forward, Bank8 accepted an account for an individual U.S. taxpayer only if he or she was resident outside the U.S. and only if the prospective account holder executed an *IRS Form W-9*. Bank8 instituted a legacy account remediation project beginning in early 2010. Pursuant to that project, each account held directly or indirectly by a U.S. taxpayer was required to produce evidence of U.S. tax compliance." (SOF8, *emphasis added*)

OA676 Bank10 engaged in a "process to contact U.S. taxpayers to encourage them *to regularize or close their accounts*." (SOF10, 2015, *emphasis added*)

OA677 Bank12: "On March 26, 2009, Bank12 issued a directive to its employees, requiring that any U.S. individuals seeking to open an account comply with the following requirements: a. U.S. individuals were required to provide a signed IRS *Form W-9*." (SOF12, *emphasis added*)

OA678 Bank24 "determined that all individual U.S. clients and offshore structures should be transferred to another bank by August 31, 2009, and further decided to terminate such relationships if not transferred by September 15, 2009. As of June 2009, the Bank had identified 28 such accounts." (SOF24, 2015)

OA679 Bank30: Since "July of 2008, its formal policy has been to reject all clients who qualified as taxable under U.S. law." (...) It became "an explicit element of [Bank30's] business model not to accept or service any U.S. clients." (SOF30, 2015)

OA680 Bank33: "In June 2009, *the Bank decided to terminate all relationships with U.S. Related Account Holders who would not provide a Form W-9*. In July 2009, the Bank implemented this decision by sending a letter to (i) all clients resident in the U.S., with certain limited exceptions (e.g., if the account was dormant), and (ii) all other clients having a U.S. connection who did not have a Form W-9 on file. U.S. Related Account Holders were asked to submit a Form W-9 within 60 days; the Bank terminated the accounts of those account holders who refused to do so." (SOF33, 2015, *emphasis added*)

OA681 Bank33: "management [reviewed] the Bank's policy concerning U.S.-related accounts in 2008. In June 2009. the Bank decided to terminate all relationships with U.S. Related Account Holders who would not provide a Form W-9." (SOF33, 2015)

OA682 Bank40 in 2010: "Despite declining assets under management, the Bank maintained its increasingly restrictive policies and its *efforts to increase transparency, including eliminating U.S. clients."* (SOF40, 2015, *emphasis added*)

OA683 Bank40: "In early 2009, Bank40 made various policy changes designed to increase transparency and *eliminate non-tax compliant accounts*, including accounts held by U.S. clients." (SOF40, *emphasis added*)

OA684 Bank42: "In April 2010, [Bank42] adopted a *"W-9 or Exit" policy* that required all U.S. clients to provide evidence that their accounts were disclosed to the IRS (which in the Bank's view included IRS Forms W-9). U.S. clients that did not provide the required evidence would have their account relationships terminated by the Bank." (SOF42, 2015, *emphasis added*)

OA685 Bank42: "In response to the UBS deferred prosecution agreement, Bank42 implemented its *W-9 or Exit Policy* in early 2010." (SOF42, *emphasis added*)

OA686 Bank43: "U.S. citizen clients had to *provide a Form W-9* and *execute a banking secrecy* waiver"... "In 2009, [Bank43] implemented *a "Regularize or Leave" action plan* that required all U.S. taxpayer-clients holding, directly or indirectly, accounts at the Bank to (I) establish their U.S. tax compliance; (ii) participate in a voluntary disclosure program; or (iii) close their accounts. As a result of the "Regularize or Leave" action plan, 42% of the U.S. taxpayer accounts known in August 2008 to lack positive evidence of U.S. tax compliance were closed or remediated by the end of 2010. (...) [Bank43] closed approximately 238 U.S. Related Accounts between August 1, 2008 and December 31, 2013, totaling approximately $228 million in assets under management." (SOF43, 2015, *emphasis added*)

OA687 Bank43: "In 2009, Bank43 implemented a "Regularize or Leave" action plan that required all U.S. taxpayer-clients holding, directly or indirectly, accounts at the Bank to (I) establish their U.S. tax compliance; (ii) participate in a voluntary disclosure program; or (iii) close their accounts...." (SOF43)

OA688 Bank51: "the Bank generally required all new accountholders to provide *IRS Forms W-8 and W-9* or their equivalent, regardless of whether those account holders were going to invest in U.S. securities. In March 2009, this practice was formalized into Bank policy." (SOF51, *emphasis added*)

OA689 Bank53: "In August and September of 2009, a Bank employee informed an account holder that the Bank had adopted a new U.S. Related Account policy (...) the Bank's policy that *non-compliant U.S. accounts be closed*." (SOF53, 2015, *emphasis added*)

OA690 Bank53: "The Bank decided to close the accounts if they did not have the *requested documentation* (i.e., Form W-9 and Certification-Waiver)...The Bank also instructed its private bankers in writing to invite their U.S. clients (even those who were asked to close their

accounts) to consult their U.S. tax experts and, if necessary, regularize their tax status with U.S. authorities." (SOF53, *emphasis added*)

OA691 Bank55: "In December 2010, the Bank's Board of Directors *determined that any account owned by a U.S. person without the required (...) documentation, such as Form W-9 and bank secrecy waiver, would be closed.*" (SOF55, 2015, *emphasis added*)

OA692 Bank55: "In June 2009, the Bank's Board of Directors further updated the policy for U.S. persons. Specifically, this new policy required that all U.S. person-clients must provide a signed Form W-9 and waiver of bank secrecy." (SOF55)

OA693 Bank57: "Following local implementation of the Global U.S. Person Policy, the Bank, including specifically assigned relationship managers, sought a new Form W-9 from all clients who previously had declared themselves as U.S. persons on the Declaration U.S. Person/NonU.S. Person form, and, where clients refused to provide a Form W-9, the Bank informed clients that their accounts must be closed. The Bank referred to this as the "*regularize or leave" efforts*. (...) Pursuant to the Bank's "regularize or leave" and other efforts related to accounts held by U.S. persons, between August 1, 2008, and December 31, 2012, the Bank closed 381 accounts held or beneficially owned by U.S. persons with total assets under management of $523.2 million as of each account's penalty date." (SOF57, 2015, *emphasis added*))

OA694 Bank57: "In August 2008, as part of a broader effort to enact policy initiatives that would apply globally to all Bank57 businesses engaged in cross-border private wealth management business, Bank57 enacted a Global Private Wealth Management - U.S. Person Policy (-'Global U.S. Person Policy"). Published formally in October 2008, ... it required all U.S. persons to submit an *IRS Form W-9* in order to do business with Bank57 entities outside the United States. Following the enactment of the Global U.S. Person Policy, Bank57 Suisse management hosted town hall meetings and issued guidance to introduce the policy and other cross-border compliance efforts. Where clients refused to provide a Form W-9, the Bank informed clients that their accounts must be closed. The Bank referred to this as "regularize or leave" efforts." (SOF57, *emphasis added*)

OA695 Bank62: "Almost immediately, [Bank62] also adopted a "Regularize or Leave" policy requiring closure of existing accounts of U.S. taxpayers who failed to provide the Bank with evidence of U.S. tax compliance. [Bank62] supplemented these policies with additional details and procedures in September and October 2009, and further elaborated upon them through a cross-border business directive issued July 2010." (SOF62, 2015)

OA696 Bank62's "business relationships only with non-U.S. resident clients who could provide documentation of their compliance with *U.S. tax law* (...) accounts for non-U.S. entities that had beneficial owners who were residents or citizens of the United States, if the entity was treated as a corporation for *purposes of U.S. tax law* (...) unless (1) *U.S. tax law* treated the entity as a non-corporate "flow-through" entity." (SOF62, 2015, *emphasis added*)

OA697 Bank62: "On February 27, 2009, [the bank] adopted new policies restricting the opening of new accounts involving U.S. taxpayers. Almost immediately, [the bank] also adopted a "Regularize or Leave" policy requiring closure of existing accounts of U.S. taxpayers who failed to provide the Bank with evidence of U.S. tax compliance." (SOF62)

OA698 Bank65: "in the last quarter of 2008 [Bank65] implemented an "Exit Program" for U.S. taxpayer-clients who did not provide or did not have on file a properly signed Form W-9, which the Bank viewed as the necessary U.S. tax compliance information (...). [Bank65] implemented this program by *either obtaining a properly signed Form W-9 from each U.S. taxpayer-client or closing the account*." (SOF65, 2015, *emphasis added*))

OA699 Bank68: "Recognizing that certain accounts had been opened under prior policies without a Form W-9, [Bank68] instituted *a legacy account remediation project* beginning in October 2008. Thereafter, [Bank68] affirmatively required every existing U.S. client account to provide a signed, valid Form W-9 (...). If the existing U.S. client did not provide a Form W-9, [Bank68] eventually terminated the account relationship." (SOF68, 2015, *emphasis added*)

OA700 Bank69: "On July 31, 2008, [Bank69] decided, in light of the UBS investigation by U.S. authorities, to take immediate measures to strengthen its compliance framework for U.S. clients. On that day, it communicated to all employees that [Bank69] among other things, (i) would not open any new accounts for U.S. clients unless they submitted an *IRS Form W-9;* and that it (ii) would not open new accounts for structures with a U.S. beneficial owner. In November 2008, [Bank69] communicated to its employees new general principles applicable to U.S. clients, which replaced the measures taken in July 2008. The principles stated that accounts of U.S. clients who did not disclose their assets to the IRS would be exited. In addition, with respect to U.S. domiciled clients, [Bank69] would only serve as a custodian bank (...) Short deadlines were set to relationship managers to bring the existing U.S. client population in line with these principles." (SOF69, 2015, *emphasis added*)

OA701 Bank69: "*insufficient effort was made to determine whether such an entity was valid for U.S. tax purposes*. (...) undertook measures and reforms specifically intended to ensure that its clients complied with their *applicable U.S. tax and reporting obligations*". (SOF69, 2015, *emphasis added*)

OA702 Bank69_1: "In November 2008, Bank69_1 communicated to its employees new general principles applicable to U.S. clients, which replaced the measures taken in July 2008. The principles stated that accounts of U.S. clients who did not disclose their assets to the IRS would be exited.... In the second half of 2008, following the events at UBS, Bank 69_1began to adopt more restrictive business policies with regard to new accounts for U.S. clients, accepting new U.S. clients only if they had been declared to the IRS. In the first half of 2009, Bank 69_1decided to close its existing U.S. accounts. (SOF69)

OA703 Bank70: "In June 2009, the Bank launched a tax compliance review of all accounts with a broadly defined U.S. nexus. External counsel determined that a Form W-9 was required for individual account holders." (SOF70).

OA704 Bank70: "In March 2009, (...) also informed that the Bank was required to obtain a Form W-9 from U.S. taxpayers and to report these U.S. taxpayers according to [U.S.] rules. Also in December 2009, as an additional control measure, the Bank started to centralize the servicing of all U.S. clients (except for "banking only" clients) at the U.S. Competence Center." (SOF70, 2015)

OA705 Bank88: "UBS 2008: After learning this information, Bank88 Switzerland ... began a series of policy changes... In February 2009, Bank88 Switzerland decided to close accounts for U.S. persons who had $1 million or less in AUM. Bank88 Switzerland permitted those U.S. clients holding more that $1 million to keep their accounts only if they signed a Form W-9, a waiver of Swiss bank secrecy, and a declaration of tax compliance. (SOF95).

OA706 Bank94: "In December 2008, the Board of Management decided to require all U.S. taxpayers holding accounts at Bank94, including accounts held through structures, to provide IRS Forms W-9 to Bank94 and thereby permit Bank94 to report their accounts, and the income earned in the accounts, to the IRS." (SOF94)

A3) Internationally oriented CH banks

OA707 Bank46: "On May I, 2009, Bank46 announced a policy and strategy going forward with respect to U.S. clients, which stated or reiterated, as applicable, among other things, that: ... new individual clients that were U.S. persons would be accepted only if such persons signed an IRS Form W-9 as part of this effort, relationship managers were asked to contact U.S. clients who had not provided an IRS Form W-9 to either *obtain an IRS Form W-9* from the U.S. client; or *terminate the U.S. client's business relationship* with Bank46." (SOF46, *emphasis added*)

OA708 Bank65: "In the last quarter of 2008 [Bank65] implemented an "Exit Program" for U.S. taxpayer-clients who did not provide or did not have on file a properly signed Form W-9, which the Bank viewed as the necessary U.S. tax compliance information (...). [Bank65] implemented this program by *either obtaining a properly signed Form W-9 from each U.S. taxpayer-client or closing the account*." (SOF65, 2015, *emphasis added*)

OA709 Bank65: "In the last quarter of 2008 Bank65 implemented an "Exit Program" for U.S. taxpayer-clients who did not provide or did not have on file *a properly signed Form W-9*, which the Bank viewed as the necessary U.S. tax compliance information." (SOF65, *emphasis added*).

OA710 Bank68: "Beginning in approximately September 2008, [the bank] instituted a formal policy prohibiting relationship managers from opening new accounts for U.S. clients unless the U.S. client first provided a Form W-9. Recognizing that certain accounts had been opened under prior policies without a Form W-9, [the bank] instituted a legacy account remediation project beginning in October 2008. Thereafter, [the bank] affirmatively required every existing U.S. client account to provide a *signed, valid Form W-9*." (SOF68, *emphasis added*)

OA711 Bank71: "According to the Bank's March 4, 2010 policy... the United States person authorized Bank71 to disclose any and all information regarding his or her relationship with Bank71 to administrative, tax, or judicial authorities in Switzerland or the United States, as required by applicable law or regulations." (SOF71)

OA712 Bank74 "implemented a policy to prohibit the opening of new U.S. taxpayer accounts unless the account holders provided Forms W-9 and *bank secrecy waivers authorizing the Bank to disclose the account holders' identification information*. (...) Entities were also required to certify that their U.S. beneficial owners were *compliant with U.S. tax reporting obligations*." (SOF74, 2015, *emphasis added*)

OA713 Bank74: "Shortly thereafter, [Bank74] implemented a Regularize or Leave Action Plan (the "RLAP"), the tenets of which were described in a written policy, dated October 8, 2008 (the "RLAP Policy Document"), to be communicated verbally to the group heads of [Bank74's] private banking business unit. Pursuant to the RLAP, the Bank's management required that relationship managers instruct *each of their U.S. clients to sign a Form W-9*, voluntarily disclose their accounts to the IRS, or close their accounts." (SOF74, 2015, *emphasis added*)

OA714 Bank74: "In response to the April 2008 news reports on the IRS investigation of UBS, Bank74's senior management decided, in June 2008, to prohibit new U.S. taxpayer clients coming from UBS and to refrain from hiring UBS relationship managers with U.S. taxpayer clients. Shortly thereafter, Bank74 implemented a *Regularize or Leave Action Plan* (the "RLAP")." (SOF74, *emphasis added*).

OA715 Bank8X: "[We] have reduced the number of U.S. customers still remaining with us. We recommend the rest of the customers to *legalize and repatriate their money*." (Interviewee; Bank8X; TagesAnzeiger28/07/09, *emphasis added*)

OA716 Bank8X: "*We have no U.S. residents who do not declare their funds*. We serve American customers only through our Geneva-based subsidiary with U.S. approval. If an American wants to open an account in Basel, this is not possible. We refer him to this SEC-licensed subsidiary." (Bank8X; BaslerZeitung11/05/09, *emphasis added*)

OA717 Bank86: "Beginning in 2008, after the UBS scandal broke, [Bank86] initiated *a series of "Exit Projects"* to identify Swiss accounts that had been opened for U.S. customers, and ask the customers to either disclose their accounts to the United States, or close them." (PSI, 20140226: 4, *emphasis added*)

OA718 "We want to know if the money we manage is taxed." (Bank86; DieWeltwoche12/03/09)

OA719 The bank "transferred all U.S. clients to a subsidiary, which is registered as an investment advisor with the SEC." (Interviewee, SchweizerBank20/03/09)

A4) Traditional Swiss private banks

OA720 Bank9: "The Bank also put in place initiatives aimed at facilitating the *regularization of its other U.S. clients* under the OVDP. These initiatives included direct contact with the Bank's clients to urge compliance, and efforts to assist the Bank's clients in completing the OVDP process." (SOF9, 2015, *emphasis added*)

OA721 Bank9: "In March 2009, the Bank became aware of the first IRS Voluntary Disclosure Program (the "OVDP"). The Bank liaised and worked with External Asset Managers to ensure that US clients who were not already compliant would enter the OVDP." (SOF9)

OA722 Bank12: “On September 23, 2009, [Bank12] updated the directive issued to its employees, requiring that *all existing U.S. clients provide a Form W-9* and *sign the Basic Agreement for U.S. Persons*. The directive required that the agreement be signed within a reasonable period, but by no later than the client's next visit to the Bank. (...) Taken as a whole, the policies that [Bank12] implemented in 2008 greatly reduced its U.S. business. Between August l, 2008 and March 2015, [Bank12] closed 76 of its 126 U.S. Related Accounts.” (SOF12, 2015, *emphasis added*)

OA723 Bank12: “On March 26, 2009, [Bank12] issued a directive to its employees, requiring that any U.S. individuals seeking to open an account comply with the following requirements (...) to provide a signed IRS Form W-9.” (SOF12, 2015)

OA724 Bank18: “Accordingly, in 2009, [Bank18] began implementing a policy requiring that all private banking accounts of individual U.S. taxpayers be closed. As the customers’ U.S. status became known, [Bank18] took steps to close the accounts. By early 2010, all [Bank18] employees were instructed not to open accounts for U.S. persons, call U.S. account holders, or invest for U.S. related taxpayers.” (SOF18, 2015)

OA725 Bank22: “In the ... September 16, 2008 memorandum... the Bank’s existing U.S. clients and U.S. beneficial owners be required to *sign Forms W9 or terminate* their relationship with the Bank.” (SOF22, *emphasis added*)

OA726 Bank35 “*obtained waivers of Swiss bank secrecy* from some of its former U.S. clients.” (SOF35, 2015, *emphasis added*)

OA727 Bank35: “In relation to preexisting relationships with U.S. clients, the 2009 Policy stated that the Bank would determine whether to continue or dissolve the client relationship on a case-by-case basis. During 2009 and 2010, the Bank decided on *a general exit of U.S. clients*. (...) The Bank closed 187 U.S. Related Accounts with a total of assets under management of $156 million since August 1, 2008.” (SOF35, 2015, *emphasis added*)

OA728 Bank37: “In 2009, as a result of the UBS investigation becoming public..., the Board of Directors decided that the Bank no longer had an interest in opening accounts for U.S. clients. Around this time, the Bank began to identify U.S. accounts... and attempted to find solutions to bring the accounts into compliance. In or about late 2009, the Bank began informing its U.S. customers that they should either provide *evidence of U.S. tax compliance or leave* for another bank.” (SOF37, *emphasis added*)

OA729 Bank52: “... in or around September 2008, [the bank] began requiring Forms W-9 for all new U.S. clients.... Following that review, the Executive Board recommended to the Board of Directors that [the bank] exit its business with all U.S. residents, whether or not tax compliant, as well as U.S. persons living outside the U.S. who would not provide [the bank] with a *Form W-9* (the "*U.S. Exit*").” (SOF52, *emphasis added*)

OA730 Bank52: “Following that review, the Executive Board recommended to the Board of Directors that [Bank52] exit its business with all U.S. residents, whether or not tax compliant, as well as U.S. persons living outside the U.S. who would not provide [Bank52] with a *Form W9* (the "*U.S. Exit*").” (SOF52, 2015, *emphasis added*)

OA731 Bank56: “In August 2009, the Executive Board directed that new accounts with a U.S. beneficial owner would require an executed IRS Form W-9 at account opening and that any existing accounts with a U.S. beneficial owner refusing *to provide a Form W-9* would be required to leave the Bank by December 31, 2009.” (SOF56, *emphasis added*).

OA732 Bank76: “In 2009, the Bank decided to make a first exit from its existing relationships with U.S. clients that presented the greatest risk of non-compliance with U.S. tax obligations. At this time, the Bank imposed a W-*9 requirement for U.S. beneficial owners*.” (SOF76, *emphasis added*).

OA733 Bank80’s “Management Committee put in place a special policy for such accounts requiring the provision of *IRS Forms W-9* and waivers of bank secrecy.” (SOF80, 2015, *emphasis added*)

OA734 Bank80’s “Management Committee adopted a policy of *only servicing U.S. taxpayer-clients in full compliance with U.S. tax and securities laws* (...) Following the adoption of this policy, the Management Committee commissioned a feasibility study on the establishment of an SEC-registered subsidiary to provide investment advisory services to U.S. taxpayer-clients. In July 2011, [Bank80] Advisors was registered as an investment adviser with the SEC under Section 203(c)(2)(A) of the Investment Advisers Act of 1940.” (SOF80; 2015, *emphasis added*)

OA735 [Bank80’s] “Management Committee adopted a policy of *only servicing U.S. taxpayer-clients in full compliance* with U.S. tax and securities laws in 2010.” (SOF80, *emphasis added*)

OA736 Bank93: “In or about March 2009, Bank93 instituted an "Exit Policy" for U.S.-related accounts, including accounts of non-U.S. entities with U.S. beneficial owners. Under this policy, all such accounts were required to *provide Forms W-9* or face closure by specified deadlines.” (SOF93, *emphasis added*)

LOCALS

UNPROSECUTED ORGANIZATIONS:

COMPLIANCE WITH FOREIGN RULES

Noncompliance

<u>*Noncompliance*</u>

OA737 Wegelin: “Wegelin's conduct is all the more egregious because Wegelin *was undeterred by the investigation and prosecution of UBS*, another, albeit much larger, *bank that engaged in similar conduct*. At not one, but at two crucial junctures during the conspiracy, *Wegelin was undeterred*. When the Department of Justice's investigation of UBS for helping U.S. taxpayers evade taxes became widely publicized in May 2008, *Wegelin decided to welcome U.S. taxpayers and to profit from the UBS investigation, rather than see UBS’ experience as an example to be avoided*. And, worse, when UBS was actually charged with committing a crime under U.S. law, admitted doing so (...) *Wegelin kept assisting U.S. taxpayers in evading taxes* and did so for a significant period of time. *Wegelin’s choice to ignore the message of deterrence to be sent by the investigation and prosecution of UBS* is all the more egregious when

compared with some other Swiss banks. Some, although by no means all, other Swiss banks, did, in fact, exit the business of providing U.S. taxpayers with services designed to help them evade taxes after the UBS investigation became public...” (DOJ130225, 2013: 13, *emphasis added*)

OA738 Wegelin: “The disclosure of the UBS investigation presented Wegelin with a stark choice: *Either Wegelin could have been deterred by the Department of Justice's tax-related investigation of UBS and concluded that it should exit the business of assisting U.S. taxpayers in evading taxes*. Or, Wegelin could have viewed the steady outflow from UBS of customers as an opportunity to obtain assets under management ("AUM"), market share in the private wealth management business, and ultimately profits. Wegelin chose the latter course and chose to view the investigation of UBS and the resulting exodus of UBS customers as a business opportunity, rather than as an example to be avoided. This fact, by itself, strongly evidences Wegelin's willfulness. *The message of deterrence* generated by the fact that one of the largest and most prominent Swiss banks was being investigated for helping U.S. taxpayers evade their tax obligations simply *was not received by Wegelin* .” (DOJ130225, 2013: 5, *emphasis added*)

OA739 Wegelin: “Among other things, in 2008 and 2009, Wegelin, through its employees, opened and serviced dozens of undeclared accounts for U.S. taxpayers in an effort to capture clients lost by UBS in the wake of widespread news reports that the IRS was investigating UBS for helping U.S. taxpayers evade taxes and hide assets in Swiss bank accounts. Indictment ¶ 16; Compl. ¶¶ 30. By mid-2008, UBS had stopped servicing undeclared accounts for U.S. taxpayers. 15. In the wake of the IRS investigation, members of Wegelin's senior management affirmatively decided to capture the illegal business that UBS exited. Indictment ¶ 12; Compl. ¶ 29. To capitalize on the business opportunity this presented and to increase the assets under management, along with the fees earned from managing those assets, individuals acting on behalf of Wegelin told various U.S. taxpayer-clients that their undeclared accounts would not be disclosed to the United States authorities because the bank had a long tradition of secrecy. Indictment ¶¶ 13-14; Compl. ¶¶ 30-32. They also persuaded U.S. taxpayer-clients to transfer assets from UBS to Wegelin by emphasizing, among other things, that unlike UBS, Wegelin did not have offices outside of Switzerland and was therefore less vulnerable to United States law enforcement pressure. Id. 16. Members of Wegelin's senior management approved efforts to capture the clients who were leaving UBS and also participated in some meetings with U.S. taxpayer-clients who were fleeing UBS. Id. Wegelin's Zurich branch even developed a special code - BNQ - for new undeclared accounts, indicating internally within Wegelin, among other things, that the accounts were undeclared. Indictment ¶ 23; Compl. ¶ 34. According to the Indictment and Verified Complaint, Wegelin increased its U.S. business from approximately $240 million in undeclared U.S. taxpayer assets in 2005 to at least $1.2 billion in such assets in 2010. Indictment ¶ 26; Compl. ¶ 14.” (IRS; Declaration of CherylR.Kiger; 2013: 6-15)

OA740 Bank Wegelin's guilty plea exhibited that it "-Took in clients leaving UBS following disclosure of investigation of UBS and after UBS deferred prosecution agreement in early 2009"; "-UBS was aware of this and client advisors at UBS referred clients who had to leave UBS to Wegelin"; "Wegelin could charge high fees to new U.S. clients because clients were afraid of criminal prosecution in the U.S."; "-Created a procedure to accommodate UBS walk-in clients." (Wegelin's Guilty Plea; DOJ; Levy; 2013: 10)

OA741 “Significance of illegal conduct for Wegelin: -Growth in Undeclared AUM: $88 million in 2002 => $214 million in 2005 => $1.5 billion in 2009; -Growth in Undeclared Accounts: 89 in 2002 => 195 in 2005 => 684 in 2009; -Growth in Undeclared AUM as % of Total AUM: 2.3% in 2002 => 2.5% in 2005 => 7% in 2009; -Growth in Fees Derived Undeclared Accounts: $479K in 2002 => $769K in 2005 => $4.6MM in 2009.” (Levy, 2013: 12).

OA742 “How much new money were you able to attract? Interviewee: In the 2nd quarter about 1.2 billion Fr. What kind of customers are they? Interviewee: In the private banking sector, they are former customers of big banks, but also of smaller regional banks.” (Interviewee; HandelsZeitung12/08/2009)

OA743 “They have benefited from the UBS crisis. There has been an increasing number of customers who have deliberately searched for a smaller, independent banking institution.” (Interviewee; DerBund20/01/2010)

OA744 “Bank Wegelin's approach has been a popular practice in Swiss banking circles.” (HandelsZeitung17/01/13, *emphasis added*)

OA745 Bank1: “In limited instances, [Bank1] provided services to its U.S. clients whereby it structured a U.S. related account, so that it appeared as if it was held by a non-U.S. legal structure, such as an offshore corporation or trust, which aided and abetted the clients’ ability to conceal their undeclared accounts from the IRS. Approximately 12% of [Bank1]’s U.S. client accounts after August 2008 were held in the name of offshore structures. (...) [Bank1] has held and managed approximately 3,500 U.S. client accounts, which included both declared and undeclared accounts, with a peak AuM of $2.78 billion since August of 2008.” (SOF1; 2015)

OA746 Bank2 “saw an economic expansion opportunity (...) In the first phase of these marketing efforts, between April 2008 and February 2009, [Bank2] contacted a total of 21 external asset managers and one family office in Liechtenstein in an attempt to grow the cross-border business. [Bank2] was aware that U.S. taxpayers had a legal duty to report to the IRS, and pay taxes on the basis of all their income, including income earned in accounts that these U.S. taxpayers maintained at [Bank2]. Despite being aware of this legal duty, the Bank opened and maintained undeclared accounts for these U.S. taxpayers. (...) In sum, [Bank2]'s 2008 marketing efforts directly led to relationships that attracted 45 U.S. Related Accounts to the bank. As of August 1, 2008, prior to the expansion of its private banking business, [Bank2] had only two U.S. Related Accounts, with aggregate assets of approximately $100,000. But during the Applicable Period, the Bank opened more than 70 U.S. Related Accounts, approximately half of them held by non-U.S. entities such as foundations, trusts, and corporations.” (SOF2, 2015)

OA747 Bank3: “RMs in the U.S. cross-border business actively assisted or otherwise facilitated thousands of U.S. individual taxpayers in establishing and maintaining undeclared accounts in a manner designed to conceal the U.S. taxpayers' ownership or beneficial interest in said accounts. (...) As of November 2008, based on internal reporting at the time, ten percent of [Bank3's] U.S. clients, representing assets under management of $25.2 million, had signed an IRS Form W-9, and the remaining 90 percent, with assets under management of $129.3 million, had not.” (SOF3; 2015)

OA748 “As of March 2009, based on internal reporting at the time, [Bank3] had at least 16 U.S. ex-UBS clients, three of whom were introduced by external asset managers.” (SOF3, 2015)

OA749 Bank11 “open[ed] approximately 22 accounts for account holders exited from other Swiss banks and accepting deposits of funds from accounts at the banks from which the account holders exited.” (SOF11 , 2015)

OA750 Bank13: “U.S. persons opened 32 U.S. Related Accounts at [Bank13] after August 1, 2008. Only one of those persons provided [Bank13] with a Form W-9 upon opening an account. In most cases, the U.S. persons who opened accounts at [Bank13] after August 1, 2008, had been required to close their accounts at other Swiss banks. [Bank13] knew or had reason to know that most of these [Bank13] accounts were likely not declared to the IRS. (...) As of December 2011, the Bank’s total volume of business with U.S.-domiciled persons was more than three times higher than its total volume of business with persons domiciled in any other single country besides Switzerland.” (SOF13; 2015).

OA751 Bank14: “From August 1, 2008, through December 31, 2014 (the "Applicable Period"), [SOF14, 2015] provided private banking services for 90 "U.S. Related Accounts" (...) The highest collective value of these accounts during any month in the Applicable Period was approximately $65 million. Thirty-seven of these accounts were opened after August I, 2008.” (SOF14, 2015)

OA752 Bank14: “Many accounts that U.S. persons opened at [Bank14] were not, in fact, timely declared on Forms 1040 or FBARs; in some cases for multiple years.” (SOF14, 2015)

OA753 Bank15: “[L]ike other Swiss banks, [Bank15] was prohibited by *Swiss law* from disclosing the identity of an account holder. (...) Despite understanding that U.S. taxpayers had a legal duty to report to the IRS and to pay taxes on income earned in accounts maintained in Switzerland, the Bank failed to ensure that its U.S. customers were abiding by that duty when it accepted them as customers and failed to monitor and/or investigate the compliance status of U.S. clients while their accounts were at the bank and to otherwise ensure that U.S. clients became tax compliant.” (SOF15, 2015, *emphasis added*)

OA754 Bank19: “After it became public that the U.S. Department of Justice was investigating the conduct of UBS, and later other Swiss banks, [Bank19] allowed several U.S. persons who closed their accounts at Category 1 banks to open accounts at [Bank20] and transfer funds into those [Bank19] accounts from the Category 1 banks.” (SOF19, 2015)

OA755 Bank20 stated that “new U.S. clients would be accepted only under "exceptional circumstances." The bank accepted an account from an individual who was a foreign national and U.S. resident and who brought the account from UBS. At the time it approved the account, the Bank was aware that the client left UBS because he was concerned about the U.S. government's activities in investigating U.S. persons with accounts at that bank.” (SOF20, 2015)

OA756 Bank21: “In December 2008, [Bank21]’s board of directors decided that it should continue to manage U.S. clients and that it would also continue to open new accounts for U.S. clients on the condition that they had a "link to our region or one of our relationship managers." As a result, the Bank opened accounts for approximately ten U.S. taxpayers who transferred accounts from other Swiss institutions that were closing such accounts. The Bank knew, or had reason to know, that two of those accounts were undeclared.” (SOF21; 2015)

OA757 Bank29 “was aware that U.S. taxpayers had a legal duty to report to the IRS, and pay taxes on the basis of all their income, including income earned in accounts that these U.S. taxpayers maintained at [Bank29]. Despite being aware of this legal duty, the Bank opened and maintained undeclared accounts for some of these U.S. taxpayers.” (SOF29, 2015)

OA758 Bank31: “Prior to 2011, the Bank's relationship managers were not instructed to, and *did not evaluate or screen incoming U.S. clients for U.S. tax compliance status.*” (SOF31, 2015, *emphasis added*)

OA759 Bank32: “Of the 96 U.S. Related Accounts, 77 were opened before August 2008.” (SOF32, 2015)

OA760 Bank32: “[O]ne was opened between August 1, 2008 and February 28, 2009, and 18 were opened after February 28, 2009. (...) In two instances, [Bank32] accepted clients who left UBS as account holders. For example, one account holder opened an account in November 2008.” (SOF32, 2015)

OA761 Bank34 “did not structure, operate, or supervise its U.S. Related Accounts in any way that was different or separate from its non-U.S. Related Accounts. (...) U.S. Related Accounts were not separately managed at [Bank34]. For example, U.S. Related Accounts were not assigned to a particular group of relationship managers or client advisers.” (SOF34, 2015)

OA762 Bank34: “Of the 330 U.S. Related Accounts: (...) 21 were opened between August 1, 2008 and February 28, 2009; and 43 were opened after February 28, 2009. (...) [The] bank accepted funds from 19 UBS account holders who exited UBS.” (SOF34, 2015)

OA763 Bank38: “From August 1, 2008, through December 31, 2014 (the "Applicable Period"), [Bank38] provided banking services for 898 "U.S. Related Accounts," as defined under the Swiss Bank Program, with over USD273 million in assets. (...) Bank accepted 37 new U.S. Related Accounts in the remainder of 2009. Of these, 17 were funded by transfers from Category I banks. (...) 39 new U.S. Related Accounts in 2010.” (SOF38, 2015)

OA764 Bank47: “During the Applicable Period, the Bank opened and maintained at least 21 undeclared accounts in the names of structures that were beneficially owned by U.S. taxpayers, while knowing, or having reason to know that, these structures were used by U.S. clients to help conceal their identities from the IRS. (...) [Bank47] also offered a variety of traditional Swiss banking services that it knew could assist, and did in fact assist, U.S. clients in the concealment of assets and income from the IRS. One such service was hold mail. (...) [It] accepted instructions in connection with at least 22 U.S. Related Accounts ($7.9 million) (...) not to disclose the names of U.S. clients to U.S. tax authorities, including the IRS.” (SOF47; 2015)

OA765 Bank48 “opened new U.S. Related Accounts after December 2009.” (SOF48, 2015)

OA766 Bank54: “In late 2009 and early 2010, [Bank54] followed the instructions of two external asset managers concerning four separate U.S. Related Accounts that were directly held by U.S. taxpayer clients of [Bank54] to restructure the assets of the accounts into new

insurance-policy accounts, which were titled in the name of a Liechtenstein insurance company for the benefit of the same, underlying U.S. taxpayer clients with the same, underlying assets (so-called "insurance wrapper" accounts) (...) a former [Bank54] employee suggested to the external asset manager (by way of an unaffiliated third-party advisor) taxpayer clients with the same, underlying assets (so-called "insurance wrapper" accounts) (...) a former [Bank54] employee suggested to the external asset manager (by way of an unaffiliated third-party advisor) that use of such insurance-policy accounts could be an option for U.S. taxpayers who were being forced to exit their Swiss banking relationships. Restructuring the form in which their assets were held at [Bank54] Switzerland allowed these U.S. taxpayers to further hide their identities and undeclared accounts from the IRS and United States law enforcement." (SOF54; 2015)

OA767 Bank58: "Manager# 1 was asked in March 2008 by a [Bank58] private banker what could be offered to a U.S. couple residing in Mississippi who wanted to open two accounts for $1 million each, and the response given by Manager#1 was: [i]f they're declared, they can open in their name and sign W9. If not, suggest they use a pic [private investment company]. (...) When one member of [Bank58's] management inquired of another management member in February 2009 (...) The concerned member of management was then informed that interest earned on fiduciaries for example are not US reportable income and therefore do not appear on the 1099. (...) In September of 2009, a member of [Bank58's] management said: [private bankers] comforted clients by telling them that the Bank will not declare anything systematically to the IRS." (SOF58, 2015)

OA768 Bank58: "In the Applicable Period, [Bank] held a total of 919 U.S. Related Accounts, which included both declared and undeclared accounts, with an aggregate peak of approximately $1.58 billion in assets under management. All of these 919 U.S. Related Accounts had U.S. account holders or U.S. beneficial owners. As of August 1, 2008, [Bank58] had 579 U.S. Related Accounts, with an aggregate peak asset value of approximately $1.01 billion. During the Applicable Period, the Bank opened 340 additional U.S. Related Accounts, with an aggregate peak asset value of approximately $570 million. Of [Bank's] 919 U.S. Related Accounts, approximately 12 percent with an aggregate peak value of approximately $293 million held U.S. securities and were timely disclosed to the Internal Revenue Service through Form 1099 reporting." (SOF58, 2015)

OA769 Bank66: "In 2008, [Bank66] accepted the transfer of the account of one client from UBS. In opening the account, [Bank66] ignored its origin and an indication that the account proceeds may have been a firm of untaxed accounts." (SOF66, 2015)

OA770 Bank72: "[B]etween August 2008 and February 2009, the Bank opened 265 new U.S. taxpayer accounts, comprising an aggregate of $171 million in new assets under management (but which represented less than 3% of the additional new accounts opened in 2008), without determining whether the relevant U.S. taxpayer clients were tax compliant in the United States. After considering the warning of the Bank's Head of Internal Auditing and the legal department memorandum, the Bank nonetheless opened 30 U.S. Related Accounts from UBS. The Bank now recognizes that the policies it implemented in 2008 were insufficient, because nearly half of those were undeclared accounts." (SOF72, 2015)

OA771 Bank75: "U.S. clients "continue to find their way to [Bank75] by being referred via personal contacts of the [Bank] client advisers. (...) The Bank opened 222 new U.S. Related Accounts with maximum aggregate assets under management of approximately $106 million between January 1 and October 31, 2009. Prior to that period, the Bank had approximately 110 U.S. Related Accounts with maximum aggregate assets under management of $133 million. (...) In an email dated November 12, 2009, the Credit Suisse employee advised: "I have a USA customer, domicile New York, assets USD 694,000, typical American, born in 1963, very nice .... He has no other bank accounts but would be glad if we could broker an account for him but he will not be in Switzerland any time soon." (...) The response was "Thank you, we will gladly accept the customer. As a bank, I again recommend the [Bank75]. We will be happy to send the account opening documents to the USA, but we would rather not send the asset management contract. It would be better if the customer traveled to Switzerland for that." (...) In May 2009, the Bank began accepting customers from Credit Suisse who had either terminated their relationship with Credit Suisse or whom Credit Suisse had terminated. The first such customer came to [Bank75] by a chance meeting with a Bank relationship manager. That customer transferred an investment portfolio of less than $250,000 from Credit Suisse which necessitated communication between the Bank and Credit Suisse in order to transfer the securities to the Bank. In or about June or July 2009, a Credit Suisse employee contacted [Bank75] and, thereafter, two of the Bank's employees met with a Credit Suisse relationship manager in the Zurich branch of Credit Suisse. This Credit Suisse relationship manager explained that Credit Suisse needed to terminate a significant number of U.S. accounts and that he knew (...) that [Bank75] was accepting U.S. customers. At this meeting, one or more of the Bank's employees provided the Credit Suisse relationship manager with several business cards. (...) Credit Suisse employees may have directed departing customers to the Bank by providing to those customers some of those business cards or copies thereof. Credit Suisse employees also occasionally walked its customers, generally elderly customers, to the Bank for purposes of opening a new account at the Bank. (...) Additional customers came to the Bank as walk-ins." (SOF75, 2015)

OA772 Bank75: "The February 2010 Cross-Border Handbook noted, "The bank has the following aims" listing first, "The bank wants-in the sense of a side-business [to] start business relations with U.S. customers."" (SOF75, 2015)

OA773 Bank77 "opened and maintained [accounts] (...) belonging to U.S. taxpayers who had left other banks being investigated by the U.S. Department of Justice *without ensuring that each such account was compliant with U.S. tax law* from their inception (...) Because *Swiss law* requires [Bank77] to identify the true beneficial owner of structures on a document called a Form A, it knew that these were U.S. client accounts." (SOF77, 2015, *emphasis added*)

OA774 Bank78: "[T]he senior management of [Bank78] *viewed the exit of U.S. clients by the targeted Swiss banks as a business opportunity to be seized immediately rather than a warning to be heeded*. Other than the 83 U.S. Related Accounts (...) [the bank] opened at least 275 additional U.S. Related Accounts since August 2008, and at least 46 of those 275 U.S. Related Accounts were undeclared at account opening. Additionally, internal bank notes indicate that in September and October 2008 certain external asset managers with whom [Bank78] entered into agreements were expected to have *"many former UBS clients" and would introduce U.S. clients to the*

Bank. [Bank78] opened and maintained at least 117 accounts (with an aggregate peak asset value of approximately $185 million) belonging to U.S. taxpayers who had left other banks being investigated by the U.S. Department of Justice without ensuring that each such account was compliant with U.S. tax law from its inception at [Bank78]. (...) senior management did, however, solicit and approve two pipelines of new undeclared U.S. client accounts. (...) [Bank78] EB #1, noting that the opportunity to transfer U.S. clients from UBS was "*unanimously agreed to and specifically recorded*" during "the management meeting of 22 July [2008]." (...) Beginning in September 2008, for each account opened in this pipeline, *an exception form to the W-9 policy was signed by senior executives*, including in one case by CEO #2 (the new chief executive officer of [Bank78]). These exception forms *expressly stated that the account opening was for a U.S. client without a Form W-9 and noted that the assets were undeclared*. (...) On September 30, 2008, CEO #2 inquired (...): "How are your undeclared U.S. new clients treated? Are you 'pragmatic' and accept such?" (...) In my opinion, *we proceed until further notice in a 'pragmatic' way, that is, we take extra business 'on board'*. Your opinion?" [Bank78] EB #1 responded on October 1, 2008: "With current knowledge, I am clearly of the opinion *we should play this niche.*" (...) on January 27, 2009, [Bank78] EB #1 proposed to the Executive Board in an email that *the Bank "continue this pragmatic cooperation with [UBS Banker #1]* without amount or time limitation" and explained that "[UBS Banker #1] has introduced 7 US clients thus far. Approximately CHF 7.5 million have been received for these so far and advised transfers of CHF 3.5 million are still pending. The further potential for this type of client is still about 1-2 times more than we already have. In addition, [UBS Banker #1] will retire in June 2009 and will no longer operate client transfers or acquisitions." In responses sent to this message on the same day, *the [Bank78] general counsel and CEO #2 approved the recommendation.*" (SOF78, 2015, *emphasis added*)

OA775 Bank8X, in 2009, "still accepted funds from U.S. customers who did not declare their accounts. (...) [Bank81] took over many customers from UBS in the context of the UBS crisis, namely several thousands (...) including of course Americans. (...) Looking back, I have to say: It would have been smarter to have closed the door earlier for the Americans." (Interviewee; Bank8X; BaslerZeitung14/09/11)

OA776 "Many banks have benefited from the move away from UBS." (Interviewee; Bank8X; Reuters24/07/2009)

OA777 Bank8X "is one of those banks which took over American clients from UBS after 2008. According to lawsuits filed by the U.S., the bank tripled its U.S. customers until 2012." (InsideParadeplatz)

OA778 Bank95: "[The bank] branches near the airport were full of Americans after the UBS story." (Int. 62)

OA779 "There were two groups, and both also had this conviction that as long as we concentrate on Switzerland, we are safe. One group expanded the American business - okay, let's make business from it. And the others, they just held back a little bit here, but still trusted that the Swiss legal system would protect them." (Int. 64)

OA780 "UBS then inevitably threw out its undeclared US clients. And there were then, unfortunately, some banks that quickly rushed to pick up the nuclear waste that UBS had thus deposited on Paradeplatz." (Int. 69)

<u>*Avoiding US nexus/US jurisdictional means*</u>

OA781 "And from these came pretty much just this conclusion then, that one said, of course, one must not travel to the United States, one must not write and telephone to the U.S." (Compliance Consultant of several local Swiss banks) (Int. 71)

OA782 "Several banks are *banning travel* for their customer advisors." (Interviewee; Handelszeitung15/04/2009, *emphasis added*)

OA783 "Now, they [the U.S. regulators] focus on the criminalization of the [Swiss] bank and its employees to access account information and thereby tax evaders. For this reason, he has strictly forbidden his employees to *travel* to certain countries. The U.S. are naturally on the list." (Interviewee; Weltwoche20/08/09, *emphasis added*)

OA784 Bank11: "After the Department of Justice's investigation of UBS became public in May 2008, the [Bank11 realized U.S. nexus with] clients domiciled in the United States [that are] acquired on the basis of *correspondence.*" (SOF11, 2015, *emphasis added*)

OA785 Bank 8X: "*no U.S. travels, no advice to U.S. clients in the U.S.*" (Int. 13, *emphasis added*)

OA786 Bank "Wegelin - which had only minimal contact with U.S. clients in the United States and which implemented directives to prohibit *use of U.S. jurisdictional means*." (WegelinReply: 21, *emphasis added*)

OA787 "The worst is yet to come. For this reason, he has strictly *forbidden his employees to travel to certain countries*. The U.S. are naturally on the list. Most banks have imposed similar *travel restrictions on their employees.*" (Interviewee; Weltwoche20/08/09, *emphasis added*)

OA788 Bank1: "In July of 2008, [Bank1] adopted restrictions regarding business *travel to the United States* for members of management and RMs with more than ten U.S. taxpayer clients. (...) RMs met with U.S. clients *outside of the United States* to provide banking services and investment advice related to their undeclared accounts. (...) During late 2008 and 2009, it issued a series of policies that had the practical effect of: (...) insulating the bank's exposure for undeclared U.S. client accounts behind its contractual relationships with EAMs in spite of, in some cases, their connections to Category I banks. Although these policies did remediate some of its undeclared U.S. client accounts, they also allowed [Bank1's] RMs to continue to open and maintain many highly profitable undeclared U.S. client accounts. (...) cutting off the *paper trail back to the United States* for accounts not reportable by the bank to the IRS." (SOF1; 2015, *emphasis added*)

OA789 Bank3: "In August 2009, [Bank3's] management board amended [Bank3's] policy toward U.S.-related accounts. It prohibited all *travel to the United States* by [Bank3's] personnel." (SOF3, 2015, *emphasis added*)

OA790 Bank7 ensured that it did not "require registration with the SEC as an investment adviser." (SOF7, 2015)

OA791 Bank7: "After learning of the UBS AG (UBS) investigation, [Bank7] reviewed and enhanced its policies and procedures with respect to [U.S. laws]. Among other things. it limited the manner in which U.S. persons who had not provided [Bank7] with a Form W-9

could be serviced, including: (a) *not issuing debit or credit cards*; (b) not accepting orders or instructions by *telephone or facsimile from the United States*; and (c) *not communicating by telephone, facsimile, or e-mail with the United States*. The purpose of this limitation was to ensure that [Bank7] did not violate U.S. Securities and Exchange Commission ("SEC") rules that require registration with the SEC as an investment adviser." (SOF7, 2015, *emphasis added*)

OA792 Bank14: "Following a unanimous motion by the Bank's executive board, in October 2009 the board of directors voted to (...) continue the account relationships with clients of the External Asset Manager (including his U.S. clients) with the conditions that his business *be relocated to Switzerland (...*) U.S. persons could use the system to authorize securities transactions only if they executed those transactions at *computers located in Switzerland* (...) [U.S. persons had to] keep *evidence of their accounts outside of the United States* (...) During the Applicable Period, there was *no travel by Bank employees or managers to the United States* or elsewhere for the purpose of meeting with U.S. clients. (...) the situation could be "considered problem-free to a large extent *if the activities are performed in Switzerland.*" (...) [C]lients could only enter into account relationships with the Bank by visiting the Bank's offices *in Switzerland*. (...) he could *not visit any clients outside of Switzerland* for any business purpose. (...) could *not recruit new customers* abroad or visit existing customers abroad and that new accounts generally had to be created *in Switzerland.*" (SOF14, 2015, *emphasis added*)

OA793 Bank19 "had an informal policy *not to pursue and/or solicit clients outside of Switzerland* or even outside of the canton (...) permitted accounts to be held by Swiss or, in one case, foreign non-operating entities that were ultimately beneficially owned by U.S. persons. (...) hold bank statements and other mail relating to accounts at [Bank] rather than send them to U.S. taxpayers located in the United States, thereby ensuring that documents reflecting the existence of the accounts remained *outside the United States and beyond the reach of U.S. tax authorities* (...) in-person visits to [Bank] rather than by telephone or otherwise from the United States." (SOF19, 2015, *emphasis added*)

OA794 Bank20: "With respect to current U.S.-domiciled customers, the new policy *prohibited regular mail, email, or e-banking services."* (SOF20, 2015, *emphasis added*)

OA795 Bank21's "executive board in 2009 discussed that it must "process and clean up the subject area of US clients." (...) The 2009 cross-border Directive emphasized that business relationships could only be *initiated in Switzerland* and with the prospective account holder *physically present in the country*. The 2009 Directive, and a separately adopted Travel Directive, stated that relationship managers must refrain from engaging in any active acquisition of customers, including marketing or business activities with current or future clients if traveling abroad." (SOF21, 2015, *emphasis added*)

OA796 Bank21: A "procedural manual dated November 2009 related to the Directive warned its employees (...) "clients who do not want disclosure to the IRS (American tax authority) *may not be contacted at all in the U.S.A.* and/or other countries! Contact is only permissible *within (Switzerland)*. (...) [The bank] implemented a policy in 2009 with respect to foreign travel by its relationship managers. Pursuant to that policy, travel was permitted to the United States to meet with U.S. clients, so long, as it was approved in advance by the Bank's chief executive officer. But such *travel came with restrictions*. For example, under the policy, [Bank21] declared that "No files may be taken abroad," relationship managers must "complete a training course," relationship managers "may not actively acquire" new customers, there was to be "no signing of business documents" or "accepting of orders" or providing "investment advice," and bank employees were prohibited from "handing over cash, securities, or objects."" (SOF21, 2015, *emphasis added*)

OA797 Bank26: "On March 1, 2012, [Bank26] management issued a policy to personnel providing that the Bank would *no longer provide e-banking, e-mail contact, travel cash cards, or prepaid cards* for customers residing *in the United States*. Later that month, the Bank adopted standard language responding to requests from abroad, stating that 'as a local bank under Swiss law, we *do not give any advice abroad, neither by e-mail nor by phone*'." (SOF26; 2015, *emphasis added*)

OA798 Bank28: "In May 2009, [Bank28] began to formalize its policy with respect to U.S. clients by enacting a Code of Market Conduct which contains, among others, an express *prohibition for employees to prospect clients while in the United States*." (SOF28; 2015, *emphasis added*)

OA799 Bank29: "The December 2008 report to management, relationship managers, and the Central Register entitled "Banking Relations With U.S. Persons" stated that the Bank had an "*absolute ban from investment soliciting and/or advisory" with respect to U.S. persons*." (SOF29, 2015, *emphasis added*)

OA800 Bank29 "agreed to hold bank statements and other mail relating to the accounts at the Bank, rather than send them to U.S. taxpayers located in the United States, to ensure that documents reflecting the existence of the accounts *remained outside the United States and beyond the reach of U.S. tax authorities,* given Switzerland's bank secrecy laws." (SOF29; 2015: *emphasis added*)

OA801 Bank32: "[T]hrough its hold-mail service and at the request of clients, [Bank32] did not send periodic statements or communications to its clients and, instead, retained correspondence at [Bank32] for later client review, thus ensuring that documents reflecting the existence of the accounts *remained outside the United States, beyond the reach of U.S. tax authorities, and protected by Swiss banking secrecy laws.* Of the 96 U.S. Related Accounts, 23 had hold-mail service. Of the total [Bank32] client population, 1,500 accounts had hold-mail service during the Applicable Period." (SOF32; 2015, *emphasis added*)

OA802 Bank 34: From May 2009, [Bank34] sought to establish that prospective U.S. account holders had a close connection to Switzerland. (...) [They were] employed in Switzerland, owned property *in Switzerland* (e.g., a vacation home), were related to an existing account holder at [Bank34]." (SOF34, 2015, *emphasis added*)

OA803 Bank38: "On February 18, 2009, the U.S. Department of Justice and UBS filed a deferred prosecution agreement in the U.S. District Court for the Southern District of Florida (...) On the same day, the Swiss Financial Markets Supervisory Authority ("FINMA") also published a report on its investigation into the U.S. cross-border business of UBS. *These developments caused [Bank38] Bank to review its policies on communications with clients in the United States*. (...) In May 2009, [Bank38] Bank issued a directive (...) *prohibiting Bank employees from sending correspondence to the United States or accepting orders received by telephone, fax, or mail*

from the United States, and prohibiting U.S. domiciled clients from initiating transactions through the e-banking system." (SOF38, 2015, *emphasis added*)

OA804 Bank4X: "One has tried to reduce this so-called nexus to the U.S., so that one is in line with these licensing regulations and concentrates on this Swiss jurisdiction." (Int. 74)

OA805 Bank47: "In the spring of 2009, the Bank adopted additional precautions again at the direction of [Bank47]. [Bank47] banned written and telephonic communications with clients *in the United States*, and all e-banking contacts with U.S.-domiciled persons." (SOF47, 2015, *emphasis added*)

OA806 Bank58: "In July 2008, [Bank58] adopted *restrictions regarding business travel to the United States* for its private bankers that required preapproval from the department head. (...) At least four private bankers, however, did use personal email or coded language to correspond with undeclared U.S. clients. For example, a June 2009 email message was sent to an undeclared U.S. client from a [Bank58] private banker with the only content being a subject line that read "[]mail fish up 20%."" (SOF58, 2015, *emphasis added*)

OA807 Bank59 "agreed not to send any mail to 47 U.S.-resident clients. This ensured that documents acknowledging the existence of the accounts remained *outside of the United States and beyond the reach of U.S. tax authorities."* (SOF59; 2015, *emphasis added*)

OA808 Bank5X: "One does not write to the USA, does not travel to the U.S.A., and also didn't have customers domiciled in the States, in the USA." (Int. 57)

OA809 Bank60: "On February 18, 2009, the U.S. Department of Justice and UBS filed a deferred prosecution agreement in the U.S. District Court for the Southern District of Florida in which UBS admitted that its U.S. cross-border business used Swiss privacy law to aid and assist U.S. clients in opening and maintaining undeclared financial accounts. On the same day, the Swiss Financial Markets Supervisory Authority ("FINMA") also published a report on its investigation into the U.S. cross-border business of UBS. 45 *Following these events, [Bank60] reviewed its procedures to ensure that there was no investment advice or other communications with U.S. clients in the United States by so-called "jurisdictional means.*" (...) In March 2009, [Bank60's] executive board *prohibited Bank employees from contacting U.S.-resident clients by any means, including telephone, e-mail, and e-banking*." (SOF60, 2015, *emphasis added*)

OA810 Bank63: "Bank employees may not take any documents regarding the client relationship with them and may *not provide advice outside Switzerland*. Bank employees also may *not accept orders outside Switzerland*." (SOF63, 2015, *emphasis added*)

OA811 Bank64 "also assessed *whether U.S. law enforcement would be able to seize* [Bank64's] U.S. clearing account, which was held at UBS. (...) a senior partner and then head of compliance informed the attendees that the Bank had received a confirmation from UBS that *[the bank's] U.S. dollar correspondent bank account was "managed at UBS in Zurich and not in Stamford, [Connecticut] U.S.A*. Consequently, and in the worst case (...), the U.S. authorities would not be able to block any funds!"" (SOF64, 2015, *emphasis added*)

OA812 Bank72: "In September 2008, as a precaution against exited UBS clients, the Bank's legal department recommended to all employees by e-mail not to accept new United States clients unless the clients were physically present in *Switzerland* (...) In particular, the Bank's customer relationship managers *never traveled to the United States* to solicit clients or to provide clients with investment advice. *Contact by Bank personnel with potential clients in the U.S. was prohibited*. Since November 2009, clients domiciled in the United States were *denied access to e-banking facilities*. (...) The Bank also *prohibited its client relationship managers from making telephone calls or using other means of electronic communication between Switzerland and other countries, including the United States*. Further, the Bank terminated e-banking services for all existing and future clients living in the United States." (SOF72, 2015, *emphasis added*)

OA813 Bank 78: "In June 2009, [Bank78] adopted restrictions *prohibiting business travel to the United States* by its private bankers. (...) private bankers *met with U.S. clients outside of the United States* to provide banking services and investment advice related to their accounts, which included undeclared accounts. For example, one [Bank78] private banker regularly met with a U.S. client who resided in the United States and had assets of more than $90 million in an account at [Bank78] held by a Liechtenstein foundation, in a Swiss hotel, at the Bank, or in London, England. When meeting in London, the [Bank78] private banker usually delivered cash amounts of 10,000 to 50,000 Swiss francs or U.S. dollars to the U.S. client." (SOF78, 2015, *emphasis added*)

OA814 Bank9X: "*we are in Switzerland*, I'm doing it in Switzerland. I'm not soliciting in the US, right? I can go put a stool in front of UBS here and open accounts right here for people who come out of the bank. You didn't need to go to the US. They were here. The clients were here. They came physically to withdraw money." (Int. 87, *emphasis added*)

OA815 Bank9X: "No, it's not something that we're going to actively pursue. But if they will come, we will consider it." (Int. 87)

<u>*Banks with entrepreneurial mindset and growth strategies more likely to seize new arbitrage opportunities*</u>

OA816 Bank0X: "They have this entrepreneurial model, in which, basically, the individual bankers are almost like a small bank by itself." (Int. 82)

OA817 Bank0X: "Very strong own identity, with very big focus on growth." (Int. 81)

OA818 Bank3X: "The only cantonal bank that had a relatively aggressive offshore private bank." (Int. 54)

OA819 [Bank4X] "had a very strong growth strategy at the time." (Int. 54)

OA820 Bank5X: "The idea that Switzerland from a jurisdictional standpoint would prove to be robust was a factor, so we said "Oh, absolutely. You're an entrepreneurial banker. We want to be supportive of you doing this. You're really excited about an opportunity that's emanating in that particular segment. Go for it."" (Int. 60)

OA821 Bank5X: "they had a very aggressive approach to onboarding clients." (Int. 55)

OA822 Bank5X: "I'm absolutely not surprised that we probably kind of gained some additional exposure that wouldn't have been desired because of the entrepreneurial approach." (Int. 60)

OA823 Bank5X: "It's more to do with the sort of the entrepreneurial laissez-faire model. And therefore, there are bankers who ultimately would see this as an opportunity. And therefore, we're at Bank58 and we can go and grab that." (Int. 60)

OA824 Bank5X: "free-wheeling entrepreneurialism." (Int. 60)

OA825 Bank9X: "To take on these US customers as well, that was greed, that was *simply greed*... they made a business model out of it." (Int. 69, *emphasis added*)

EPISODE2

LOCALS

UNPROSECUTED ORGANIZATIONS:
COMPLIANCE WITH FOREIGN RULES

Compliance

OA826 "I would say, as of 2012, banks started cleaning their books, started getting rid of their non-compliant clients. (...) And, gradually if you wished, the books of the banks had to be cleaned up, and also banks started focusing on certain markets in the manner that is supposedly compliant with the legislation. And I think this is basically where we stand now. (...) Most, if not all banks, have had to refocus their books, so that led to some banks disposing of their books. For instance, [Bank0X] over the last five years has sold a large portion of its book that was in jurisdictions which they didn't want to cover anymore, and they focused instead on certain jurisdictions and they kept those books. So that's one example but many banks have actually done the same thing." (Int. 5)

OA827 "But the end was-- the period during which it happened was between the UBS case and the NPAs. And then it ended during the NPA saga. So, I went to Washington (...) all this stopped in 2013 or something like that." (Int. 11)

OA828 After the Wegelin case, "we were all the more vigilant to have the least possible, if not at all, *American customers. (...) We started to close them*." (Int. 2, *emphasis added*)

OA829 "[A]ll banks want to avoid further hardship with the U.S. and to deliver the information *required by the U.S. tax authorities.*" (Interviewee, HandelsZeitung10/03/10, *emphasis added*)

OA830 Bank3: "In November 2011, [Bank3] decided that it would maintain accounts for *U.S. clients* only if the accounts had been disclosed to the relevant U.S. authorities." (SOF3, 2015, *emphasis added*)

OA831 Bank3: "In December 2011, [Bank3] sent letters to its U.S. clients requesting a bank secrecy waiver and asking them to *confirm that their accounts had been disclosed to the relevant U.S. authorities*. Where the requested documentation was not provided, [Bank3] terminated the relationship." (SOF3, 2015, *emphasis added*)

OA832 Bank13: "at the request of [Bank13] management, the Bank's board of directors decided to discontinue the Bank's U.S. cross-border business. [Bank13] would not accept any more new accounts from U.S. persons and, with limited exceptions, would close *all existing accounts of U.S. persons by the end of that year*. [Bank13] informed its U.S. clients of this decision by letter in February 2012. In the letters, [Bank13] also requested that the departing U.S. clients provide *Forms W-9* to the Bank before exiting, and many of them did so. The process of closing U.S. Related Accounts was supervised by the Bank's board of directors and its management and was primarily implemented by the Bank's compliance officer and external compliance consultant." (SOF13, 2015, *emphasis added*)

OA833 Bank17 started "to encourage its U.S. clients to ensure *tax compliance in their home jurisdictions*. (...) the *observance of the law and rule of the local country*." (SOF17, 2015, *emphasis added*)

OA834 Bank20: "The Cross-Border Team began the process of closing U.S. Related Accounts in August 2013, when termination communications were sent to U.S. clients. In September 2013, the Bank's Compliance Group informed U.S.-domiciled clients that their accounts would be subject to a total suspension, and in October 2013 the Bank again communicated with U.S. customers who had not responded to the initial mailing." (SOF20, 2015)

OA835 Bank23, in 2011, implemented a policy stating "that U.S. client accounts could only be opened *with documentation of tax compliance" under U.S. law*. (SOF23, 2015, *emphasis added*)

OA836 Bank26: "The form also requested that the customer *authorize the Bank to disclose information to the Department of Justice and the Internal Revenue Service* and expressly *release the [Bank26] from the terms of the Swiss banking secrecy act* as set forth in the banking act." (SOF26, 2015, *emphasis added*)

OA837 Bank26: "On June 28, 2010, [Bank26's] Board of Directors approved the "tax declared money only strategy." Under this strategy, the account opening process would cease when doubts arose about a customer's tax compliance. Further, the strategy required that the Bank *close existing banking relationships within 12 months after discovering that the account holder was not tax compliant.*" (SOF26, 2015, *emphasis added*)

OA838 Bank28, in December 2011, "required relationship managers to contact U.S. clients domiciled outside Switzerland in order to *clarify their situation* and, if necessary, ask the client to leave the bank. (...) clients domiciled outside of Switzerland sign a cross-border certificate ("Cross-Border Certificate") *attesting that the assets deposited at [Bank28] have been declared to tax authorities in their home country*." (SOF28, 2015, *emphasis added*))

OA839 Bank28: "In December 2011, [Bank28] enacted a Cross-Border Circular which prohibits [Bank28] employees from prospecting clients domiciled outside of Switzerland and requires that *new clients domiciled outside of Switzerland* sign a cross-border certificate ("Cross-Border Certificate") attesting that the assets deposited at [Bank28] have been declared to tax authorities in their home country." (SOF28, 2015, *emphasis added*)

OA840 Bank29: "On November 6, 2013, the Bank sent a letter to clients (...) requesting documents be provided regarding the client's tax status by November 30, 2013, and recommending clients who have not yet declared their [Bank29] account information to the IRS to enter the Voluntary Disclosure Program (a link to the IRS Voluntary Disclosure Program web site was provided)." (SOF29, 2015)

OA841 Bank29's "objective was "assuring the total compliance *to the U.S.A. imposed restrictions* inherent to the banking relations with U.S. Persons and persons who are fiscal subjects in the U.S.A.""" (SOF29, 2015, *emphasis added*)

OA842 Bank32: "In or about late 2011, and pursuant to guidance provided by the Swiss Financial Market Supervisory Authority, [Bank32] created a cross-border team based in its (...) headquarters to help bank personnel properly *service account holders residing outside of Switzerland*." (SOF32, 2015, *emphasis added*)

OA843 Bank32: "*sought and obtained waivers of Swiss bank secrecy* from the holders of U.S. Related Accounts." (SOF32, 2015, *emphasis added*)

OA844 Bank41's position in August 2011: "[B]anking services may only be offered to international clients not resident in Switzerland in *compliance with regulations applicable wherever the account holder is resident.*" (SOF41, 2015, *emphasis added*)

OA845 Bank47 "provided information for *the Department and the IRS* to *make treaty requests to the Swiss competent authority for certain U.S. Related Accounts.*" (SOF47, 2015, *emphasis added*)

OA846 Bank61: The bank implemented "series of measures and reforms specifically intended to ensure that its clients complied with *their applicable U.S. tax and reporting obligations.*" (SOF61, *emphasis added*)

OA847 Bank63 "did not have any U.S. person policy until 2010, when it adopted a policy principally related to *compliance with U.S. Securities and Exchange Commission requirements, rather than U.S. tax compliance* more broadly." Thereafter, "the Bank has focused on collecting more information with respect to the tax compliance of its clients." (SOF60, 2015, *emphasis added*)

OA848 Bank66 "renounced its previous practice of accepting 'manifestly untaxed assets from foreign clients'". (SOF66, 2015)

OA849 Bank64: "then requested that all U.S. domiciled clients and entities with U.S. domiciled beneficial owners needed to (1) provide an applicable IRS form; (2) confirm in writing that the beneficial owners were in compliance with their individual reporting requirements; and (3) provide written consent to allow the Bank to report their accounts to the IRS." (SOF64, 2015)

OA850 Bank67 "Also during 2012, [Bank67] established a compliance team of more than 20 full-time employees dedicated exclusively to reviewing and approving all openings and closings of, and changes to, accounts of *U.S. citizens and resident aliens...*" (SOF67, 2015, *emphasis added*)

OA851 Bank72: "In February 2012, [Bank72] decided to *terminate its relationships with all United States clients*, including those clients domiciled in Switzerland." (SOF72, 2015, *emphasis added*)

OA852 Bank75: "In or about 2011, (...) the Bank began to require *all existing potential U.S. customers to execute a form entitled Determination of U.S. Tax Status of Natural Persons or Determination of U.S. Tax Status for Legal Entities and Companies*." (SOF75, 2015, *emphasis added*)

OA853 Bank75 implemented increasingly restrictive measures: "beginning in 2011, the Bank (...) would not provide investment services in the U.S. or correspond with customers in the U.S., and, for those U.S. customers subject to tax withholding, the Bank requires a Withholding Statement." (SOF75, 2015, *emphasis added*)

OA854 Bank77: "In June 2013, the Bank implemented more restrictive and effective measures concerning its U.S. cross-border business. In its account opening process adopted that month, a new client must confirm *tax compliance with* respect to its assets and income and affirm that it will continue to remain tax compliant." (SOF77, 2015, *emphasis added*)

OA855 Bank80's "Management Committee put in place a special policy for such accounts (...) only serving U.S. taxpayer-clients in full compliance with *U.S. tax and securities laws.*" (SOF80, 2015, *emphasis added*)

OA856 Bank8X: In February 2012, Bank8X's top management "*required for every U.S. client a confirmation of a tax lawyer, stating that the assets are declared*". The bankers of Bank8X, in a unique and special exercise with call center and night shifts, called numerous U.S. clients, who had to decide immediately upon making their accounts transparent to the U.S. tax office IRS. (Insideparadeplatz, *emphasis added*)

Launching W9 or exit programs

OA857 Bank1: "issued a series of effective and gradually restrictive policies that resulted in *exiting most of its undeclared U.S. client accounts by the end of 2012*. [Bank1] implemented increasingly restrictive and effective measures concerning its U.S. cross-border business. [Bank1] adopted a policy prohibiting the acceptance of any new accounts for U.S. taxpayers without a Form W-9... in *January of 2011*, [Bank1] adopted a policy requiring *the closure of existing U.S. client accounts that failed to provide a Form W-9* or evidence of participation in an IRS Offshore Voluntary Disclosure Program." (SOF1; 2015, *emphasis added*)

OA858 Bank2: "In February 2012, the Bank sent a letter to all accountholders with an identified U.S. nexus, requesting that they provide a *signed IRS Form W-9 by June 30, 2012*. The letter stated that, if the customer did not provide the requested documentation, the Bank would terminate the relationship." (SOF2, 2015, *emphasis added*)

OA859 Bank14: "In October 2013, (...) issued a formal statement of policies regarding its U.S. cross-border business. Regarding the closing of U.S. Related Accounts, the Bank prohibited [Bank14] employees from aiding and abetting current or former clients in concealing assets from U.S. tax authorities. The directive also allowed employees to open new U.S. Related Accounts only if they had a personal initial conversation with the potential client, received a *Form W-9,* and obtained approval by the Bank's executive board. The directive also stressed that, if there were any doubts as to whether a client was meeting his U.S. tax obligations, the Bank must request additional documentation demonstrating tax compliance." (SOF14, 2015, *emphasis added*))

OA860 Bank1X: "We had to regularize the U.S. customers so that we would not have a problem as a bank." (Int. 84)

OA861 Bank1X: “From then on, one demanded from the customers that they bring W9 or corresponding documents, then set grace periods et cetera, and then decided that those who were not willing to do that had to leave the bank.” (Int. 84)

OA862 Bank19: "[S]ince November, all U.S. clients have been required to *provide a Form W-9.*" (SOF19, 2015, *emphasis added*)

OA863 Bank21: "In February 2012, the Bank's management decided (...) *all existing U.S. clients to submit to the Bank a Form W-9*. Accounts of clients who failed to provide the form were closed." (SOF21, 2015, *emphasis added*)

OA864 Bank26: "On March 25, 2013, [Bank26's] management issued a policy requiring that *all existing customers living abroad* who have not *signed the Bank's "tax-confirmation" form* complete the form. (...) In September 2013, [Bank26] issued a series of policies concerning U.S. Related customers: a. The Bank adopted new forms for all new customers, asking *whether the customer is a U.S. related person*." (SOF26; 2015, *emphasis added*)

OA865 Bank27 “started to assess its existing U.S. customer base for information relating to tax compliance, specifically the existence of an *IRS Form W-9*. One week later, with a view to increasing its scrutiny of its U.S. customer accounts, [Bank27] replaced the March 2009 directive on U.S. customers with an instruction to all employees requiring that any new relationships with U.S. persons had to be reviewed and approved by the management.” (SOF27; 2015, *emphasis added*)

OA866 Bank31: “In 2011, [Bank31] implemented a project that it called "Colombo" to change the manner in which it handled U.S. clients. (...) Beginning in June 2011, the Bank sent *a letter to all U.S account holders requesting that they submit a Form W-9 to the Bank*. The bank further advised that unless it received documentation that an account was, or had become tax compliant by July 31, 2011, the account would be closed. (...) Relationship managers were reminded to actively pursue a signed Form W-9 from the Bank's U.S. clients or to close the clients account if one was not provided. The freezing of accounts was systematically implemented and some accounts were closed.” (SOF31; 2015, *emphasis added*)

OA867 Bank32: “In or about late 2011 (...) [Bank32] began to *require U.S. persons to close their accounts if they did not provide [Bank32] with a Form W-9*. New U.S. clients were required to sign a Form W-9.” (SOF32, 2015, *emphasis added*)

OA868 Bank34: “By April 2012, *all U.S. account holders were required to sign a Form W-9*, regardless of whether they held any securities. Beginning in summer 2012, [Bank34] began closing U.S. accounts without a signed Form W9 regardless of the assets held in the accounts.” (SOF34, 2015, *emphasis added*)

OA869 Bank38: “In April 2012, [Bank38] began sending letters to its U.S. clients asking them to *sign Forms W-9 and Swiss bank-secrecy waivers* (...) The Bank warned that it would block the accounts of clients who did not respond.” (SOF38, 2015, *emphasis added*)

OA870 Bank41: “Beginning in May 2012, the Bank began requesting that new and existing clients sign and have on file *a Form W-9 and a combination self-certification as to tax compliance* and waiver of [Bank41's] obligations under the Swiss banking secrecy laws. (...) In December 2012, [Bank41] further tightened its compliance policies with respect to external asset managers.” (SOF41, 2015, *emphasis added*)

OA871 Bank58: "Until June 2013, however, [Bank58] requested but did not require all of its U.S. clients to *provide a signed IRS Form W-9 and to confirm whether their accounts were disclosed to the IRS*." (SOF58, 2015, *emphasis added*)

OA872 Bank60: "In April 2011, [Bank60] executive board decided to maintain existing U.S. Related Accounts and accept new U.S. Related Accounts *only if the U.S. client provided the Bank with a signed Form W-9*. (...) In June 2011, [Bank60] sent letters to all of its U.S. clients who had not previously provided Forms W-9 and requested that they do so." (SOF60, 2015, *emphasis added*)

OA873 Bank60 "In April 2011, [Bank60's] executive board decided to maintain existing U.S. Related Accounts and accept new U.S. Related Accounts only if the U.S. client provided the Bank with a signed Form W-9." (SOF60, 2015)

OA874 Bank63: "adopted a program to *exit U.S. persons who did not have a properly signed Form W-9*. (...) In 2012, the Bank decided to provide resources, including staff assistance, to ensure the closure of these accounts by June 30, 2012. Although the exit process did not meet the June 30, 2012 deadline, 30 U.S. Related Accounts that were open in early 2012 and lacked a Form W-9 in the file were closed by the end of 2012, with another 17 closed in subsequent years." (SOF63, 2015, *emphasis added*)

OA875 Bank64: “In March 2012, the Bank initiated a review of U.S. client accounts to identify any clients who had not filed a Form W-9 (or W-8BEN or W-81MY as appropriate) in instances where a Form W-9 was not required by [U.S. law]. [Bank64] then *requested that all U.S. domiciled clients and entities with U.S. domiciled beneficial owners needed to: (1) provide an applicable IRS form*; (2) confirm in writing that the beneficial owners were in compliance with their individual reporting requirements; and (3) provide written consent to allow the Bank to report their accounts to the IRS (...) In September 2012, the Bank sent letters to all U.S.-domiciled clients requesting the provision of this documentation to the Bank by October 31, 2012. Clients who failed to provide the above documentation by the end of 2012 had to terminate their relationship with the Bank.” (SOF64, 2015, *emphasis added*)

OA876 Bank64 “must actively endeavor to determine the tax status of all US-domiciled clients by means of the *form W-9*.” (SOF64, 2015, *emphasis added*)

OA877 Bank6X: “As of 2011, they really required everyone to have this *W9 and waiver*.” (Int. 85, *emphasis added*)

OA878 Bank72 “required customers making such cash withdrawals to acknowledge *valid customs legislation* with regard to the import and export of currency to a third country. The Bank's form warned that: "If the undeclared currency is discovered during a check, *the competent authorities* will always be informed," and the Bank required its customers to sign a statement reading, "I confirm that I have been informed that cash withdrawals may be regarded *as acts of concealment in the field of money laundering or in the tax field* and that I alone remain responsible for *fulfilling my duties towards the tax authorities of my country of domicile.*" ...unless clients "provided a Form W-9 as *evidence of their complying with U.S. tax laws*, and-provided a written confirmation that the assets held at the Bank were disclosed to the IRS.” (SOF72, 2015, *emphasis added*)

OA879 Bank75: "started its *exit program in 2011*. (...) In or about mid-2011, the Bank began requiring new and existing U.S. Related Account holders to, inter alia, *complete a Form W-9.*" (SOF75, 2015, *emphasis added*)

OA880 Bank78: "The management board of [Bank78] decided that all U.S. clients were to submit a *Form W-9*." (SOF78, 2015, *emphasis added*)

OA881 Bank78: "In March 2010, the management board of [Bank78] decided that *all U.S. clients were to submit a Form W-9,* and if a U.S. client did not submit a Form W-9, the relationship would be terminated. This forced termination initially only applied to one category of U.S. clients: U.S. client relationships that had assets in excess of 250,000 Swiss francs and possessed no so-called "termination-aggravating products". These "termination-aggravating products" included, among others, structured products, safe-deposit boxes, fixed-term products and certain savings accounts. The management board of [bank] did not decide to launch this termination process until January 18, 2011, and the *termination process* for the second priority group, which consisted of the U.S. clients with the "termination-aggravating products," *did not begin until July 14, 2011*." (SOF78, 2015, *emphasis added*)

OA882 Bank79 "Executive #1 wrote that "*U.S. clients who have not signed a W9 form so far will be informed that they must sign such form or otherwise leave the bank.* "The minutes from the July 8, 2011 management board meeting state that 'the board discussed [Bank79's] need to come up with a strategy for eliminating U.S. accounts where the customer has not signed a Form W-9.'" (SOF79; 2015, *emphasis added*)

OA883 Bank9X: "From 2011 onwards, this was carried through. These clarifications of the US customers that had to be made, which were then, how shall I say, very systematically brought into the bank." (Int. 77)

REINFORCEMENT BY EPISTEMIC AUTHORITIES

EPISODE1 **REINFORCEMENT BY EPISTEMIC AUTHORITIES**

COSMO-POLITANS **Reinforcement of goal-based territorial categorizations**

Disagreement with authorities making an attribute-based territorial categorization

OA884 Bank4X: "This was this *hypocrisy of FINMA*... This solved a UBS problem, but created a much bigger new problem." (Int. 88, *emphasis added*)

OA885 Bank4X: "Their position, that was *perceived as 'exotic'*." (Int. 88, *emphasis added*)

OA886 Bank8X: "We said, it can't be that this doesn't affect us... We have seen that is problem for the whole industry." (Int. 56)

OA887 Bank8X: "These were not just any FINMA employees, these were the top management. And there I had, with our U.S. attorneys, I have a very bad feeling about this." (Int. 56)

OA888 Bank8X: "Yes, I must also say, so for me it was *a debacle* how that went down in Switzerland." (Int. 56, *emphasis added*)

OA889 "Boundaries do not apply to you. A banking group is not only an economic reality, but also a legal one. It is the group that must abide by the law. The group must be run in the same way everywhere. If there is a place where there is too much shenanigans, we will close that subsidiary of the group." (Int. 89)

OA890 "It was already clear, just those gentlemen who have really championed the bank customer secrecy even longer, they pushed back the course of the FINMA… if only already in 2009 a strict recommendation had come." (Int. 68)

OA891 "The way FINMA let this through after UBS, that they didn't warn, *we didn't understand that*, and we still don't understand that today." (Int. 68, *emphasis added*)

Reinforcement by internationally oriented advisors and networks

A1) Internationally oriented banking associations

OA892 Bank4X: "There was some information exchange through this *[association of international banks]*." (Int. 88, *emphasis added*)

OA893 "At the events, we also invited a *U.S. lawyer*... they also flew in people from their environment, *American lawyers*. Or lawyers with an American degree, who could then look at the problem." (Int. 86, *emphasis added*)

OA894 "The [association of international banks] has held many events. *U.S. law firms* also appeared there, which of course then also positioned themselves as advisors and consultants." (Int. 69, *emphasis added*)

OA895 "We did something [at the association of international banks] ... not just about the whole SEC licensing process. Because, the tax issue was really the surprise." (Int. 86)

OA896 "I've had several bankers who were closer to the Swiss banking Association, who said, we hear horrible things on the US after the UBS case... They said, we need to do something... The *Swiss banking association* was more sensitive to the US." (Int. 82, *emphasis added*)

OA897 The "Geneva circle was very reluctant at first. But then it moved much faster than the German-speaking part of Switzerland. They always insisted on sovereignty and on Switzerland." (Int. 79)

A2) International banks/bankers

OA898 Bank3X: "Our relationship with [CEO of a cosmopolitan bank and head of a cosmopolitan banking association] was very close, and we learned certain things there that other banks might not have learned so quickly. And we simply noticed that something was brewing." (Int. 75)

OA899 Bank5X: “We had very *close ties with UBS*. We were also able to make inquiries from our network. And I believe that in these confidential discussions, we were told that this whole story cannot or should not be taken lightly.” (Int. 68, *emphasis added*)

OA900 Bank5X: “I came from Bank [8X] on [date]. I was a *lawyer there with the head of compliance* at [a large cosmopolitan Swiss bank] and then became General Counsel here.” (Int. 68, *emphasis added*)

OA901 Bank5X: “Yes, because that was information, for example, that I really received very confidentially from UBS, that you should not believe as a bank that the U.S. does not know exactly where these customers are going.” (Int. 68)

B) Internationally oriented legal advisors

OA902 Bank 16 “engaged *U.S. and Swiss counsel as well as forensic accounting experts* to conduct an internal review in order to identify and collect data and information regarding its *U*.S.-taxpayer accounts and to examine its conduct in relation to such accounts.” (SOF16, 2015, *emphasis added*)

OA903 Bank35 “In July 2008, following reports of the UBS investigation and *after receiving legal advice from a U.S. law firm*, the Bank's executive board ("the Board") decided (i) not to accept U.S. nationals domiciled in the United States as new clients (with exceptions subject to specific approval), and (ii) to enter into new relationships with U.S. nationals domiciled outside the United States only if the relationship was approved by a member of the Board.” (SOF35, 2015, *emphasis added*)

OA904 Bank35: “Receiving legal advice from a *U.S. law firm*.” (SOF35, *emphasis added*)

OA905 Bank5X: “The lawyers from Bank5X, they always go externally. They got American lawyers to help them.” (Int. 78)

OA906 Bank5X: “At Bank5X, we got a *legal opinion from the U.S.*” (Int. 78, *emphasis added*)

OA907 [Bank70] “retained *U.S. qualified tax lawyers of an international law firm* to review the account dossiers of all other U.S. clients to determine if affirmative evidence of these clients’ tax compliance was on file.” (SOF70, *emphasis added*)

OA908 Bank70: "External counsel determined that a *Form W-9* was required for individual account holders, Form 5471 in case of controlled foreign corporations, and Form 3520-A in case of non-U.S. trusts or foundations. Where the external tax lawyers determined that the required form was not present in the Bank's dossiers, the Bank contacted the client by mail and asked that the client submit the missing form. The Bank additionally required that the client sign a declaration agreeing to the disclosure of their accounts to the IRS pursuant to applicable law. *The Bank's outside counsel was actively involved in drafting appropriate correspondence.*" (SOF70, 2015, *emphasis added*)

OA909 Bank70: "As an additional measure, [Bank70] retained *U.S. qualified tax lawyers of an international law firm to review the account dossiers of all other U.S. clients* to determine if affirmative evidence of these clients' tax compliance was on file." (SOF70, 2015, *emphasis added*)

OA910 Bank8X: “We said [right during the UBS case], now we need support, we see the problem, we do our internal investigation, we do that accompanied by *U.S. lawyers*... we then wrote to four, five major U.S. law firms and then got advice from a U.S. lawyer.” (Int. 56, *emphasis added*)

OA911 Legal advisor: “When [the banks] asked me back then, I told them not to touch the Americans.... I was always very afraid of the Americans.” (Int. 89)

C) Internationally oriented owners

OA912 Bank1X: “[The owner] reacted pretty quickly and, of course, the Swiss affiliate of their banks did as well.” (Int. 70)

OA913 Bank2X: “We said you simply have to *behave identically to the parent company*.” (Int. 59, *emphasis added*)

OA914 Bank2X: “We were also, in addition to Bank22, also represented in another bank or asset manager... They actually wanted to make a profit, but we clearly said that, as long as we were the majority shareholder, it was out of the question. That is not an issue for us.” (Int. 59)

OA915 Bank4X: “This has such an owner effect.” (Int. 54)

OA916 Bank5X: “…certainly also *inquiries from the German regulator*. And the German regulator has no access to the Swiss banks and has, I think, simply made inquiries there as well. The regulators, the politicians, the board members, they meet regularly at events. They talk to each other. It’s not always a hundred percent official. But, of course, that leads to the formation of an opinion, and that then also leads to actions and directives at the end.” (Int. 65, *emphasis added*)

OA917 Bank8X: “The Chinese wanted, or the English did not want that at all. And, of course, they didn't want it on the England-America axis either. So, rules were imposed on us that we didn't even know in this form in compliance.” (Int. 79)

Reinforcement of threat perceptions

<u>Reinforcement by internationally oriented legal advisors</u>

OA918 Bank8X: “And there I had, with our *U.S. attorneys*, I said, I have a very bad feeling about this. And I want to arm myself against that.” (Int. 56, *emphasis added*)

OA919 “I went to [University] in New York. I studied there and worked there. (…) I know the U.S. legal system well.” (Int. 2)

OA920 “I studied in the U.S.A. A relative of mine has a bank in the U.S.A. I know *the legal situation in the U.S.A*.” (Int. 3, *emphasis added*)

Reinforcement by internationally oriented associations

OA921 Bank18: “In early 2009, [Bank 18] attended a presentation organized by the [association of international banks] in Switzerland called "Do’s and Dont’s with US Customers."” (SOF18)

OA922 “[T]he legal compliance people from the foreign multinational banks were in the [association of international banks]. They observed current trends, regulatory, legal, etc., and discussed how their banks should react to such things. And when the UBS issue came up, they were dedicated to this issue. What does it mean? Can we do that, should we do that, do we want to do that? They knew each other well. They were relatively open with each other.” (Int. 86)

OA923 “Bankers went to the meetings of the [bank association]. We have been advised that this is an issue. Or bankers ... had *sensitivities with the US* because it related to them... *because of their experience*.” (Int. 82, *emphasis added*)

Reinforcement by internationally oriented owners

OA924 Bank4X: “[The owner] said: Look at this carefully. You need to protect yourself from this.” (Int. 88)

OA925 Bank5X: “The [foreign] *Group management had a lot of respect for these risks*.... has always reacted rather critically to Swiss management and always had an uneasy feeling somewhere. ... every Swiss bank has a Swiss board of directors... But if, of course, the owner looks at certain things in detail or makes additional stipulations, then of course, this has a very strong *influence on how you perceive risks*.” (Int. 65, *emphasis added*)

LOCALS **Reinforcement of territorial categorizations**

Reinforcement by domestically oriented advisors and networks

A) Reinforcement by authorities and actors making attribute-based territorial categorization

OA926 Bank6x: “*If the Swiss government* changes the rules, we comply with it.” (Interviewee; Bank62; SchweizerBank21/07/09, *emphasis added*)

OA927 “FINMA says: "We expect you to act within the law to comply"... So, acting within the law, meaning within Swiss law...” (Int. 87)

OA928 “FINMA looked at and investigated everything possible and impossible. But the problem of state aid was not yet in our genes at that time. It’s different now. But, at that time, it was like that.” (Compliance consulting of several local banks) (Int. 71)

OA929 “Directly to the foreign law, FINMA has said nothing. On the contrary, they say we don’t know. We are not competent there.” (Int. 71)

OA930 “FINMA had judged this wrong, legalistically and according to Swiss law.” (Int. 71)

OA931 “Then , one actually only looked at the published decisions by FINMA in the UBS Case. In the *FINMA documents*, there is no mention of this conspiracy.” (Int. 71, *emphasis added*)

OA932 “[I]t was assumed relatively strongly [by the Swiss regulator] that *on Swiss territory exclusively Swiss law applies*. (...).” (Int. 8, *emphasis added*)

OA933 “FINMA will not act as the «long arm» of a foreign regulator.” (DuPasquier & Fischer, 2010: 3)

OA934 Swiss regulators highlighted Swiss banks’ obligation to register as an investment advisor in the U.S., if they operated “*financial services in the U.S.*”. “A financial institution is then considered to be *operating in the U.S., if it uses U.S. jurisdictional means*, that is, email, fax, mail, phone, internet or other commercial instruments.” (GPK, 20100530: 3245, *emphasis added*)

OA935 Swiss regulators emphasized that Swiss banks’ crossborder business with U.S. clients came under scrutiny of U.S. regulators, in particular, *banks’ activities with “nexus” with U.S. soil, i.e. banks’ use of so-called “U.S. jurisdictional means”*. “These terms refer to U.S. concept of «*U.S. jurisdictional means*», which includes most communications (e.g., by telephone, by fax, by regular mail or by email) sent from outside into the USA. Travelling on an interstate highway in the USA also constitutes a use of a U.S. jurisdictional mean.” (DuPasquier & Fischer, 2010: 458, *emphasis added*)

OA936 In two meetings, FINMA “accepted” the conduct of a *Swiss bank* [that engaged in significant business with U.S. clients’ tax evasion], as the conduct was *not illegal under Swiss law*. (Hässig, 2008, *emphasis added*)

OA937 “[I]t was assumed relatively strongly that *on Swiss territory exclusively Swiss law applies*. (...) this was never much discussed.” (Int. 8, *emphasis added*)

OA938 Swiss regulators highlighted: “A problem arises, *if client advisors of Swiss banks travel on-site,* hence to the clients abroad, to render such services. They *then have to obviously fulfill the legal prescriptions of the target country.*” (GPK, 20100530: 3392, *emphasis added*)

OA939 Swiss regulators highlighted Swiss banks’ obligation to register as an investment advisor in the U.S., if they operated “*financial services in the U.S.*”. “A financial institution is then considered to be *operating in the U.S., if it uses U.S. jurisdictional means*, that is, email, fax, mail, phone, internet or other commercial instruments.” (GPK, 20100530: 3245, *emphasis added*)

OA940 Swiss regulators emphasized that Swiss banks’ crossborder business with U.S. clients came under scrutiny of U.S. regulators, in particular, *banks’ activities with “nexus” with U.S. soil, i.e. banks’ use of so-called “U.S. jurisdictional means”*. “These terms refer to U.S. concept of «*U.S. jurisdictional means*», which includes most communications (e.g., by telephone, by fax, by regular mail or by email) sent from outside into the USA. Travelling on an interstate highway in the USA also constitutes a use of a U.S. jurisdictional mean.” (DuPasquier & Fischer, 2010: 458, *emphasis added*)

OA941 The Swiss regulator's 2008 Annual Report states: "[T]he Banking Commission expects, as a matter of principle, that banking institutions active on a global scale *comply with the rules and regulations applicable in the countries in which such institutions conduct their business operations*." (SFBC AR 2008: 33, *emphasis added*)

OA942 FINMA *"*looked closely at all *[Swiss] wealth managers* (…) the FINMA said: 'Yes, in the end, according to *Swiss law*, it is not forbidden to do that (…) from a Swiss perspective, there was no conflict from a legal point of view." (Int. 8, *emphasis added*)

B) No legal advice sought / Reinforcement by legal experts with focus on CH law

OA943 Bank0X: "Our compliance consultant said, no, that wasn't a problem, because he looked after many banks, where he also continued to drive exactly this track." (Int. 62)

OA944 Bank38: "In May 2009, [Bank38] issued a directive requiring that the head of the premium banking department approve all new U.S.-domiciled clients, *prohibiting Bank employees from sending correspondence to the United States or accepting orders received by telephone, fax, or mail from the United States, and prohibiting U.S.-domiciled clients from initiating transactions through the e-banking system*. The Bank also placed these account holders in a restricted category requiring additional monitoring by the Bank's compliance department. *The Bank issued this directive after its auditor had approved it as consistent with U.S law* and after the Bank's executive board had confirmed its decision to continue advising U.S. customers and accepting new U.S. accounts." (SOF38, 2015, *emphasis added*)

OA945 Bank78 "did not seek any outside legal advice about whether or not their conduct would further assist U.S. customers in evading United States taxes. According to [Bank78] executives, *they did not believe outside legal advice was necessary.* In their view, the Bank could open accounts for U.S. taxpayers to give them a place to park their undeclared assets." (SOF78, 2015, *emphasis added*)

OA946 "And that always happens, of course, when you're on a board of directors and something like this happens, you go to *your in-house counsel* and say, take a look at this and tell me what you think about it. And then you go into these papers and then it becomes a little bit legalistic like that. And when you look at it legalistically, it has actually come to a relatively, a weak risk assessment. You might have judged it wrong, but legalistically right..." (Int. 71, *emphasis added*)

OA947 "I do remember that the advice we got *from our in-house lawyers* [and from a Swiss law firm], that was our main legal firm, was that there is no issue. There is no issue." (Int. 55, *emphasis added*)

OA948 "It always comes back to that feeling that legal, your auditors, your senior staff, are all saying the same message. They were saying that to me as the CEO. They must have been saying the same to their head offices around the world saying, "No, not to worry."" (Int. 55)

OA949 "My legal advisors said the US does not have this right." (Int. 55)

OA950 "The consultants, the local law firms, they all thought in the same manner. They all thought that the Swiss law primed. Their argument was that this is-- and that's where they were wrong. "This is Swiss policy."" (Int. 55, *emphasis added*)

OA951 "I do remember very well around 2008, with the UBS cases, (...) that I asked my legal counsel, "Are there any issues?" I was reassured, "No. No. No. No. *Within Swiss law, we're doing everything... they probably considered the Swiss integrity*. (...) people *with a Swiss legal training* perpetuated the views that *Switzerland is neutral and should not be attacked by the US* (...) But in a way, you'd probably say that maybe the Swiss legal industry should have woken up a bit earlier to some of those issues... around 2008 but I think they kept very muted about it for a long time." (Int. 10, *emphasis added*)

OA952 "[T]heir *legal counsels*, in-house and external, must have given them comfort that what they were doing is fine. I do remember very well around 2008, with the UBS cases, (…) that I asked my legal counsel, "Are there any issues?" I was reassured, "No. No. No. No. *Within Swiss law, we're doing everything… they probably considered the Swiss integrity*. (…) people *with a Swiss legal training* perpetuated the views that *Switzerland is neutral and should not be attacked by the US* (…) But in a way, you'd probably say that maybe the Swiss legal industry should have woken up a bit earlier to some of those issues… around 2008 but I think they kept very muted about it for a long time". (Int. 10, *emphasis added*)

OA953 "*'No, no, don't worry; we have Swiss laws, Swiss banking secrecy...*' And I am Dr. [name], lawyer in Zurich, and I can tell you that because of Swiss law… you can't beat it. (…) 'Don't worry. *We've got a constitution. We've got law*, etc. And the Americans will never be able to do anything.'" (Interviewee 11, *emphasis added*)

OA954 "[A]n executive of Wegelin told the team leaders (...) that Wegelin could charge high fees to its new U.S. taxpayer-clients because they were afraid of criminal prosecution in the United States…. This advice by a member of Wegelin's senior management is all the more egregious by virtue of that *executive's extensive training as a lawyer and experience in law enforcement*. See PSR ¶ 41." (Rakoff In DOJ, 20130225: 6, *emphasis added*)

C) Reinforcement by domestically oriented associations and ties to other banks

OA955 Bank1X: "One was Banking Association here in [City] (laughs)... The problem at that time was to get the know-how at all, so likewise yes. For us as a local bank, the question is probably how do we get the relevant information at all? And, of course, also in good time and how do we find out what developments there are in general in these problem areas?" (Int. 84)

OA956 "[The CEO of Bank X and president of a bank association] ran around [in the association] there and said that we only had to refer to Swiss law." (Int. 86)

OA957 Bank41: "My wife worked at [local bank] as an assistant. And I was also in contact with the former General Counsel of [local bank]." (Int. 74)

Reinforcement of safety perceptions

<u>Reinforcement by domestic regulator</u>

OA958 "With the globalization and the internationalization of criminal standards, our business principles have fundamentally changed. However, politics has not fulfilled its role as an early warning system." (Interviewee, DerBund03/10/09)

OA959 "The Federal Council at the time said that banking secrecy would never be betrayed, over my dead body. And that already gave the feeling of security." (Int. 64)

OA960 "They were in the same soup, in the same network, and exchanged information and kind of reassured themselves that nothing can happen. That they can just close the fences." (Int. 82)

<u>Reinforcement by domestically oriented legal advisors</u>

OA961 Bank14: "In September 2008, [Bank14] outsourced its compliance function to an external consulting firm based in Switzerland ("Consulting Firm #I"). A principal of Consulting Firm #I ("Compliance Consultant #I") commenced an internal investigation of the Bank's U.S. cross-border business. (...) Compliance Consultant #2 no longer recommended that the Bank obtain an expert opinion on that topic and concluded in a later report that the situation could be "*considered problem-free to a large extent if the activities are performed in Switzerland."* (...) On September 25, 2009, at the request of [Bank14], another external consulting firm based in Switzerland ("Consulting Firm #2") (...) was at the meeting and stated that he could recommend continuing foreign customer accounts" as long as the Bank implemented the recommendations in his report. (...) Bank Executive #I also stated that "there is practically no risk *if U.S. customers travel to Switzerland and a customer account is handled locally*," and added that he could imagine "accepting new seriously high net worth customers from the USA at [Bank14] provided the basic legal requirements are complied with." (SOF14, 2015, *emphasis added*)

OA962 Bank38: "In May 2009, [Bank38] issued a directive requiring that the head of the premium banking department approve all new U.S.-domiciled clients, *prohibiting Bank employees from sending correspondence to the United States or accepting orders received by telephone, fax, or mail from the United States, and prohibiting U.S.-domiciled clients from initiating transactions through the e-banking system*. The Bank also placed these account holders in a restricted category requiring additional monitoring by the Bank's compliance department. *The Bank issued this directive after its auditor had approved it as consistent with U.S law* and after the Bank's executive board had confirmed its decision to continue advising U.S. customers and accepting new U.S. accounts." (SOF38, 2015, *emphasis added*)

OA963 "I do not recall that any lawyer made that difference of territorial, extraterritorial at that time. They just said, "It's nothing to worry about."" (Int. 55)

OA964 "[T]heir legal counsels, in-house and external, have given them *comfort* that what they were doing is fine." (Int. 10, *emphasis added*)

EPISODE2 **REINFORCEMENT BY EPISTEMIC AUTHORITIES**

LOCALS **Reinforcement of territorial categorizations**

<u>Reinforcement of adaptation by advisors and networks</u>

A) Adapted thinking of Swiss regulator

OA965 "From a criminal perspective, banks and their employees can make themselves *liable to prosecution for facilitating tax offenses*. This can *even apply, if they are only active in Switzerland*.". (Interviewee, FINMA, Finanz&Wirtschaft24/03/10, *emphasis added*)

OA966 "From a *foreign law perspective*, it is very easy to challenge Swiss banking secrecy, in that one says, the conduct is *conspiracy*, it is indictable. (…) This criminal perspective through the *conspiracy lens*… is a possibility in foreign law…" (Int. 8, *emphasis added*)

OA967 "This went clearly beyond the previously defended Swiss "territoriality" principle" by Swiss authorities". (Int. 8, *emphasis added*)

OA968 Before the Wegelin case, "one did not arrive to the point to say: 'And this means *you cannot accept untaxed US customers*. This step was taken in 2012 after this Wegelin indictment. (...) And then only after Wegelin they said: 'Listen, this will not work anymore (…) In the end, the notion of *knowingly accepting funds or tax money from a U.S. customer by a bank constitutes a violation of U.S. law, independently of whether it has happened abroad or not*'." (Int. 8, *emphasis added*)

OA969 Swiss regulators *waived* the supremacy of Swiss law over banks in Switzerland". (FDJP14/04/10, *emphasis added*)

OA970 "And then from 2011 and 12, Switzerland as a country had to basically go through a similar kind of thinking process, which was do we want to pursue with banking secrecy that could be misused? But then, Switzerland would have cut itself off the international system." (Int. 67)

OA971 "But the wake-up call then also came with this circular, in that FINMA said, be careful in cross-border business. (Int. 88)

OA972 The arbitrage attitude is no longer possible. So, there is *this circular from FINMA*, which said that this is a new business risk. I would have to pay attention to that." (Int. 88, *emphasis added*)

B) Convergence of legal expertise among legal advisors

OA973 Bank0X: "After the Wegelin case, we banks in [a Swiss region] all got together and started working with [an internationally oriented law form]. And [this law firm] then in turn had in a *lawyer in New York*." (Int. 62, *emphasis added*)

OA974 Bank13: “management and the Bank's *external compliance consultant* conducted a comprehensive review [in line with U.S. law].” (SOF13, 2015, *emphasis added*)

OA975 Since 2013, Bank14 “formed an Operational Committee consisting of [Bank14] *representatives and U.S. and Swiss law firm partners (...)* Along with its outside advisors, the Bank established a *multi-tiered review protocol to identify and analyze all U.S. Related Accounts."* (SOF14, 2015, *emphasis added*)

OA976 Bank 16 “engaged *U.S. and Swiss counsel* as well as forensic accounting experts to conduct an internal review in order to identify and collect data and information regarding its U.S.-taxpayer accounts and to examine its conduct in relation to such accounts.” (SOF16, 2015, *emphasis added*)

OA977 [Bank19] “retained an outside firm to assist the Bank in identifying *U.S. clients* and determining what documentation the Bank had already collected on those clients.” (SOF19, 2015, *emphasis added*)

OA978 Bank21’s “management decided that the Bank should proactively monitor the tax-compliance of new clients. (...) It hired a *Swiss law firm and a U.S. accounting firm* to reach out to U.S. account holders to persuade them *to come into compliance with U.S. tax law*.” (SOF21, 2015, *emphasis added*)

OA979 Bank5X: “And then we basically kind of engaged *U.S. lawyers*, and then it was very much a guy called [Name], who was a colleague of the CEO who was the front man for that.” (Int. 60, *emphasis added*)

OA980 Bank64: “The Bank's management was fully aware that the Zurich branch in particular had onboarded numerous structured accounts, and openly discussed its ramifications at a February 2012 board meeting after an internal audit *by an outside accounting group*. The independent report stated: "The Zurich branch serves mainly foreign private clients. The range of clients of the Zurich branch includes U.S. clients. However, pursuant to the bank’s strategy, these clients are not among the target group of the bank." The report contained a breakdown of various categories of U.S. Related Accounts by branch, demonstrating that Zurich controlled nearly 75% of the bank's U.S. Related Accounts in terms of assets under management.” (SOF64, 2015, *emphasis added*)

OA981 Bank77: “On May 31, 2012, the *Bank's external auditor* furnished its annual report on regulatory matters to the Bank's Board of Directors and to the Bank's supervising regulatory agency, FINMA.” (SOF77, 2015, *emphasis added*)

OA982 The Wegelin case “shows that the US, in order to tackle tax evasion, is willing to prosecute (…) any [accomplices] helping clients to open accounts and set up offshore structures to hide money from the IRS.” (Troller et al., 2013)

Reinforcement of threat perceptions

<u>*Reinforcement of threat perception by domestic regulator*</u>

OA983 Bank1X: “The paper where *FINMA* pointed out that there are risks from cross-border banking. It is no longer sufficient to rely only on Swiss law, but you may also need to *take into account the supervisory, tax and criminal law of other countries*.” (Int. 84, *emphasis added*)

OA984 “This step was taken in 2012 after this Wegelin indictment. (...) And then only after Wegelin [*FINMA*] said: ‘Listen, this will not work anymore (…) In the end, the *notion of knowingly accepting funds or tax money from a U.S. customer by a bank constitutes a violation of U.S. law*, independently of whether it has happened abroad or not’.” (Int. 8, *emphasis added*)

OA985 “From a criminal perspective, banks and their employees can make themselves *liable to prosecution for facilitating tax offenses*. This can *even apply, if they are only active in Switzerland*.”. (Interviewee, FINMA, Finanz&Wirtschaft24/03/11, *emphasis added*)

OA986 FINMA “calls on [Swiss] institutions to also comply with *foreign supervisory law* and define an appropriate service model for each target market.” (FINMA, 22/10/11, *emphasis added*)

OA987 “From a criminal perspective, banks and their employees can make themselves *liable to prosecution for facilitating tax offenses*. This can *even apply, if they are only active in Switzerland*… financial institutions, in the context of *assuring an appropriate management organization*, have to record, limit and supervise [foreign law]”. (Interviewee, FINMA, Finanz&Wirtschaft24/03/11, *emphasis added*)

OA988 In a position paper, *FINMA* emphasizes that “it would be indispensable that [Swiss banks] produced in-depth analyses of the cross-border financial services business (…) The paper reports on a Swiss subsidiary of a foreign bank, which *from Switzerland serviced clients beyond the Swiss border*. (…) The transactions have been concluded at the *place of residence of the client*, but *booked at the place of domicile of the bank in Switzerland*. The bank ‘*lacked the necessary sensitivity*’.” (DerBund23/10/11, *emphasis added*)

OA989 “From a criminal perspective, banks and their employees can make themselves *liable to prosecution for facilitating tax offenses*. This can *even apply, if they are only active in Switzerland*… financial institutions, in the context of *assuring an appropriate management organization*, have to record, limit and supervise [foreign law]”. (Interviewee, FINMA, Finanz&Wirtschaft24/03/11, *emphasis added*)

<u>*Reinforcement of threat perception by legal advisors*</u>

OA990 Bank14: “... Along with its outside advisors, the Bank established a *multi-tiered review protocol to identify and analyze all U.S. Related Accounts.*” (SOF14, 2015, *emphasis added*)

OA991 Bank19: “In July 2013, based on the *evaluation of the outside firm*, the Bank established a CrossBorder Team, whose mandate included closing many foreign accounts and all U.S. Related Accounts. Foreign-domiciled account holders were retained (...) only if those account holders signed a declaration of proper taxation of their assets with the Bank and a waiver of Swiss banking secrecy.” (SOF19, 2015, *emphasis added*)

OA992	“Then, of course, there was a convergence in legal advice, because it was absolutely clear, that you have a serious problem if you have black money in the business model.” (Int. 88)

ONLINE APPENDIX II: ALTERNATIVE EXPLANATIONS

We conducted supplementary analyses to account for alternative explanations of our findings.

Comparison of the two enforcement events

First, we drew on the deterrence and coercion literatures to assess whether there were important differences between the two U.S. law enforcement events (see Table 1). The *severity of sanctions* levied in law enforcement may be an alternative driver of the unprosecuted organizations' compliance decisions (Nagin, 2013). At USD 780 m and USD 74 m, representing 4.6% and 6.1% of the targets' total amount of undeclared U.S. assets, the sanctions' severity was high across the two events. Wegelin was indicted, while UBS eventually reached a deferred prosecution agreement with the DOJ. In both instances, however, these prosecution events hit the firms very hard.[1]

Table 1. Comparison of the UBS and the Wegelin Enforcement Events

Indicators	Enforcement event 1 (UBS)	Enforcement event 2 (Wegelin)
The sanctions' severity	High	High
The U.S. regulators' access to evidence	High	High
The prosecution events' salience	High	High
The U.S. regulators' coercive capacity	High	High
The U.S. regulators' clarity of enforced territorial category	Low	High

Further, in both law enforcement events, several executives were individually charged. Two senior UBS executives were arrested in the U.S.[2] (SI14/11/04). While these factors may have been

[1] In 2008, the year that the U.S. fine was charged, UBS had been fully hit by the financial crisis, resulting in a net loss of CHF21.3 b. The bank had to be saved by emergency law and an emergency loan (SI14/11/04). The amount of the U.S. penalty was regarded as "shocking" by the unprosecuted banks (e.g. Int. 91). In contrast, Wegelin was highly profitable in 2012 and could pay the U.S. fine without facing existential threats. Nonetheless, the U.S. indictment endangered the bank, because its partners threatened to withdraw from it. The U.S.-related business was phased out and Wegelin continued its non-US-related business under the brand name "Bank Notenstein" (Schönig and Straumann, 2023).

[2] The two UBS executives included a member of the bank's top management team, the Swiss-based global head of private banking. He was arrested while on a business trip in Italy and was extradited to the U.S.

necessary, our data suggest that the perceived prosecution risk, as triggered by the unprosecuted organizations' territorial categorizations (rather than severity) explained the differences in organizations' responses.

We further assessed the *regulators' access to evidence* (cf. Short and Toffel, 2010; Short, 2013). Across the enforcement events, the U.S. regulators gained extensive evidence through revelations either by whistleblowers or by clients who benefitted from rewards given by the U.S. government. Comparing the *salience of prosecution events* (e.g. Thornton, Gunningham, and Kagan, 2005), we found that both events were highly mediatized. Finally, the *U.S. regulators' coercive capacity* was high in both events, because they controlled banks' access to the U.S. dollar (cf. DiMaggio and Powell, 1983; Emmenegger and Eggenberger, 2018). This qualitative comparison suggests that the observed differences in the banks' prosecution risk perceptions and compliance decisions could not be explained only by these factors. They did have a role, as they were present and high in both events. Thus, they may be seen as necessary but insufficient factors for explaining the observed differences in the general deterrence effects we observed. Unlike most deterrence research, our study points to the cultural – rather than only material – dimensions of law enforcement events.

Further, we sought to rule out that the U.S. regulators changed their theory of enforcement jurisdiction between the two events, relying on territoriality jurisdiction (i.e. attribute-based territorial categorization) in the UBS case and on extraterritorial jurisdiction (i.e. goal-based territorial categorization) in the Wegelin case. First, legal scholars (e.g. Verdier, 2019; 2020) confirmed that, in *both* enforcement events, the conspiracy statute (18 U.S.C. §371) was applied extraterritorially for violations of U.S. law committed *in Switzerland*. Second, evidence – e.g. by the federal prosecutor of Wegelin – suggests that, already in the UBS enforcement, the U.S. regulators' intention was to deter *all* Swiss banks (not only those with a U.S. nexus) from committing conspiracies no matter where they were committed (Schönig and Straumann, 2023).

Further, we checked the possibility that changes in U.S. law explained the observed differences in compliance decisions. The Foreign Accounts Tax Compliance Act (FATCA), which introduced a shift to mandatory reporting by foreign financial institutions, was ratified in a new bilateral agreement between the U.S. and Switzerland in 2013 and came into force in 2014 (Fedlex, 2013) – both after our observation window. Our data suggests that, with four exceptions that anticipated its ratification, FATCA did not influence the unprosecuted organizations' compliance decisions.

We ensured that the Swiss regulator's influence on unprosecuted organizations (and especially on the 'locals') was predominantly one of epistemic authority, ruling out the possibility that legal changes explained differences in their compliance decisions. In our observation window, Switzerland maintained the distinction between "tax fraud" and "tax evasion", regarding only the former as criminal offense and tolerating banks' facilitation of their clients' tax evasion. While Switzerland's double taxation treaty with the U.S. was revised after both enforcement events (in 2009 and in 2012), these revisions only focused on accelerating the administrative process with which information on U.S. clients who had committed "tax fraud and the like" was exchanged with the U.S. regulator for banks already under investigation, i.e. for which incriminating evidence existed (Fedlex, 2009). Hence, these revisions focused predominantly on bank clients rather than on the banks themselves.

Comparison of the compliant and non-compliant organizations

Second, we assessed whether there were key differences between the compliant and the non-compliant organizations following the UBS enforcement event. We crafted measures for potential alternative explanations based on the literatures on deterrence, social comparison, and normative institutional forces, conducting tests of group differences (for the results, see Table 2; for an overview over the measures, see Table 3).

Table 2. The Compliant vs. Non-Compliant Banks after the UBS Event

Indicators	Group difference test results[3]		
	**Compliant banks (N=42)**	**Non-compliant banks (N=51)**	**p-value**
**Deterrence**			
Percentage of U.S. assets	0.03 (0.02, 0.06)	0.03 (0.01, 0.06)	0.367
Unknown	*2*	*10*	
Percentage of U.S. accounts	0.02 (0.01, 0.04)	0.02 (0.01, 0.03)	0.641
Unknown	*9*	*16*	
**Social comparison**			
Status similarity			>0.999
Similar	18 (54.5%)	23 (54.8%)	
Dissimilar	15 (45.5%)	19 (45.2%)	
Unknown	*9*	*9*	
Business model similarity			0.075
Similar	5 (11.9%)	14 (27.5%)	
Dissimilar	37 (88.1%)	37 (72.5%)	
Geographic similarity	87.4 (0.00, 276.60)	86.80 (0.00, 152.35)	0.351
**Normative forces**			
Legal status			0.370
Private	38 (90.5%)	42 (82.4%)	
Public	4 (9.5%)	9 (17.6%)	
**Further organizational characteristics**			
Firm age	79.50 (36.00, 152.00)	102.00 (51.00, 160.00)	0.418
Firm size	2,313.55	1,741.01	0.622
Unknown	(557.24, 8,871.47)	(438.73, 8,369.66)	
	8	*9*	
Prominent firms	8 (19.0%)	7 (13.7%)	0.576
Ownership type			0.085
Private	36 (85.7%)	35 (68.6%)	
Public	6 (14.3%)	16 (31.4%)	

Following the deterrence literature (Paternoster and Bachman, 2012; Nagin, 2013), we assessed the severity of the unprosecuted organizations' offenses with two measures: banks' *percentage of U.S. assets* and *percentage of U.S. accounts* were comparable across the two groups (see Table 2). Other triggers of unprosecuted organizations' prosecution risk perceptions, e.g., whether they thought they could remain "under the radar" of the U.S. regulator (e.g. Schell-Busey et al., 2016), were less

[3] Results were based on bivariate analyses using the nonparametric Wilcoxon rank sum exact test. No major differences were found with the parametric Welch two-sample t-test. For some variables, the data were not available for all banks. We reported the number of banks per group for which the data were *unknown* for a given variable. Overall, the cosmopolitans were rather in private ownership (p=0.085) and of dissimilar business model type (p=0.075) than UBS. Yet, these differences were insufficient for explaining the observed results. Further, we conducted binary logistic regressions with a sub-sample of 53 banks to test for membership in the non-compliant group of banks. Besides firm age (p=0.028), there were no significant results at the 5% and 10% levels.

prominent in our data (except for 4 cosmopolitans and 3 locals, as mentioned in our findings).

Table 3. Overview over the Indicators Used in the Group Comparison

Indicators	Operationalized in terms of…
**Deterrence**	
Percentage of U.S. assets	… the amount of U.S. assets over the total AUM.
Percentage of U.S. accounts	… the amount of U.S. accounts over the total AUM.
**Social comparison**	
Status similarity	… the bank's market share in the industry (Yiu, Xu, and Wan, 2014). A unprosecuted organization is in the high-status group if it is above or equal to the industry median; otherwise, it is in the low-status group. An organization is considered of similar status if it is a member of the same group as the enforcement target.
Business model similarity	… whether the unprosecuted organization's and the target's business models are of the same type following the prevailing business model categories in the industry (i.e. pure player, hybrid, cantonal, universal bank) (cf. Barreto and Baden-Fuller, 2006).
Geographic similarity	… the distance (km) between the headquarters between the target and the unprosecuted banks (Davis and Greve, 1997; Peteraf and Shanley, 1997).
**Normative forces**	
Legal status	… the sector the bank is part of (Edelman, 1992), specifically, whether it is a public or private law institution.
**Further organizational characteristics**	
Firm age	… years since founding. The institutional research suggests that older firms may be more inert and therefore more resistant to comply with novel legal demands (Dobbin and Sutton, 1998). In contrast, deterrence research suggests that younger firms may be more likely to engage in corporate fraud, because managers are under pressure to meet earnings expectations (Beasley, 1996).
Firm size	… the bank's total AUM. On the one hand, it has been positively related to corporate fraud (Arthaud-Day et al., 2006). On the other hand, large firms are more likely to comply with the law, because they may be more subject to societal pressures and because they can more easily afford regulatory costs (Edelman, 1992).
Prominent firms	… whether the bank appeared in the leading Swiss private banking rating by the Swiss magazine *Bilanz*. Because prominent firms may be more salient, they may be more subject to societal pressures (Mishina et al., 2010) and therefore more likely to comply with the law.
Ownership type	… whether a bank is privately or publicly owned. A firm's governance structure may lead managers to prioritize returns over compliance (Beasley, 1996; cf. Barreto and Baden-Fuller, 2006).

The literature on social comparison, specifically on reference groups, advances the similarity level between prosecuted and unprosecuted organizations, specifically status similarity (Yiu, Xu, and Wan, 2014), as a key predictor of the extent to which a targeted firm acts as a reference point for unprosecuted organizations. With greater similarity between the two, experiences of the target become more relevant to the unprosecuted organization (Massini, Lewin, and Greve, 2005).

Unprosecuted organizations are then more likely to select the target as a reference point to assess the likelihood of getting caught and punished for fraudulent behaviors (Stafford and Warr, 1993). We crafted three measures to assess the similarity level: *status*, *business model*, and *geographic similarity* (cf. Davis and Greve, 1997; Barreto and Baden-Fuller, 2006; Yiu, Xu, and Wan, 2014).

Finally, we assessed further company characteristics that potentially affect their compliance decisions. We assessed the banks' proximity to the public sphere (i.e. the norms and culture surrounding the state) based on its *legal status* (Edelman, 1990; 1992), the firms' *age*, *size*, *prominence*, and *ownership type* (see Edelman, 1992; Gunningham, Thornton, and Kagan, 2005; Yiu, Xu, and Wan, 2014).

Overall, as Table 2 shows, we found no significant differences in these variables that are in line with prior theory. In contrast to prior theory, privately (rather than publicly) owned firms, and firms with a dissimilar (rather than similar) business model to UBS complied faster with U.S. law. We could thus rule out the possibility that the two groups and the observed differences in general deterrence across the groups are driven by the abovementioned explanations, rather than by the cultural-cognitive mechanism we found. These supplementary analyses strengthened our confidence that our findings cannot be answered with existing theory, further corroborated our proposed theory of general deterrence across borders, and further legitimated our inductive and grounded theory-building approach (cf. Edmondson and McManus, 2007).

REFERENCES

Barreto, I. and C. Baden-Fuller

2006 "To conform or to perform? Mimetic behaviour, legitimacy-based groups and performance consequences." Journal of Management Studies, 43: 1559-1581.

Davis, G. F. and H. R. Greve

1997 "Corporate elite networks and governance changes in the 1980s." American Journal of Sociology, 103: 1-37.

DiMaggio, P. J. and W. W. Powell

1983 "The iron cage revisited: Institutional isomorphism and collective rationality in organizational fields." American Sociological Review, 48: 147-160.

Edelman, L. B.

1990 "Legal environments and organizational governance: The expansion of due process in the american workplace." American Journal of Sociology, 95: 1401-1440.

Edelman, L. B.

1992 "Legal ambiguity and symbolic structures - organizational mediation of civil-rights law." American Journal of Sociology, 97: 1531-1576.

Edmondson, A. C. and S. E. McManus

2007 "Methodological fit in management field research." Academy of Management Review, 32: 1155-1179.

Emmenegger, P. and K. Eggenberger

2018 "State sovereignty, economic interdependence and u.S. Extraterritoriality: The demise of swiss banking secrecy and the re-embedding of international finance." Journal of International Relations and Development, 21: 798-823.

Fedlex

2009 "Abkommen zwischen der schweizerischen eidgenossenschaft und den vereinigten staaten von amerika zur vermeidung der doppelbesteuerung auf dem gebiete der steuern vom einkommen." S. Confederation, (ed.), 0.672.933.61. Switzerland: Fedlex.

Fedlex

2013 "Fatca-gesetz: Bundesgesetz über die umsetzung des fatca-abkommens zwischen der schweiz und den vereinigten staaten." S. Confederation, (ed.), 672.933.6. Switzerland.

Gunningham, N., D. Thornton, and R. A. Kagan

2005 "Motivating management: Corporate compliance in environmental protection." Law & Policy, 27: 289.

Massini, S., A. Y. Lewin, and H. R. Greve

2005 "Innovators and imitators: Organizational reference groups and adoption of organizational routines." Research Policy, 34: 1550-1569.

Nagin, D. S.

2013 "Deterrence: A review of the evidence by a criminologist for economists." Annual Review of Economics, 5: 83-105.

Paternoster, R. and R. Bachman

2012 "Perceptual deterrence theory." In F. T. Cullen and P. Wilcox (eds.), The oxford handbook of criminological theory: Oxford Handbooks Online.

Schell-Busey, N., S. S. Simpson, M. Rorie, and M. Alper

2016 "What works?: A systematic review of corporate crime deterrence schell-busey et al. Corporate crime deterrence." Criminology and Public Policy, 15: 387-416.

Schönig, D. and T. Straumann

2023 Paria inter pares - das ende der bank wegelin. Bern: Stämpfli Verlag.

Short, J. L.

2013 "Competing normative frameworks and the limits of deterrence theory: Comments on baker and griffith's ensuring corporate misconduct." Law & Social Inquiry, 38: 493-511.

Short, J. L. and M. W. Toffel

2010 "Making self-regulation more than merely symbolic: The critical role of the legal environment." Administrative Science Quarterly, 55: 361-396.

Stafford, M. and M. Warr

1993 "A reconceptualization of general and specific deterrence." Journal of Research in Crime and Delinquency, 30: 123-135.

Thornton, D., N. Gunningham, and R. A. Kagan

2005 "General deterrence and corporate environmental behavior*." Law & Policy, 27: 262-288.

Verdier, P.-H.

2019 "The new financial extraterritoriality." The George Washington Law Review, 87: 239-314.

Verdier, P.-H.

2020 Global banks on trial: U.S. Prosecutions and the remaking of international finance. U.K.: Oxford University Press.

Yiu, D. W., Y. Xu, and W. P. Wan

2014 "The deterrence effects of vicarious punishments on corporate financial fraud." Organization Science, 25: 1549-1571.
